# Proteomic Approach to Reveal the Proteins Associated with Encystment of the Ciliate *Euplotes encysticus*

**DOI:** 10.1371/journal.pone.0097362

**Published:** 2014-05-16

**Authors:** Jiwu Chen, Xiuxia Gao, Bangzheng Wang, Fenfen Chen, Na Wu, Yuanyuan Zhang

**Affiliations:** School of Life Sciences, East China Normal University, Shanghai, China; Russian Academy of Sciences, Institute for Biological Instrumentation, Russian Federation

## Abstract

In order to identify and reveal the proteins related to encystment of the ciliate *Euplotes encysticus*, we analyzed variation in the abundance of the proteins isolated from the resting cyst comparing with proteins in the vegetative cell. 2-D electrophoresis, MALDI-TOF MS techniques and Bioinformatics were used for proteome separation, quantification and identification. The comparative proteomics studies revealed 26 proteins with changes on the expression in the resting cysts, including 12 specific proteins and 14 differential proteins. 12 specific proteins and 10 out of the 14 differential proteins were selected and identified by MALDI-TOF MS. The identified specific proteins with known functions included type II cytoskeletal 1, keratin, Nop16 domain containing protein, protein arginine n-methyltransferase, epsilon-trimethyllysine hydroxylase and calpain-like protein. The identified differential proteins with known functions included Lysozyme C, keratinocyte growth factor, lysozyme homolog AT-2, formate acetyltransferase, alpha S1 casein and cold-shock protein. We discussed the functions of these proteins as well as their contribution in the process of encystment. These identified proteins covered a wide range of molecular functions, including gene regulation, RNA regulation, proteins degradation and oxidation resistance, stress response, material transport and cytoskeleton organization. Therefore, differential expression of these proteins was essential for cell morphological and physiological changes during encystment. This suggested that the peculiar proteins and differential proteins might play important roles in the process of the vegetative cells transforming into the resting cysts. These observations may be novel findings that bring new insights into the detailed mechanisms of dormancy.

## Introduction

Ciliates are eukaryotic unicellular organisms with a highly differentiated cellular pattern. Vegetative cells of many ciliates can differentiate into resting cysts under unfavourable situations, such as starvation, temperature stress, high population density and so on. Encystment is a superior strategy for ciliates to survive in adverse environmental conditions. Encystment appears now of common occurrence in free-living ciliates and inevitable stage of parasitic ciliates [1].

Ciliate encystment involves drastic morphological and physiological changes, including a drastic decrease of cellular volume, the presence of partially permeable barriers (cyst walls) which are composed of distinct cyst wall layers derived from different precursors [2], [3], organelles clustering as a consequence of cytoplasmic dehydration [4], [5], a high autophagic activity and drastic nuclear changes [6], [7]. At present, many data of encystment have been accumulated at microscopic and submicroscopic level. However, biochemistry and molecular mechanisms of the ciliate encystment are still poorly known and a more extensive analysis at molecular level is absolutely necessary. Moreover, studies on the encystment at molecular level predominantly focus on parasitic protozoa, such as ***Giardia*** **and** ***Entamoeba*** [8], [9]. And studies on free-living ciliates like *Euplotes encysticus* encystment at molecular level are much fewer. More noteworthy is that parasitic protozoa encyst in order to avoid being digested by host, while free-living ciliates encyst so as to cope with adverse environmental conditions. Therefore, molecular mechanism and protein expression of these two organisms are significantly different during encystation. What's more, the protein events behind the free-living ciliates encystment are little known. In order to analyze the involvement of proteins differential expression in the encystment process of free-living ciliates, we used *Euplotes encysticus* as experimental material, using comparative proteomics strategy to identify and compare the differences of proteins expression between the resting cysts and the vegetative cells. Here we just considered the change of proteins expression levels, which was one aspect of ciliate encystment. The aim of this research was to reveal the proteins associated with encystment of the ciliate *Euplotes encysticus*.

## Materials and Methods

### Vegetative Cell Culture and Encystment Induction

Hypotrich ciliate *Euplotes encysticus* was used as experimental material because of its easy culture and induction. The cells were cultured in 10 cm glass petri dishes with sterilized pond water and the food organism *Chilomonas paramecium* at 25°C.

The vegetative cells were fed once every other day in the first few days and then fed twice each day when they generally multiplied faster. When vegetative cells were cultured to a high density, half (approximately 2×10^5^) of the cells were taken and stimulated to form resting cysts, which were mainly through starvation accompanied with low temperature.

### Extraction and Purification of Protein Samples

Respectively, approximately 2×10^5^ resting cysts and vegetative cells were filtered and concentrated by filter paper. Then the concentrated resting cysts and the vegetative cells were transferred into 1.5 mL centrifuge tube, respectively. The vegetative cells were centrifuged at 5000×g for 8 min at 4°C and the resting cysts were centrifuged at 2000×g for 5 min at 4°C. The supernatant was discarded and the harvested cells were mixed with 0.3 mL cell lysis buffer (4% SDS, 1 mM DTT, 150 mM Tris-HCl, pH 8; protease inhibitors). The suspension was kept for 10 min at room temperature, and then it was subjected to continued sonication treatment to ensure adequate lysis in an ice-bath 15 times, each time 30 s with a 30 s interval. These two samples were centrifuged at 14,000×g for 20 min and the precipitation was discarded. Repeat this step twice to remove the impurity. Finally, the supernatants were obtained as the total proteins samples of resting cysts and vegetative cells. Ice-cold acetone (5∶1) was added into the protein solutions to precipitate proteins at −20°C overnight, and then the proteins precipitated were centrifuged at 12000×g for 45 min and air-dried. 200 mg dried protein samples were dissolved in 0.5 mL 2D proteins extract buffer and sonicated for 3 min on ice. The protein extracts were centrifuged at 12000×g for 45 min and the supernatant were taken. The supernatants were filtered by 0.22 µm filter membrane and the clarified protein solutions were obtained. The protein concentration in each sample solution was determined using the non-interference type protein assay Kit (Sangon Biotech Company). Each subpackage sample was 80 µg and kept at −80°C before used.

### Two-Dimensional Gel Electrophoresis (2-DE) and Image Analysis

The individual samples (80 µg of proteins) were loaded during rehydration of IPG strips in 450 µL (total volume) of IEF buffer. IEF was performed using 24 cm, non-linear, pH 3–10, immobilized pH gradient (IPG) strips (GE Healthcare). IPG strips (50 µA/IPG strip) were run in an IPGphor system (GE Healthcare). The running conditions for IEF were as follows: 12 h at 30 V, 1 h at 500 V, 1 h at 1000 V, 8 h at 8000 V, 500 V for 4 h. After IEF, the strips runed in the second dimension (SDS-PAGE). Prior to SDS-PAGE, the focused strips were incubated in equilibration (EQ) solution (6 M urea, 50 mM Tris–HCl, pH 8.8, 2% SDS, 30% glycerol) containing 1% dithiothreitol (DTT, Sigma-Aldrich) and subsequently in EQ solution containing 2.5% iodoacetamide (Sigma-Aldrich) for 15 min and immediately applied on the top of 12% polyacrylamide gels. SDS-PAGE was performed using Ettan-DALT-Sbx system (GE Healthcare) with 15 mA/gel for the first 30 min and 30 mA/gel for the remaining separation. After the second dimension, gels were stained with silver according to Shevchenko had reported [10]. Briefly, the gels were fixed in 30% ethanol and 10% acetic acid and then sensitized in 0.02% sodium thiosulfate. The staining was performed in 0.1% silver nitrate. Dried 2-D gels were scanned with an Image Scanner (GE Healthcare), protein spots were quantified and numbered using the PDQuest 8.0 software (Bio-Rad) and checked manually to eliminate artifacts due to gel distortion, abnormal silver staining or poorly detectable spots. After background subtraction, normalization and matching, the spot volumes in gels from vegetative cells (as control) were compared with the matched spot volumes in gels from the resting cysts. The protein level of each spot was expressed as a percentage of total spot volume in the 2-DE gels. Comparison of test spot volumes with corresponding standard spot volumes gave a standardized abundance for each matched spot and values were averaged across triplicates for each experimental condition. Statistical analysis was performed to pick spots matching across all images. The significant differentially expressed protein spots (P≤0.05) with 1.5-fold or more increased or decreased intensity between resting cysts and vegetative cells were selected and subjected to further identification by MALDI-TOF MS/MS.

### Mass Spectrometry Analysis

Single protein spots from the 2-D gels were excised from a representative resting cyst gel stained with silver and submitted for tryptic digestion [10]. The gel pieces were destained with a solution of 15 mM potassium ferricyanide and 50 mM sodium thiosulfate (1∶1) and air-dried. Each sample was then digested for 16 h at 37°C with 3 µL sequencing grade trypsin (20 ng/µL). The digested samples were transferred into new centrifuge tubes and then put into 100 µL of extraction buffer (5% TFA, 67% acetonitrile) and sonicated for 15 min. Combined the enzymolysis solution and freeze dried, and then the dried proteins were dissolved in 5 µL 0.1% TFA. 1 µL of peptide mixture was spotted onto the MALDI ground steel target plate, and 1 µL of α-cyano-4-hydroxycinnamic acid saturated solution containing 50% ACN and 0.1% TFA was added into each sample. The spots were air-dried on the target plate and MS measurements were carried on a 4800 Plus MALDI TOF/TOF Analyzer (AB SCIEX, America). The laser source was Nd: YAG laser with 355 nm wavelength. The acceleration voltage was 2 kV. Data collection was obtained in the positive-ion mode and automatic acquisition data mode. The following search parameters were used: (a) Databank was NCBInr; (b) Etrieval of species were All Entries; (c) Trypsin was the specific enzyme; (d) Permit maximum miss cut site was 1; (e) Fixed modification was Carbamidomethyl, Carbamido methylation of cysteine was set as fixed modification, oxidation of methionine was as variable modification; (f) Variable modification: Oxidation(M), Acetyl (N-term); (g) MS tolerance was set as 120 ppm; (h) MS/MS tolerance was 0.4 Da. Both parent and fragment spectra were pooled to generate MS/MS spectrum of particular peptide.

### FLUTAX Fluorescent Labeling Method for Revealing Microtubular Cytoskeleton

As Yun et al [11] described with slight modification: a small amount of the resting cysts or the vegetative cells were drawn and added on a clean glass slide. The excess water was removed. Then defined amount of saponin (0.05%) was dropwised to permeate into the resting cysts or the vegetative cells for 30 s. After being washed one time with PHEM, the resting cysts or the vegetative cells were fixed in 4% paraformaldehyde for 1 min and washed again with PHEM. Subsequently, the resting cysts or the vegetative cells were treated with 0.5%Triton-X100 for 4 min and washed with PHEM. Finally, the resting cysts or the vegetative cells were stained (in dark) with 1 µmol/L FLUTAX (Invitrogen) for 8 min, and rinsed with 0.01 mol/L PBS (pH 7.2) for three times. The stained resting cysts or the vegetative cells were examined and taken photos by Olympus BX51 fluorescence microscope (excitation wavelength = 492 nm, emission wavelength = 520 nm).

### Data Analysis

The proteome maps of resting cyst and vegetative cell were compared by using PDQuest 8.0 software. The obtained MS data were analyzed, searched and identified by GPS 3.6 (Applied Biosystems) and Mascot 2.1 software (http://www. matrixscience.com/). Namely, the obtained data of peptide mass fingerprinting and MS/MS spectrum were comprehensively analyzed with GPS 3.6 and Mascot 2.1 software. The matching related proteins were searched in NCBI and Uniprot databank. A GPS Explorer protein confidence index ≥95% was used for further manual validation.

## Results

### 2-D Electrophoresis Analysis

**Figure 1:**
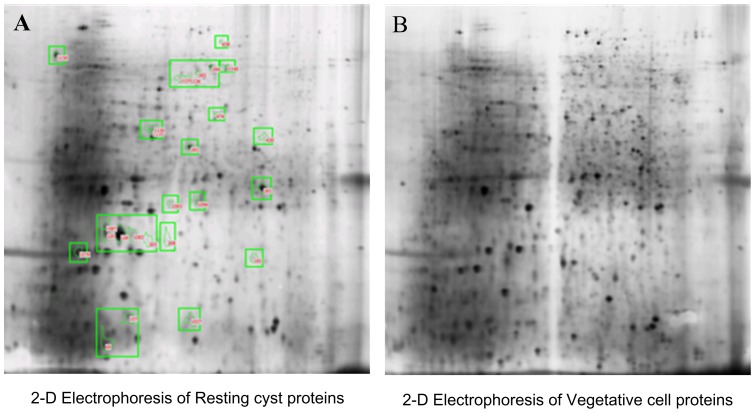
Two-dimensional electrophoresis of resting cyst proteins and vegetative cell proteins. Fig.1A and Fig.1B represent 2-D electrophoresis protein spots of resting cysts and vegetative cells of *Euplotes encysticus*, respectively. The green circles in Fig.1A showed differential expression proteins in the resting cysts compared with the vegetative cells.

Two-dimensional electrophoresis maps of the total proteins of resting cysts and vegetative cells were shown in Fig. 1. Protein profiles of resting cysts and vegetative cells were studied by comparative proteomics analysis. The six two-dimensional gels (test gels in triplicate and control gels in triplicate) were scanned by Image Scanner.

**Figure 2:**
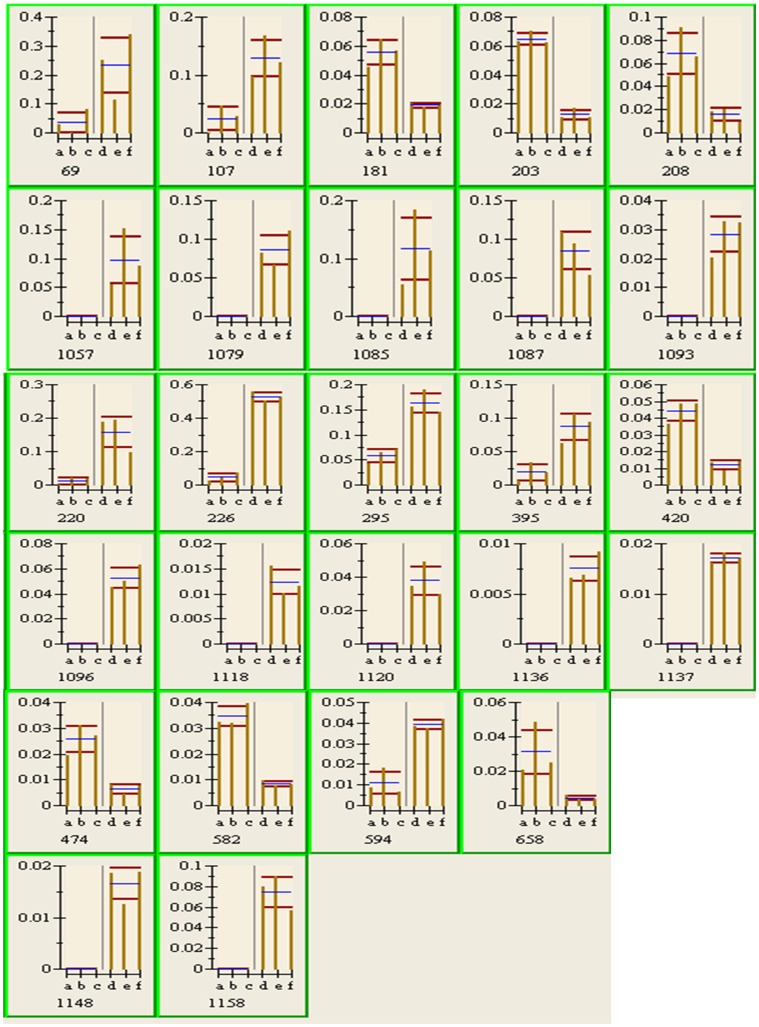
Histograms analysis of each identified protein spot. The bottom number represented the protein spot number. The blue line represented mean value in the group. The yellow vertical line represented the original value. The red line represented range of mean ± standard deviation. Letter a, b, c represented 3 repeating values in vegetative cells and letter d, e, f represented 3 repeating values in resting cysts. The y axis represented protein gray value (relative abundance of the proteins).

1266 protein spots were found and numbered through PDQuest 8.0 software analysis. These spots were statistics analyzed to identify the protein spot of differential expression. The evaluating standard is: Ratio ≥2, p value ≤0.05. 26 differential protein spots in the resting cysts were screened according to this standard (Table 1). Fig.1A showed the differential protein spots in the representative resting cyst group. The histograms analysis could more intuitively illustrate the differential spots and the details were shown in Fig. 2.

**Table 1:** Class analysis report.

Match ID (spot name)	Max (difference value)	Match Count	value in Vegetative cell	value in Resting cyst	P value
69	2.07559	2	−2.07559	2.07559	0.0451903
107	2.22897	2	−2.22897	2.22897	0.0146115
181	2.36497	2	2.36497	−2.36497	0.00318235
203	3.92345	2	3.92345	−3.92345	1.03E-04
208	2.47099	2	2.47099	−2.47099	0.0142603
220	5.16025	2	−5.16025	5.16025	0.0101538
226	7.67243	2	−7.67243	7.67243	2.29E-05
295	2.03675	2	−2.03675	2.03675	0.00279494
395	2.2655	2	−2.2655	2.2655	0.0116266
420	2.67445	2	2.67445	−2.67445	0.00191995
474	2.55973	2	2.55973	−2.55973	0.00584602
582	3.28328	2	3.28328	−3.28328	5.83E-04
594	2.28226	2	−2.28226	2.28226	0.00194621
658	3.49032	2	3.49032	−3.49032	0.0362267
1057	1.00E+06	1	1.00E+06	1.00E+06	0.026032
1079	1.00E+06	1	1.00E+06	1.00E+06	0.00236628
1085	1.00E+06	1	1.00E+06	1.00E+06	0.0343078
1087	1.00E+06	1	1.00E+06	1.00E+06	0.00737498
1093	1.00E+06	1	1.00E+06	1.00E+06	0.00228177
1096	1.00E+06	1	1.00E+06	1.00E+06	6.21E-04
1118	1.00E+06	1	1.00E+06	1.00E+06	0.00151532
1120	1.00E+06	1	1.00E+06	1.00E+06	0.00276141
1136	1.00E+06	1	1.00E+06	1.00E+06	8.78E-04
1137	1.00E+06	1	1.00E+06	1.00E+06	8.13E-06
1148	1.00E+06	1	1.00E+06	1.00E+06	0.00131403
1158	1.00E+06	1	1.00E+06	1.00E+06	0.00160574

*Among 26 differential protein spots, match count is 2 indicating the proteins expressed in both the resting cyst and vegetative cell with differential expression quantities. However, match count is 1 indicating that the proteins only expressed in the resting cyst. Positive value in the resting cyst indicated the protein was up-regulated in the resting cyst compared with the protein expression in the vegetative cell. Namely, the protein abundance in the resting cyst was higher than that in the vegetative cell. Positive value in the vegetative cell indicated the protein expression was up-regulated in the vegetative cell compared with the protein expression in the resting cyst. Conversely, negative value in the resting cyst indicated the protein expression was down-regulated in the resting cyst compared with the protein expression in the vegetative cell. Negative value in the vegetative cell indicated the protein expression was down-regulated in the vegetative cell compared with the protein expression in the resting cyst.

Combining all the above-mentioned data, we found that 26 differential protein spots in the resting cysts included 14 differential expression proteins and 12 specific expression proteins. Among the 14 differential proteins, 7 proteins were up-regulated and their up-regulated digits were 2.07559, 2.22897, 5.16025, 7.67243, 2.03675, 2.2655, 2.28226, respectively. Multiple of the up-regulated digits were in the range of 2–8. Meanwhile, the remaining 7 proteins were down-regulated and the down-regulated digits were −2.36497, −3.92345, −2.47099, −2.67445, −2.55973, −3.28328, −3.49032, respectively. Multiple of the down-regulated digits were in the range of 2–4. Compared with these 14 differential proteins, we were more interested in the 12 specific proteins of resting cyst group, which were more worth studying. Therefore, all of the 12 specific proteins and 10 out of the14 differential proteins were selected to be identified by mass spectrometry.

### MALDI-TOF MS Analysis

After searching and inquiring against NCBI database by Mascot software, detailed information of the 12 specific protein spots in resting cysts and the 10 differential protein spots were obtained and shown in Table 2, Table3, respectively.

**Table 2:** The MALDI-TOF MS analysis of 12 specific protein spots in resting cysts.

protein spots	Protein name	Accession No.	Protein MW	Protein PI	Matches	Coverage	Protein Score
1087	type II cytoskeletal 1	gi|160961491	65621	8.15	4	8%	137
1137	No significant hits	—	—	—	—	—	—
1093	Calpain-like protein	gi|403348238	78275	5.03	5	9%	46
1079	No significant hits	—	—	—	—	—	—
1120	ribosomal protein-serine acetyltransferase	gi|471232818	19803	9.1	4	35%	36
1085	Hypothetical protein TTHERM	gi|118353103	116822	5.08	23	22%	86
1148	Keratin	gi|14872730	66230	7.62	3	7%	221
1136	Nop16 domain containing protein	gi|403346091	26782	9.68	4	28%	45
1118	Hypothetical protein OXYTRI	gi|403377452	18060	5.79	7	24%	39
1057	protein arginine n-methyltransferase	gi|82594756	45074	5.83	7	24%	71
1096	hypothetical protein IMG5	gi|471233404	13110	8.86	3	26%	20
1158	trimethyllysine hydroxylase, epsilon	gi|291411954	50018	8.51	9	11%	46

**Table 3:** The MALDI-TOF MS analysis of 10 differential protein spots.

Protein spots	Protein name	Accession No.	Protein MW	Protein PI	Matches	Protein Score
69↑	Lysozyme C	gi|126608	16741.2	9.37	4	79
107↑	No significant hits	—	—	—	—	—
181↓	keratinocyte growth Factor	gi|60617190	19172.4	8.81	7	111
203↓	No significant hits	—	—	—	—	—
208↓	No significant hits	—	—	—	—	—
295↑	lysozyme homolog AT-2	gi|539969	3162.5	4.55	2	195
395↑	Formate acetyltransferase	gi|169840896	5921.3	9.86	4	49
420↓	hypothetical protein ACD	gi|406985142	23018.7	6.19	12	96
474↓	alpha S1 casein, partial	gi|159793193	18727.7	5.23	5	144
658↓	cold-shock protein	gi|401399368	13443.4	7.88	7	68

* “↑” indicates the protein expression is up-regulated in the resting cysts compared with the vegetative cells. “↓” indicates the protein expression is down-regulated in the resting cysts compared with the vegetative cells.

Generally, the criteria of a credible protein identification are protein score C.I. % >95% and protein score >50–55. Due to lack of whole proteins sequence of *Euplotes encysticus* and limitation of protozoa protein databases, the identified proteins whose protein scores ≥45 might consider to be successfully identified. As a result, we found 7 credible proteins among 12 identified specific proteins and 6 out of the 7 credible proteins were well defined, which were type II cytoskeletal 1 (No. 1087), keratin (No. 1148), Nop16 domain containing protein (No. 1136), protein arginine n-methyltransferase (No. 1057), epsilon-trimethyllysine hydroxylase (No. 1158) and calpain-like protein (No. 1093), respectively. 1 protein with undefined function is hypothetical protein TTHERM (No. 1085). The remaining proteins do not match with known function proteins, which is mainly because of scarce proteins information and uncompleted protein databank of *Euplotes encysticus*. These proteins with unknown functions were still looking forward to further identification.

Of 10 differential proteins, 7 protein dots were identified as credible proteins. Among them, 6 proteins with well defined functions were Lysozyme C (No. 69), keratinocyte growth factor (No. 181), lysozyme homolog AT-2 (No. 295), formate acetyltransferase (No. 395), alpha S1 casein (No. 474) and cold-shock protein (No. 658), respedtively. 1 protein with undefined function is hypothetical protein ACD (No. 420). 3 remaining proteins do not match with known function proteins. We speculated that some of these proteins with unknown functions might be novel proteins, which might play significant roles in the process of encystment.

### FLUTAX Fluorescent Labeling Method for Revealing Microtubular Cytoskeleton

**Figure 3:**
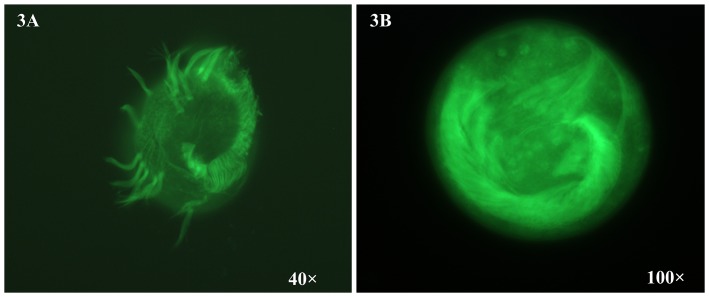
FLUTAX fluorescent labeling method for revealing microtubular cytoskeleton. Fig.3A shows the ciliature microtubules and ciliature base-associated microtubules of vegetative cells. Fig.3B shows the ciliature microtubules and ciliature base-associated microtubules of resting cysts.

Fluorescence taxtoid (FLUTAX) is a specific fluorescent dye for staining cystoskeleton. The ciliature microtubular organelles in the ventral cortex of *Euplotes encysticus* were visualized by the direct FLUTAX. The organelles consist of the adoral zone of membranelles (AZM), undulating membranes (UM), various cirrus and the ciliature base-associated microtubules. Fig.3A showed the ciliature microtubules and ciliature base-associated microtubules of vegetative cells. Fig.3B showed the ciliature microtubules and ciliature base-associated microtubules of resting cysts. Compared with the vegetative cells, ciliature microtubules and ciliature base-associated microtubules of the resting cysts rapidly changed. The ciliary shafts in the various ciliature of the cyst were partially or completely absorbed. In the resting cysts, AZM, UM and various cirrus in original venter all fell into deep of the cytoplasm and each other converge. This partially indicated that microtubular proteins and cytoskeleton proteins in the cysts sharply changed compared with the vegetative cells. This result and above identified results of cytoskeleton proteins mutually confirmed cytoskeleton-associated proteins changed in the resting cysts. These results suggested from one side that there were some differentially expressed proteins between the resting cysts and vegetative cells.

**Figure 4:**
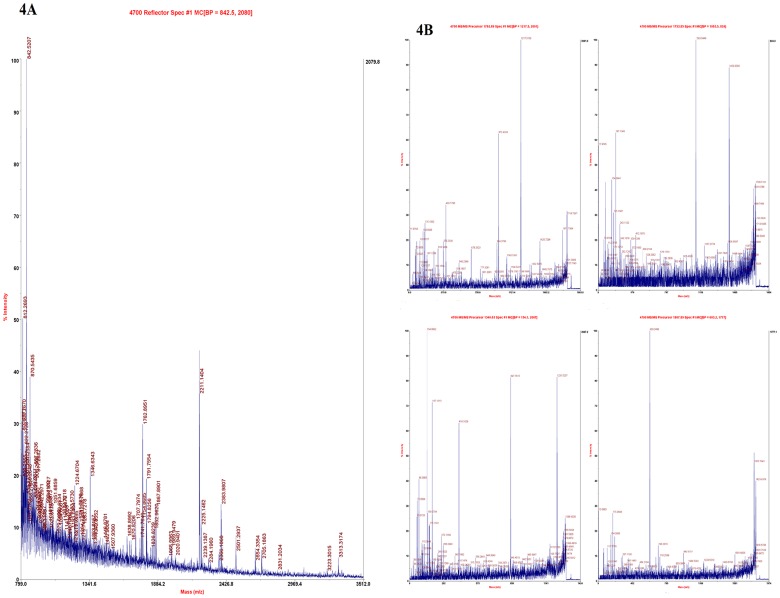
Mass spectra of the protein arginine n-methyltransferase (1057). Fig.4A represents peptide mass fingerprinting of the protein arginine n-methyltransferase. Fig.4B represents MS/MS spectra of the protein arginine n-methyltransferase.

In supplementary material, we provided peptide mass fingerprinting and MS/MS spectra for each of the identified specific protein spots in the resting cysts (Fig.S1-Fig.S11). We only took protein arginine n-methyltransferase as a representative, the peptide mass fingerprinting and MS/MS spectra of this protein were shown in this article (Fig.4). These spectra provided us with more detailed information of analysis and identification of specific proteins in the resting cysts.

## Discussion

The process of encystment in ciliates involves overall reconstruction at the cellular level. This process might be controlled or regulated by unknown intracellular signaling pathway including proteins differential expression.

### Analysis of the Specific Proteins of Resting Cyst

Ciliates encystment is clearly a RNA and protein synthesis-dependent process [12]. So the resting cyst has its specific proteins. A total of 12 specific proteins including 6 well defined proteins were obtained in our study. 6 specific proteins were type II cytoskeletal 1, keratin, Nop16 domain containing protein, protein arginine n-methyltransferase, epsilon-trimethyllysine hydroxylase and calpain-like proteins.

Among these specific proteins, keratin is a cysteine-rich or glycine-rich structural protein and has significant mechanical properties because of its hard fibrous structure. Keratin may regulate the activity of kinases, such as PKC and SRC, via binding to integrin beta-1 (ITB1) and the receptor of activated protein kinase C. Keratins are major contributors of some cells mechanical properties [13]. Type II cytoskeletal 1 is also a keratin. We inferred that keratins could strengthen the mechanical properties of cyst wall and be involved in stability of the cyst wall. Therefore, the keratins improve the ability of survival of the cyst. It is worth mentioning that keratin was found in our identified experiment of cyst wall proteins(unpublished data), which showed that keratin comprised the cytoskeletal component of the cyst wall. In addition, Gutierrez JC et al [12] pointed out that proteins with high content of glycine had been found in cyst wall of some ciliates such as *C. sleinii* and *Paraurostyla sp*. Among those which were glycine-rich, keratin (a cytoplasmic intermediate filament in vertebrate cells) should be mentioned. Namely, Gutierrez JC et al [12] suggested that keratin might be present in resting cysts of some ciliates. It needs more experiments to further confirm that keratin exists in resting cysts of some ciliates, which probably lead to argue.

Nucleolar protein is known as Nop. The stress-sensing function of the nucleolus including Nop16 is one of its most important newly identified roles. More and more evidence indicated that the nucleolus played a role as a coordinator of cellular stress responses [14].The nucleolus senses stress is a central hub for coordinating the stress response. Classification of the molecular functions of Nop shows that only ∼30% have a function clearly related to the production of ribosome subunits. The other functions including biogenesis of multiple RNPs, cell-cycle control, apoptosis, resist viral infection, DNA replication and repair, are consistent with a role for the nucleolus in providing a link between ribosome subunit biosynthesis, cell-cycle progression and stress signaling [15].So Nop probably not only played significant roles in the stress response but also involved in some peculiar proteins expression associated with the encystment.

It is well known that posttranslational modification of proteins is related to structural and functional diversity and is a mechanism frequently used to regulate cellular signaling events. Arginine dimethylation is a common modification of specific proteins. Arginine methylation by protein *N*-arginine methyltransferases (PRMTs) is an important posttranslational modification in the regulation of protein signaling. PRMTs catalyze the sequential transfer of methyl groups from S-adeonsyl- L-methionine to the guanidinonitrogens of arginine residues in proteins, the effect of which include regulation of signal transduction, transcription regulation, RNA transport, chromatin remodeling and DNA repair. So far, nine PRMTs (PRMT 1∼9) have been isolated [16]. Each of the various PRMTs may play distinct cellular roles. For instance, PRMT 1 may be required for specific developmental processes or stress responses. I-Smads, Smad6 and Smad7 are methylated by PRMT1. PRMT2 interacts with the methyl acceptor heterogeneous nuclear ribonucleoprotein E1BAP5 through its Src homology 3 domain. Methylation of STAT1 and STAT3 by PRMT enzymes may regulate their signaling pathways. PRMT6 activity affects gene regulation primarily by modifying protein-nucleic acid interactions. PRMT6 localizes exclusively to the cell nucleus, exhibits automethylation, and methylates *in vitro* glycine and arginine-rich (GAR) sequences in proteins. Methylation of polymerase β by PRMT6 was shown to increase its repair activity of damaged DNA, implicating PRMT6 as a regulator of base excision repair. PRMT6 is a negative regulator of cellular as well as viral transcriptional activation [17]. Symmetric and asymmetric protein methylation by PRMT enzymes has been shown to regulate the transduction of signals to the nucleus, transcript ion regulation through nuclear receptors and RNA transport between the nucleus and cytoplasm, which suggests that modulating this signaling event may have far- reaching impact [18]. Therefore, PRMTs regulated signal transduction, transcription, RNA transport, chromatin remodeling and DNA repair through posttranslational modification of proteins during the encystment. Multifunction of PRMTs suggested PRMTs might significantly contribute to the process of encysment.

ε-*N*-trimethyllysine hydroxylase (TMLH) was also a specific protein in the resting cysts. It is known that TMLH is the first enzyme in the biosynthetic pathway of L-carnitine and catalyzes the formation of β-hydroxy-*N*-ε-trimethyllysine from ε-*N*-trimethyllysine. Carnitine is a vital compound, which plays an indispensable role in the transport of activated fatty acids across the inner mitochondrial membrane into the matrix, where β-oxidation takes place. Furthermore, carnitine is involved in the transfer of the products of peroxisomal β-oxidation, including acetyl-CoA. TMLH is predominantly localized in mitochondria. The submitochondrial localization of TMLH will have implications for the substrate flow and regulation of the carnitine biosynthesis [19]. Therefore, we inferred that TMLH had important influences on the energy metabolism of the encystment.

Many experimental data have showed that calpains are a ubiquitous family of calcium-dependent cysteine proteases and play important roles in a wide range of cell stress response, cell regulatory and differentiation processes through restricting enzymolysis of various enzymes in cell and cytoskeleton protein system. In many protozoa organisms, atypical calpains have been discovered and most of these novel calpain-like proteins are non-enzymatic homologues of typical calpains [20]. Recently, calpain-like proteins could be acted as microtubule-interacting proteins [21]. Because many morphological changes were coordinated with the alteration of cytoskeleton protein system during the encystment, calpain-like proteins likely played vital roles in the system of cytoskeleton protein reorganization in encystation process.

In short, these specific proteins are believed to play important roles in the encystment.

### Analysis of the Differential Proteins of Resting Cyst

The proteomic analysis performed in this study revealed that 14 protein spots of the resting cysts showed significant differences in protein abundance compared with the vegetative cells. Among these differential proteins, 10 proteins were identified by mass spectrometry and Bioinformatics. Of the 10 identified proteins, 6 proteins were with defined functions and discussion were focused on these proteins including Lysozyme C, lysozyme homolog AT-2, alpha S1 casein, formate acetyltransferase, keratinocyte growth factor and Cold shock proteins.

Lysozyme including lysozyme C widely distributes among eukaryotes and prokaryotes, it is an important component of the innate immune response system. It mainly hydrolyzes the β-1, 4-glycosidic linkages between *N*-acetylmuramic acid and *N*-acetylglucosamine in peptidoglycan leading to bacterial lysis [22]. Lysozyme homolog AT-2 is also a lysozyme and has lysis bacteria function. Alpha S1 casein also shows antibacterial activity and is an antibacterial peptide [23]. Due to *Euplotes encysticus* feeding on bacteria, the activities of bacteriolytic enzymes in the resting cysts changed to adapt to dormancy state during encystment.

Formate acetyltransferase (also known as pyruvate formatelyase, PFL) was an important differential protein in the resting cysts. It catalyzes the reversible conversion of pyruvate and CoA into acetyl-CoA and formate [24], [25]. Its activity enhancement probably means that acetyl-CoA increases. Acetyl-CoA is a central material in energy metabolism process and its increase helps to satisfy energy metabolism demand of the resting cysts. Apparently the resting cysts having a strikingly reduced metabolism were not ‘sleeping’ and they still had low energy metabolism level.

We speculated that *Euplotes encysticus*, just like other animal before dormancy, transformed the food bacterium into stored energy as much as possible. So activity of the lysosome increased due to stress during the encystment. Similarly, formate acetyltransferese was up-regulated during encystment in order to transform stored energy as well. These two enzymes should decrease in the mature resting cysts.

Keratinocyte growth factor (KGF) completely prevented the increase in permeability caused by the peroxide. KGF can reduce oxidative damage, indicating that KGF could increase the levels of enzymes which detoxify reactive oxygen species. For example, peroxiredoxin VI, an enzyme that detoxifies hydrogen peroxide and organic peroxides, was identified as a KGF target gene [26], [27]. However, KGF was down-regulated in the resting cysts, which might indicated that KGF probably could not encounter oxidative stress.

Cold shock proteins (CSPs) were also differential proteins in the resting cysts. CSPs can work under both normal and cold-shock conditions, mainly inducing by a rapid temperature downshift in order to adapt to cold stress. Whereas, they also exist in normal conditions to regulate other biological functions. These general functions involve in RNA chaperoning and transcriptional antitermination. CSPs appear to serve various cellular functions in the context of stress response, but they are not antifreeze proteins [28]–[30]. For single-cell organisms, CSPs play critical roles during the cold adaptation process. However, we found that the abundance of CSPs declined in the resting cysts. We speculated that CSPs expressed in the early stage of encystment and were successively degraded in the subsequent process, which leaded to the CSPs declined in the resting cysts.

The peculiar and differential proteins mentioned above covered a wide range of molecular functions, such as gene regulation, RNA regulation, proteins degradation and stress resistance, material transport and cytoskeleton organization. Therefore, they were essential for cell morphological and physiological changes during encystment.

## Conclusions

*Euplotes encysticus* adapts to environmental stress through transforming into resting cyst. One mystery underlying the encystment is the proteins expression changes. The most striking result of this study was to identify a large number of the specific proteins and the differential proteins related to the encystment. These protein functions were discussed and possibly involved in RNA regulation, proteins degradation and stress resistance, material transport and cytoskeleton organization etc. Diversity of these protein functions suggested that they might played important roles in the encystment process. Notably, proteins with unknown function among the identified specific proteins were those which were usually not similar to any known protein. However, their expressions might be associated with the stress tolerance and encystment. Therefore, these proteins are likely novel proteins and may be the best candidates for sources of resisting adverse environmental stress in future studies.

The results and analysis in this study will help to unravel the molecular mechanisms underlying encystment and eukaryote dormancy.

## Supporting Information

Figure S1 **Mass spectra of spot (1079) in resting cyst.** A: Peptide mass fingerprinting of spot (1079) in resting cyst; B1-B6: MS/MS spectrum of spot (1079) in resting cyst.
(PDF)

Figure S2 **Mass spectra of spot (1085) in resting cyst.** A: Peptide mass fingerprinting of hypothetical protein TTHERM (1085) in resting cyst; B1-B12: MS/MS spectrum of hypothetical protein TTHERM (1085) in resting cyst.
(PDF)

Figure S3 **Mass spectra of spot (1087) in resting cyst.** A: Peptide mass fingerprinting of type II cytoskeletal (1087) in resting cyst; B1-B12: MS/MS spectrum of type II cytoskeletal (1087) in resting cyst.
(PDF)

Figure S4 **Mass spectra of spot (1093) in resting cyst.** A: Peptide mass fingerprinting of Calpain-like protein (1093) in resting cyst; B: MS/MS spectrum of Calpain-like protein (1093) in resting cyst.
(PDF)

Figure S5 **Mass spectra of spot (1096) in resting cyst.** A: Peptide mass fingerprinting of hypothetical protein IMG5 (1096) in resting cyst; B1-B5: MS/MS spectrum of hypothetical protein IMG5 (1096) in resting cyst.
(PDF)

Figure S6 **Mass spectra of spot (1118) in resting cyst.** A: Peptide mass fingerprinting of hypothetical protein OXYTRI (1118) in resting cyst; B: MS/MS spectrum of hypothetical protein OXYTRI (1118) in resting cyst.
(PDF)

Figure S7 **Mass spectra of spot (1120) in resting cyst.** A: Peptide mass fingerprinting of ribosomal protein-serine acetyltransferase (1120) in resting cyst; B: MS/MS spectrum of ribosomal protein-serine acetyltransferase (1120) in resting cyst.
(PDF)

Figure S8 **Mass spectra of spot (1136) in resting cyst.** A: Peptide mass fingerprinting of Nop16 domain containing protein (1136) in resting cyst; B1-B6: MS/MS spectrum of Nop16 domain containing protein (1136) in resting cyst.
(PDF)

Figure S9 **Mass spectra of spot (1137) in resting cyst.** A: Peptide mass fingerprinting of spot (1137) in resting cyst; B1-B3: MS/MS spectrum of spot (1137) in resting cyst.
(PDF)

Figure S10 **Mass spectra of spot (1148) in resting cyst.** A: Peptide mass fingerprinting of keratin (1148) in resting cyst; B1-B12: MS/MS spectrum of keratin (1148) in resting cyst.
(PDF)

Figure S11 **Mass spectra of spot (1158) in resting cyst.** A: Peptide mass fingerprinting of trimethyllysine hydroxylase (1158) in resting cyst. B: MS/MS spectrum of trimethyllysine hydroxylase (1158) in resting cyst.
(PDF)
