# Supplementary material for: Proteomic Approach to Reveal the Proteins Associated with Encystment of the Ciliate Euplotes encysticus
Source: PLoS One. 2014 May 16;9(5):e97362. doi: 10.1371/journal.pone.0097362 (PMC4023950; doi:10.1371/journal.pone.0097362)
Supplement: Figure S1 — Mass spectra of spot (1079) in resting cyst. A: Peptide mass fingerprinting of spot (1079) in resting cyst; B1-B6: MS/MS spectrum of spot (1079) in resting cyst. (PDF) [file pone.0097362.s001.pdf]

A

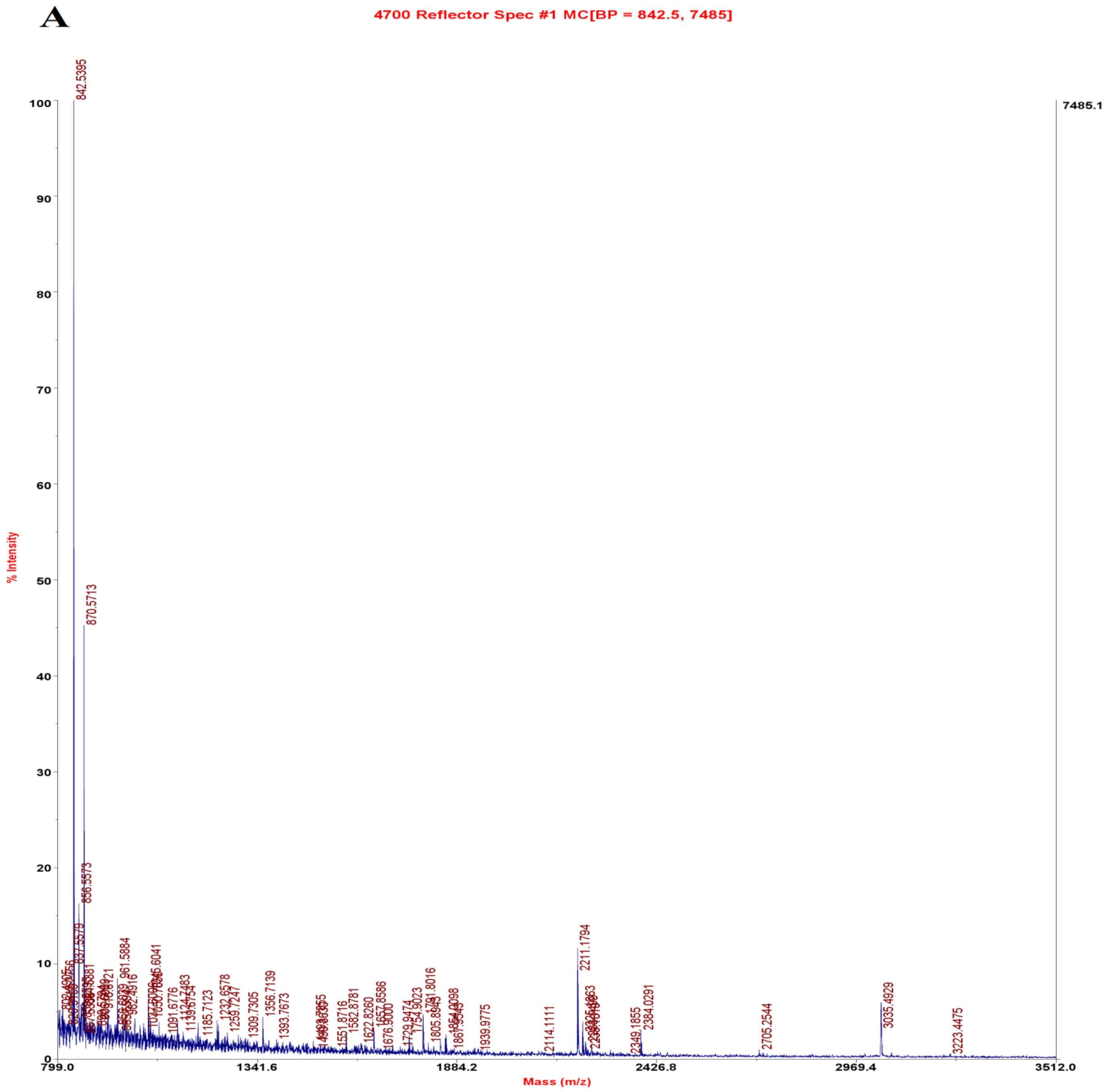
4700 Reflector Spec #1 MC[BP = 842.5, 7485]
% Intensity
Mass (m/z)
7485.1
100
90
80
70
60
50
40
30
20
10
0
799.0
1341.6
1884.2
2426.8
2969.4
3512.0
842.5395
870.5713
856.5573
961.5884
1091.6776
1185.7123
1232.6578
1259.7247
1309.7305
1356.7139
1393.7673
1551.8716
1582.8781
1622.8260
1657.8586
1676.9000
1729.9474
1754.9023
1791.8016
1805.8943
1854.0098
1939.9775
2114.1111
2211.1794
2349.1855
2384.0291
2705.2544
3035.4929
3223.4475

B1

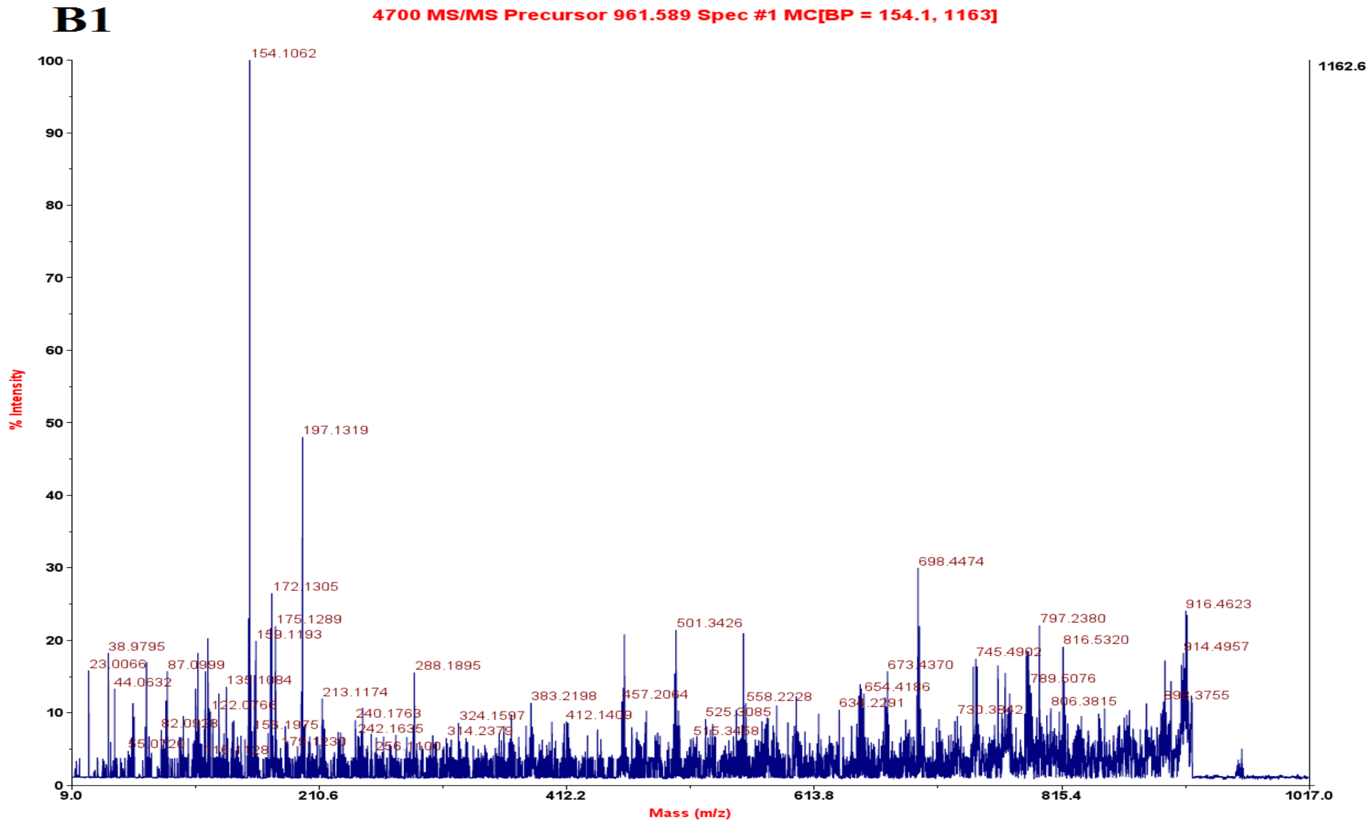
4700 MS/MS Precursor 961.589 Spec #1 MC[BP = 154.1, 1163]
% Intensity
Mass (m/z)
1162.6
154.1062
197.1319
698.4474
172.1305
175.1289
159.1193
501.3426
797.2380
916.4623
816.5320
914.4957
38.9795
23.0066
87.0999
44.0632
288.1895
745.4902
673.4370
213.1174
383.2198
457.2064
558.2228
789.5076
654.4186
806.3815
240.1763
412.1409
324.1597
525.3085
634.2291
730.3842
314.2379
515.3458
9.0
210.6
412.2
613.8
815.4
1017.0

B2

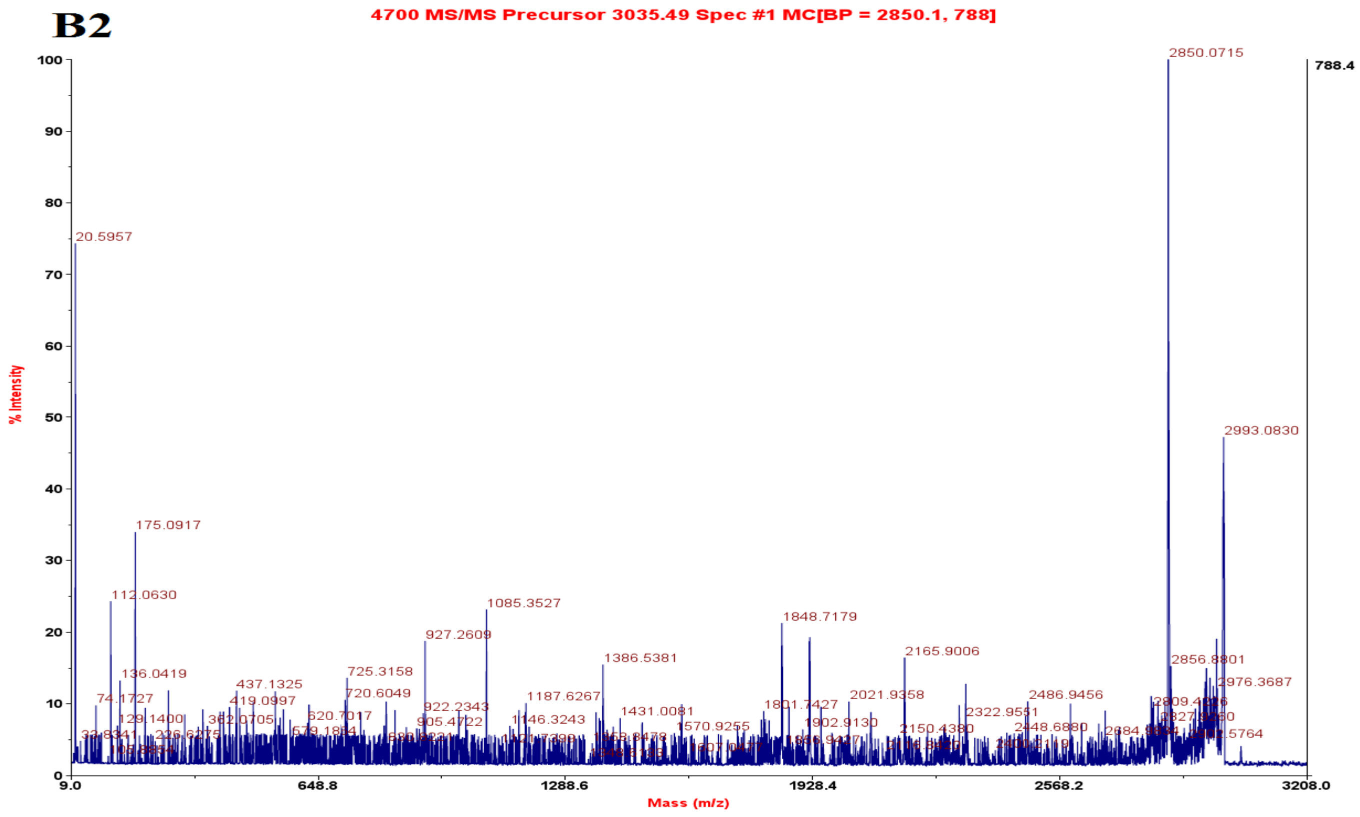
4700 MS/MS Precursor 3035.49 Spec #1 MC[BP = 2850.1, 788]
% Intensity
Mass (m/z)
788.4
2850.0715
20.5957
2993.0830
175.0917
112.0630
1085.3527
1848.7179
927.2609
2165.9006
2856.8801
1386.5381
136.0419
437.1325
725.3158
2976.3687
419.0997
720.6049
74.1727
1187.6267
2021.9358
2486.9456
1801.7427
922.2343
1431.0081
2322.9551
129.1400
520.7017
905.4722
1146.3243
1902.9130
2448.6880
1570.9255
2150.4380
9.0
648.8
1288.6
1928.4
2568.2
3208.0

B3

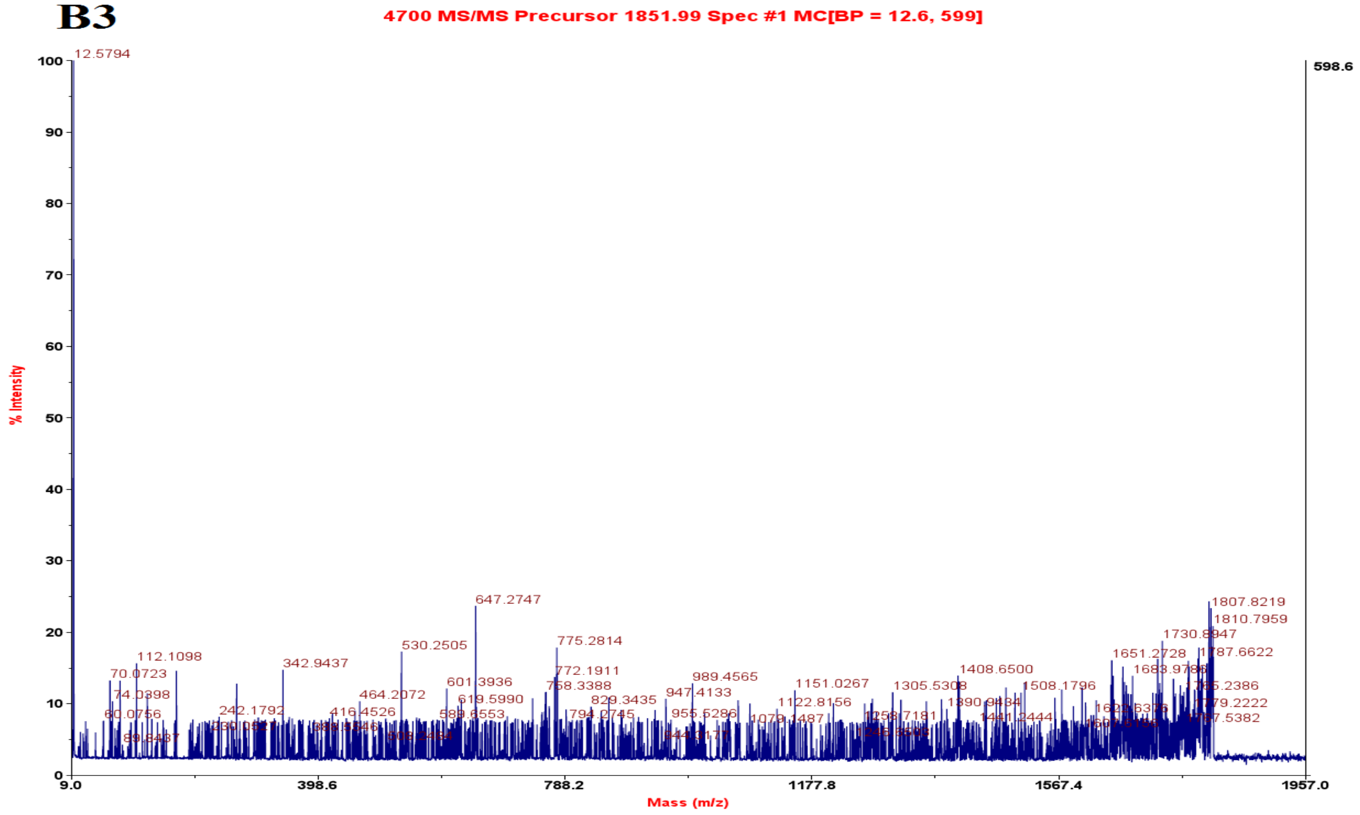
4700 MS/MS Precursor 1851.99 Spec #1 MC[BP = 12.6, 599]
% Intensity
Mass (m/z)
598.6
12.5794
647.2747
1807.8219
1810.7959
775.2814
530.2505
342.9437
112.1098
70.0723
1730.8947
1651.2728
1787.6622
1408.6500
1151.0267
1305.5308
1508.1796
989.4565
601.3936
464.2072
242.1792

B4

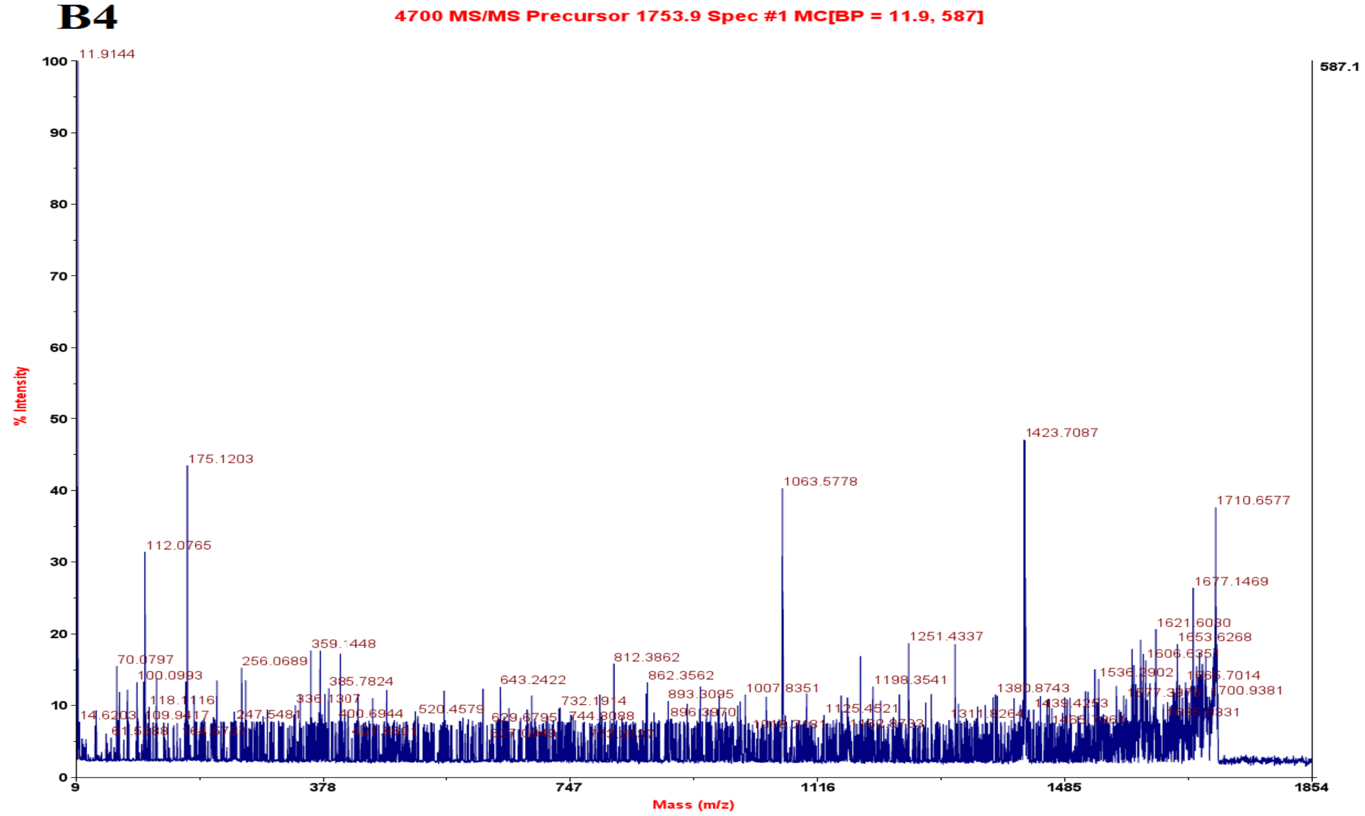
4700 MS/MS Precursor 1753.9 Spec #1 MC[BP = 11.9, 587]
% Intensity
Mass (m/z)
587.1
11.9144
1423.7087
175.1203
1063.5778
1710.6577
112.0765
1677.1469
1621.6030
1653.6268
1251.4337
1198.3541
812.3862
862.3562
643.2422
359.1448
385.7824
256.0689
70.0797
100.0993
1007.8351
1380.8743

B5

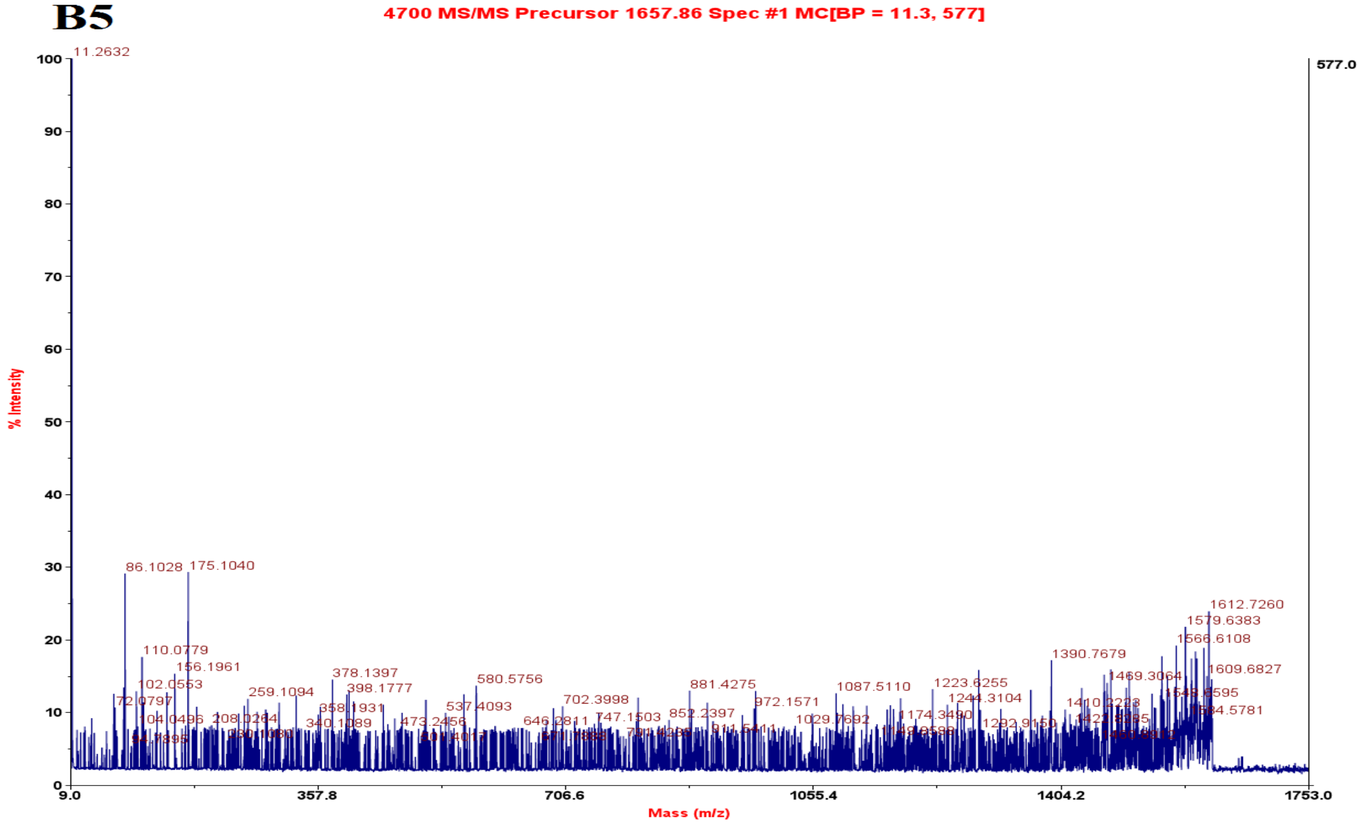

B6

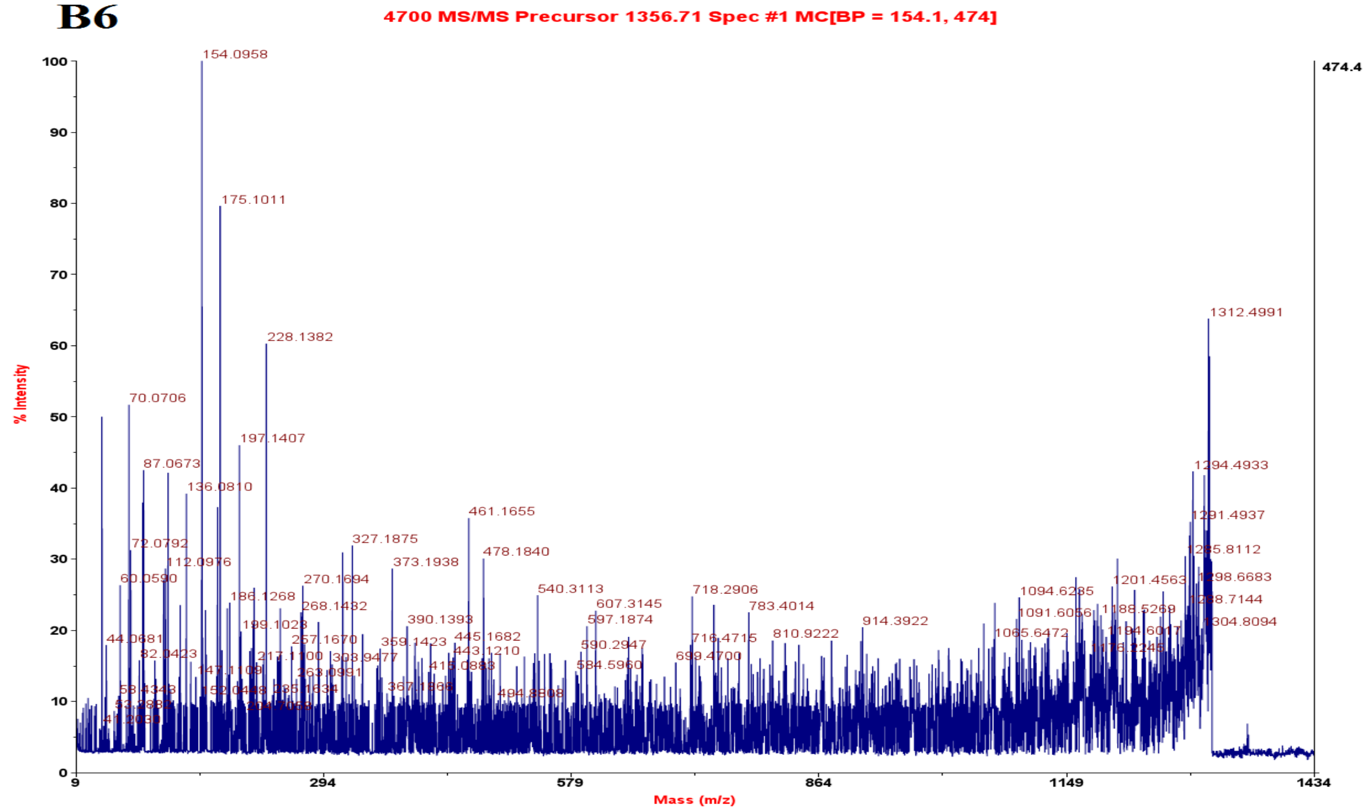

Fig. S1 Mass spectra of spot (1079) in resting cyst

A: Peptide mass fingerprinting of spot (1079) in resting cyst; B1-B6: MS/MS spectrum of spot (1079) in resting cyst.
