# Supplementary material for: Proteomic Approach to Reveal the Proteins Associated with Encystment of the Ciliate Euplotes encysticus
Source: PLoS One. 2014 May 16;9(5):e97362. doi: 10.1371/journal.pone.0097362 (PMC4023950; doi:10.1371/journal.pone.0097362)
Supplement: Figure S2 — Mass spectra of spot (1085) in resting cyst. A: Peptide mass fingerprinting of hypothetical protein TTHERM (1085) in resting cyst; B1-B12: MS/MS spectrum of hypothetical protein TTHERM (1085) in resting cyst. (PDF) [file pone.0097362.s002.pdf]

A

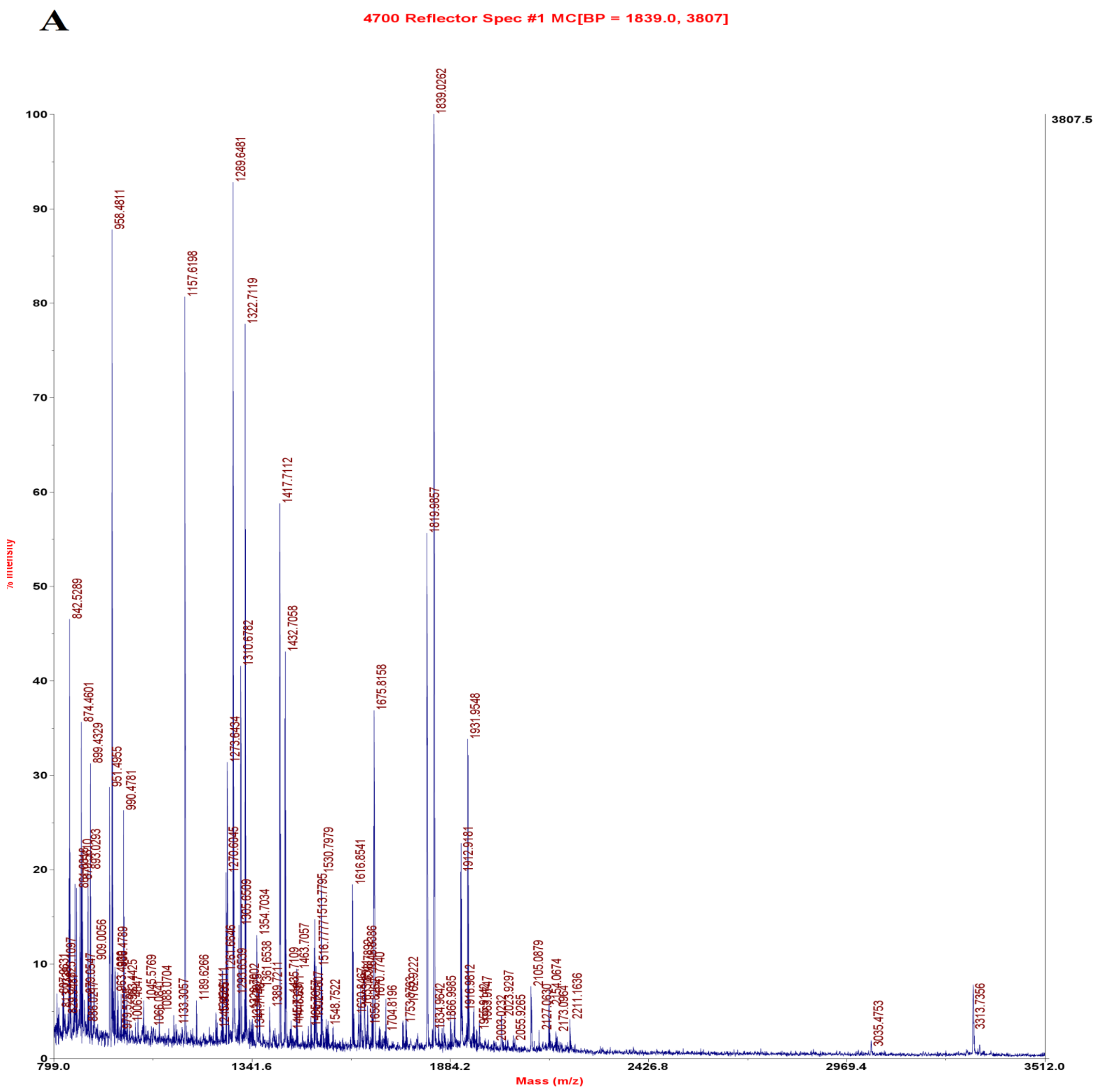
4700 Reflector Spec #1 MC[BP = 1839.0, 3807]
% Intensity
Mass (m/z)
3807.5
1839.0262
1289.6481
958.4811
1157.6198
1322.7119
1417.7112
1819.9857
842.5289
1432.7058
1310.6782
1675.8158
874.4601
1931.9548
899.4329
1273.6434
951.4955
990.4781
1270.6045
893.0293
1912.9181
1530.7979
1616.8541
1354.7034
2105.0879
3313.7356
3035.4753
2211.1636
1189.6266
1045.5769
1133.3057
1548.7522
1704.8196
799.0
1341.6
1884.2
2426.8
2969.4
3512.0

B1

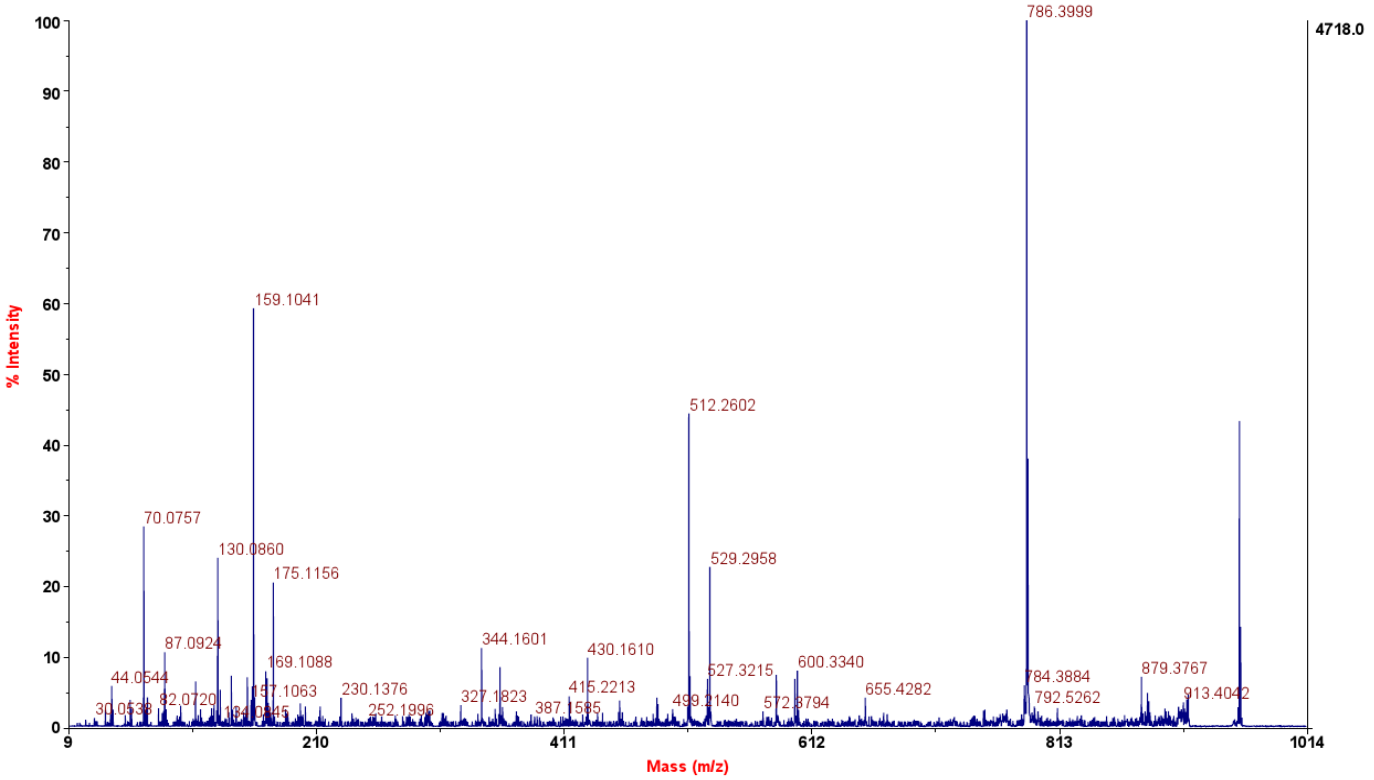
4700 MS/MS Precursor 958.481 Spec #1 MC[BP = 786.4, 4718]
% Intensity
Mass (m/z)
4718.0
786.3999
159.1041
512.2602
529.2958
70.0757
130.0860
175.1156
87.0924
169.1088
44.0544
82.0720
30.0538
157.1063
230.1376
252.1996
344.1601
327.1823
387.1585
430.1610
415.2213
499.2140
527.3215
572.3794
600.3340
655.4282
784.3884
792.5262
879.3767
913.4042

B2

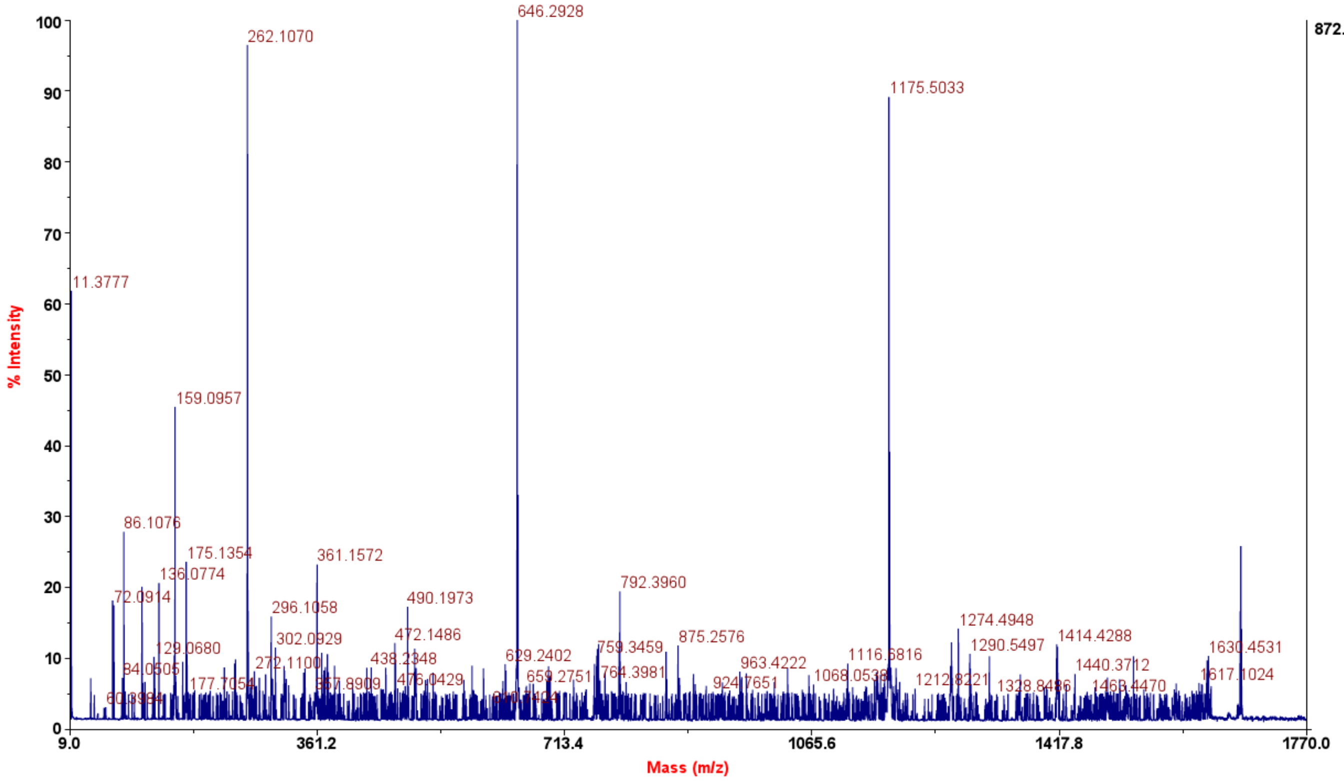
4700 MS/MS Precursor 1674.8 Spec #1 MC[BP = 646.3, 873]
% Intensity
Mass (m/z)
872.9
646.2928
262.1070
1175.5033
11.3777
159.0957
86.1076
175.1354
136.0774
72.0914
129.0680
84.0505
60.9984
177.7054
361.1572
296.1058
302.0929
272.1100
357.8909
490.1973
472.1486
438.2348
476.0429
629.2402
659.2751
792.3960
759.3459
764.3981
875.2576
963.4222
924.7651
1068.0538
1116.5816
1274.4948
1290.5497
1212.8221
1328.8486
1414.4288
1440.3712
1468.4470
1630.4531
1617.1024

B3

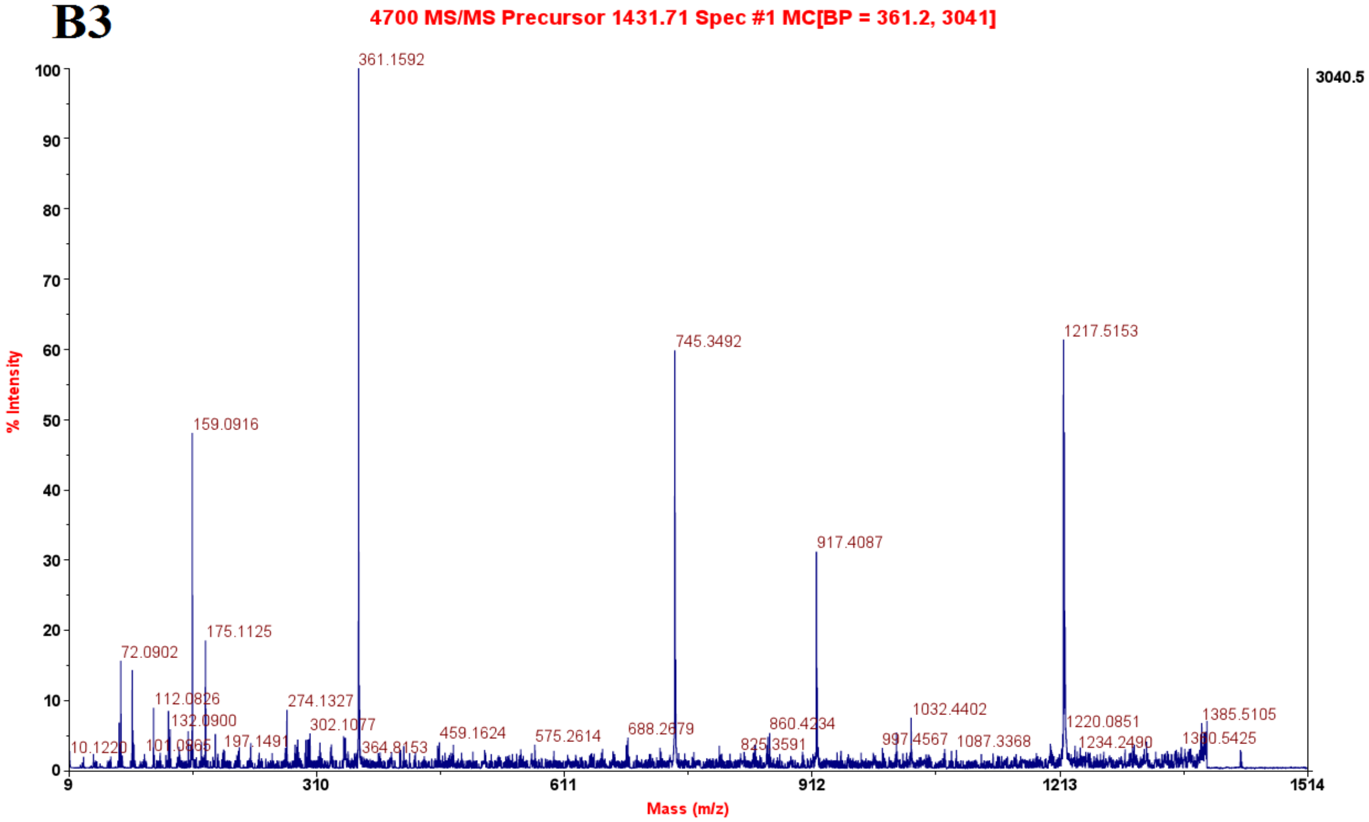

4700 MS/MS Precursor 1431.71 Spec #1 MC[BP = 361.2, 3041]
% Intensity
Mass (m/z)
3040.5
361.1592
745.3492
1217.5153
159.0916
917.4087
175.1125
72.0902
112.0826
274.1327
1032.4402
132.0900
302.1077
860.4234
1385.5105
459.1624
688.2679
1220.0851
575.2614
364.8153
1087.3368
1234.2490
197.1491

B4

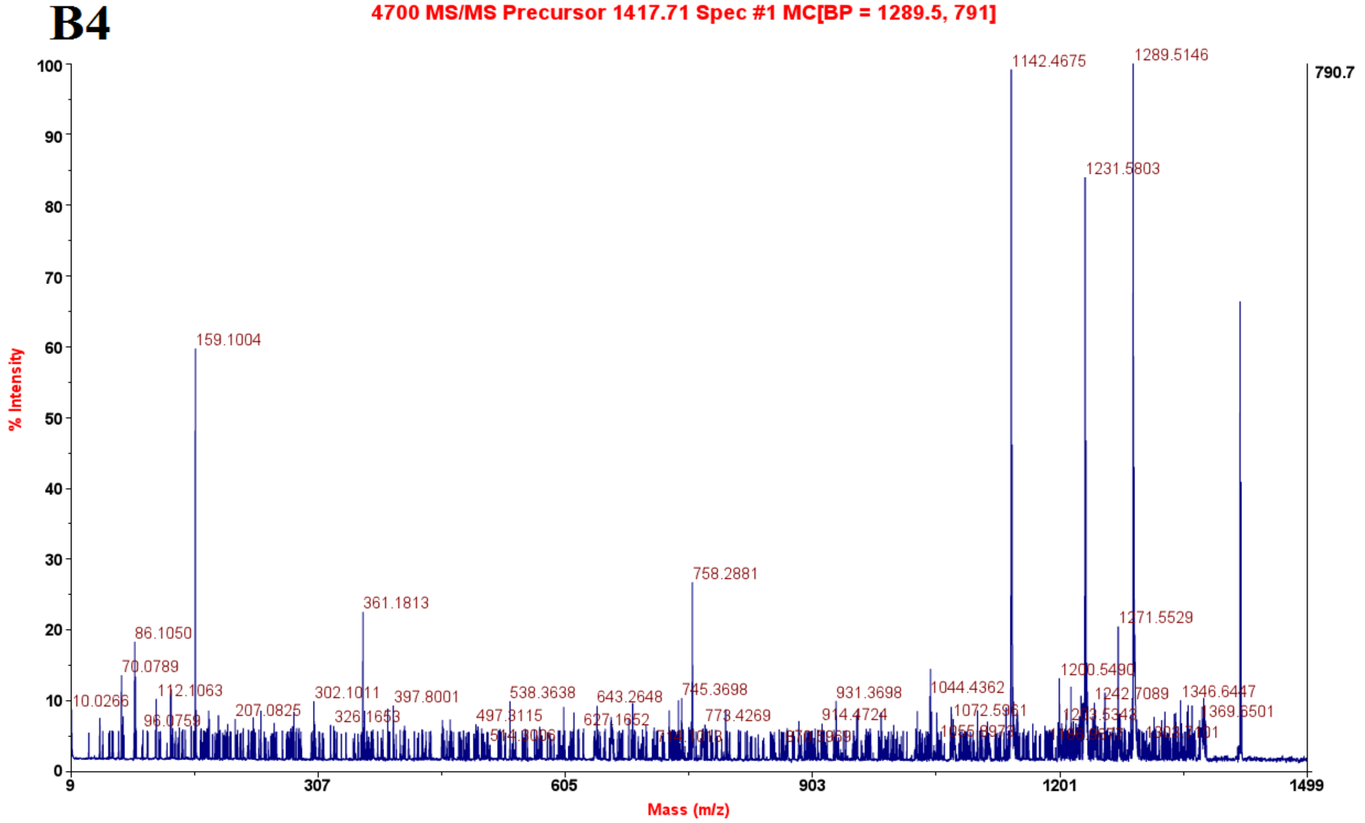

4700 MS/MS Precursor 1417.71 Spec #1 MC[BP = 1289.5, 791]
% Intensity
Mass (m/z)
790.7
1142.4675
1289.5146
1231.5803
159.1004
758.2881
361.1813
1271.5529
86.1050
70.0789
1200.5490
112.1063
10.0266
302.1011
397.8001
538.3638
643.2648
745.3698
931.3698
1044.4362
1242.7089
1346.6447
207.0825
1369.6501
497.3115
773.4269
914.4724
96.0759
627.1652

B5

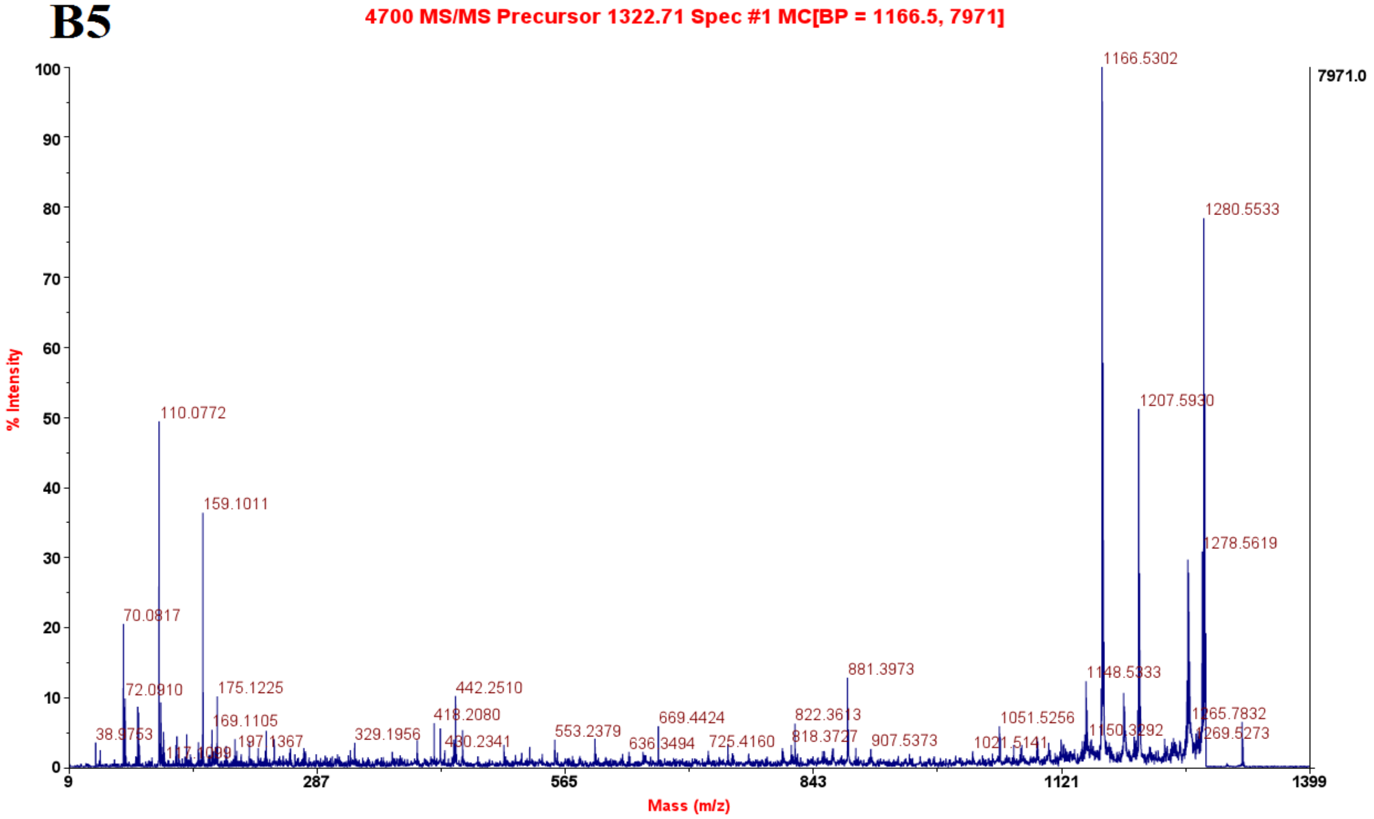
4700 MS/MS Precursor 1322.71 Spec #1 MC[BP = 1166.5, 7971]
% Intensity
Mass (m/z)
7971.0
1166.5302
1280.5533
1207.5930
1278.5619
110.0772
159.1011
70.0817
72.0910
175.1225
169.1105
38.9763
442.2510
418.2080
430.2341
329.1956
553.2379
669.4424
636.3494
725.4160
822.3613
818.3727
881.3973
907.5373
1051.5256
1021.5141
1148.5333
1150.3292
1265.7832
1269.5273

B6

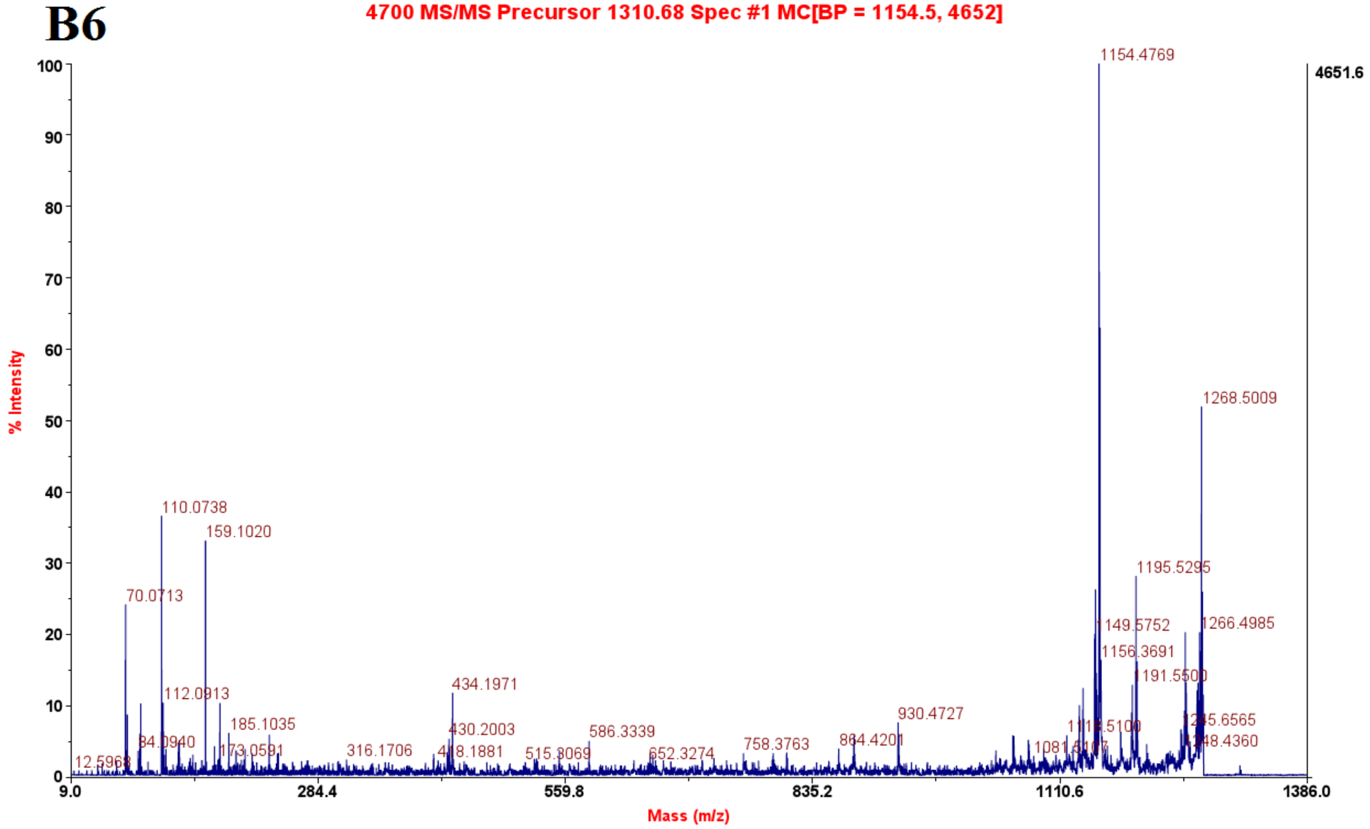
4700 MS/MS Precursor 1310.68 Spec #1 MC[BP = 1154.5, 4652]
% Intensity
Mass (m/z)
4651.6
1154.4769
1268.5009
1195.5295
1149.5752
1266.4985
1156.3691
110.0738
159.1020
70.0713
112.0913
185.1035
84.0940
173.0591
12.5968
434.1971
430.2003
418.1881
316.1706
515.3069
586.3339
652.3274
758.3763
864.4201
930.4727
1081.5107
1245.6565
1248.4360

B7

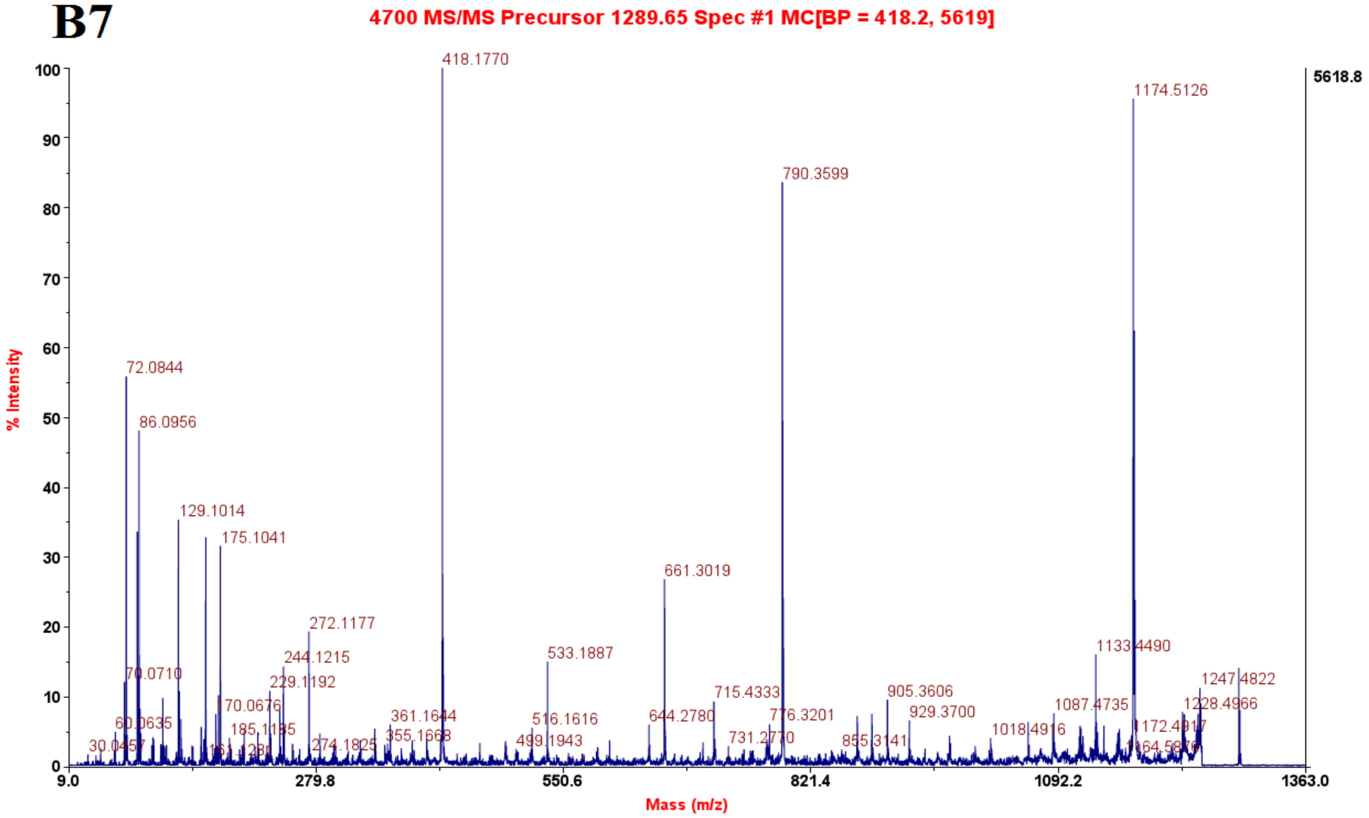
4700 MS/MS Precursor 1289.65 Spec #1 MC[BP = 418.2, 5619]
% Intensity
Mass (m/z)
5618.8
418.1770
1174.5126
790.3599
72.0844
86.0956
129.1014
175.1041
661.3019
272.1177
244.1215
533.1887
229.1192
1133.4490
1247.4822
70.0710
715.4333
905.3606
170.0676
1087.4735
1228.4966
60.0635
361.1644
516.1616
644.2780
776.3201
929.3700
185.1185
355.1668
1018.4916
1172.4917
731.2770
30.0457
274.1825
499.1943
855.3141
1164.5876
9.0
279.8
550.6
821.4
1092.2
1363.0

B8

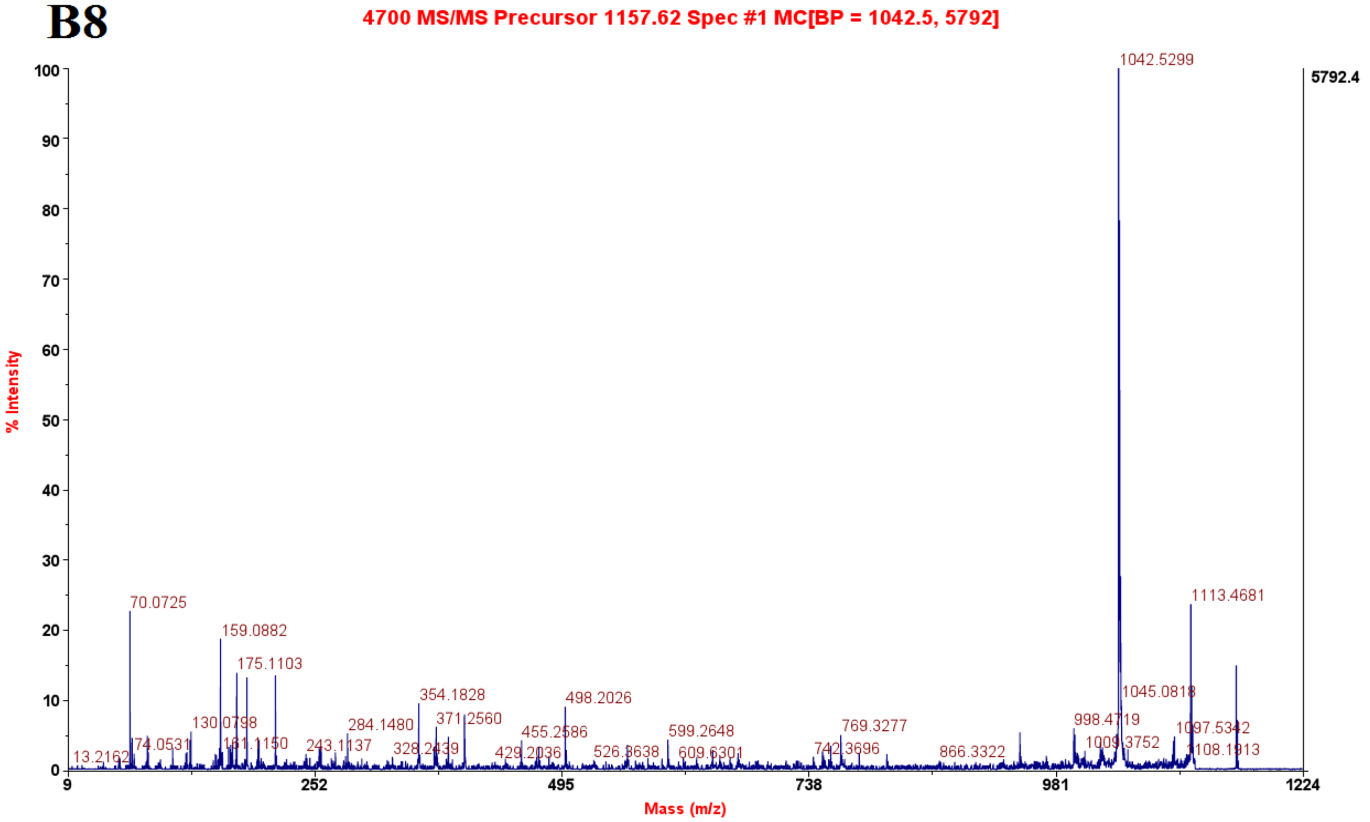
4700 MS/MS Precursor 1157.62 Spec #1 MC[BP = 1042.5, 5792]
% Intensity
Mass (m/z)
5792.4
1042.5299
1113.4681
70.0725
159.0882
175.1103
354.1828
498.2026
1045.0818
130.0798
371.2560
769.3277
998.4719
284.1480
455.2586
599.2648
1097.5342
74.0531
161.1150
243.1137
1009.3752
328.2439
742.3696
866.3322
1108.1913
13.2162
429.2036
526.3638
609.6301
9
252
495
738
981
1224

B9

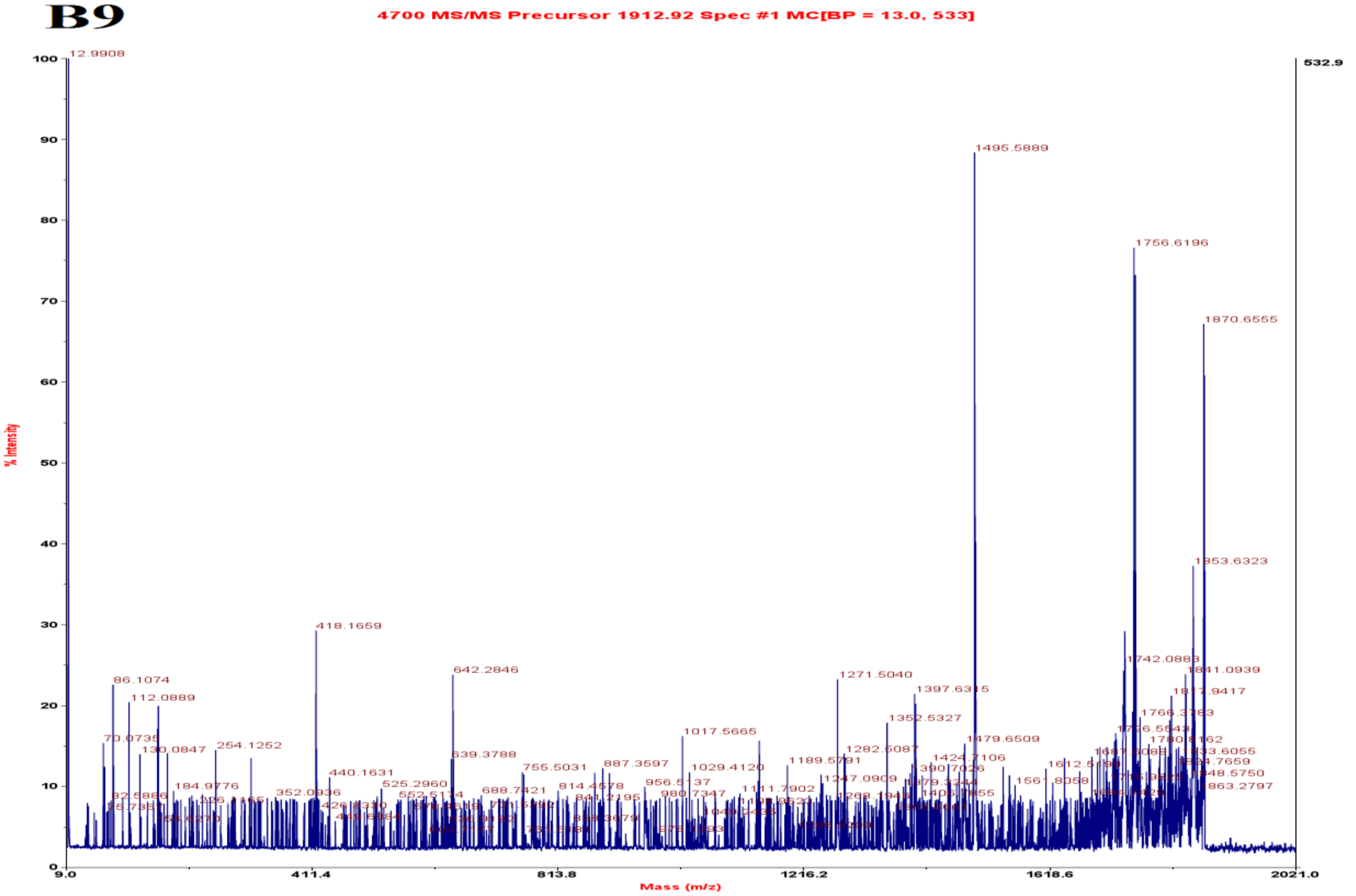
4700 MS/MS Precursor 1912.92 Spec #1 MC[BP = 13.0, 533]
% Intensity
Mass (m/z)
532.9
12.9908
1495.5889
1756.6196
1870.6555
1853.6323
418.1659
642.2846
1271.5040
1742.0883
1841.0939
86.1074
112.0889
1397.6315
1017.5665
254.1252
639.3788
1479.6509
755.5031
887.3597
440.1631
9.0
411.4
813.8
1216.2
1618.6
2021.0

B10

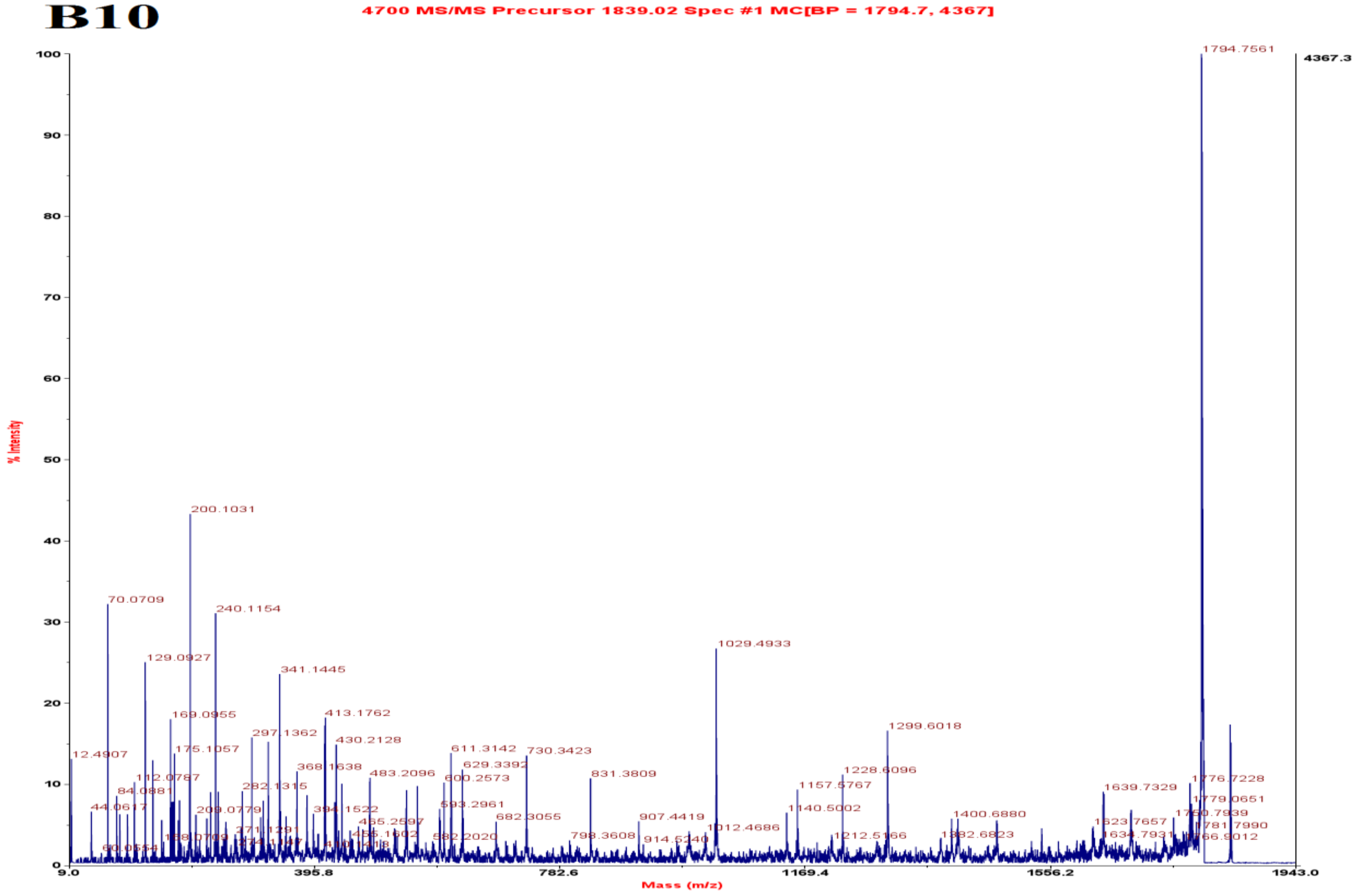
4700 MS/MS Precursor 1839.02 Spec #1 MC[BP = 1794.7, 4367]
% Intensity
Mass (m/z)
4367.3
1794.7561
200.1031
70.0709
240.1154
1029.4933
129.0927
341.1445
169.0955
413.1762
1299.6018
297.1362
430.2128
175.1057
12.4907
611.3142
730.3423
368.1638
629.3392
483.2096
1228.6096
1776.7228
831.3809
1639.7329
1157.5767
1400.6880
9.0
395.8
782.6
1169.4
1556.2
1943.0

B11

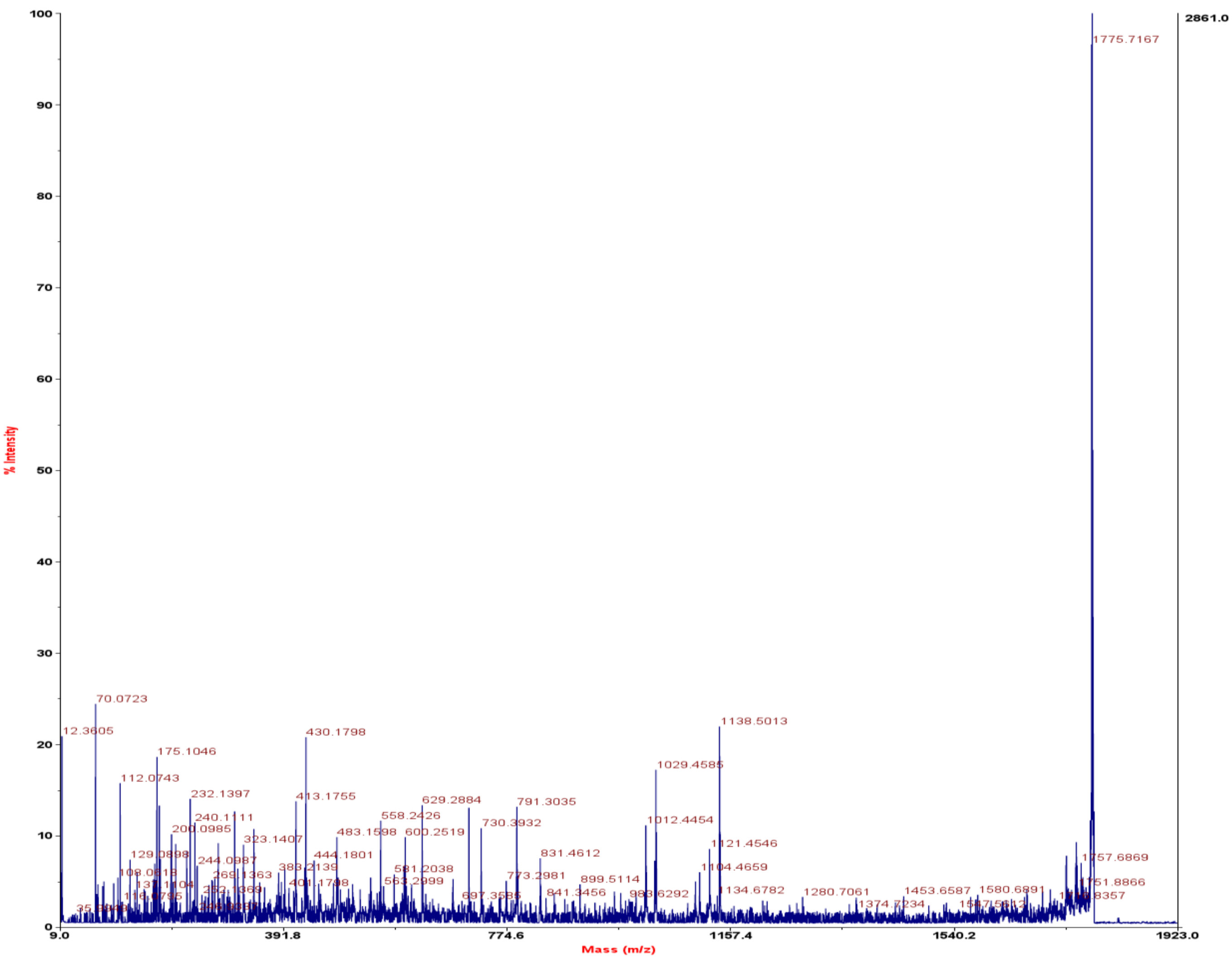
4700 MS/MS Precursor 1819.99 Spec #1 MC[BP = 1776.7, 2861]
2861.0
% Intensity
Mass (m/z)
100
90
80
70
60
50
40
30
20
10
0
9.0
391.8
774.6
1157.4
1540.2
1923.0
1775.7167
70.0723
12.3605
1138.5013
430.1798
175.1046
112.0743
1029.4585
232.1397
413.1755
629.2884
791.3035
558.2426
240.1111
730.3932
1012.4454
200.0985
483.1598
600.2519
323.1407
1121.4546
129.0898
444.1801
831.4612
244.0987
1757.6869
108.0818
383.2139
581.2038
1104.4659
269.1363
773.2981
899.5114
563.2999
401.1798
1751.8866
1134.6782
1280.7061
1453.6587
1580.6891
841.3456
697.3586
1374.7234

B12

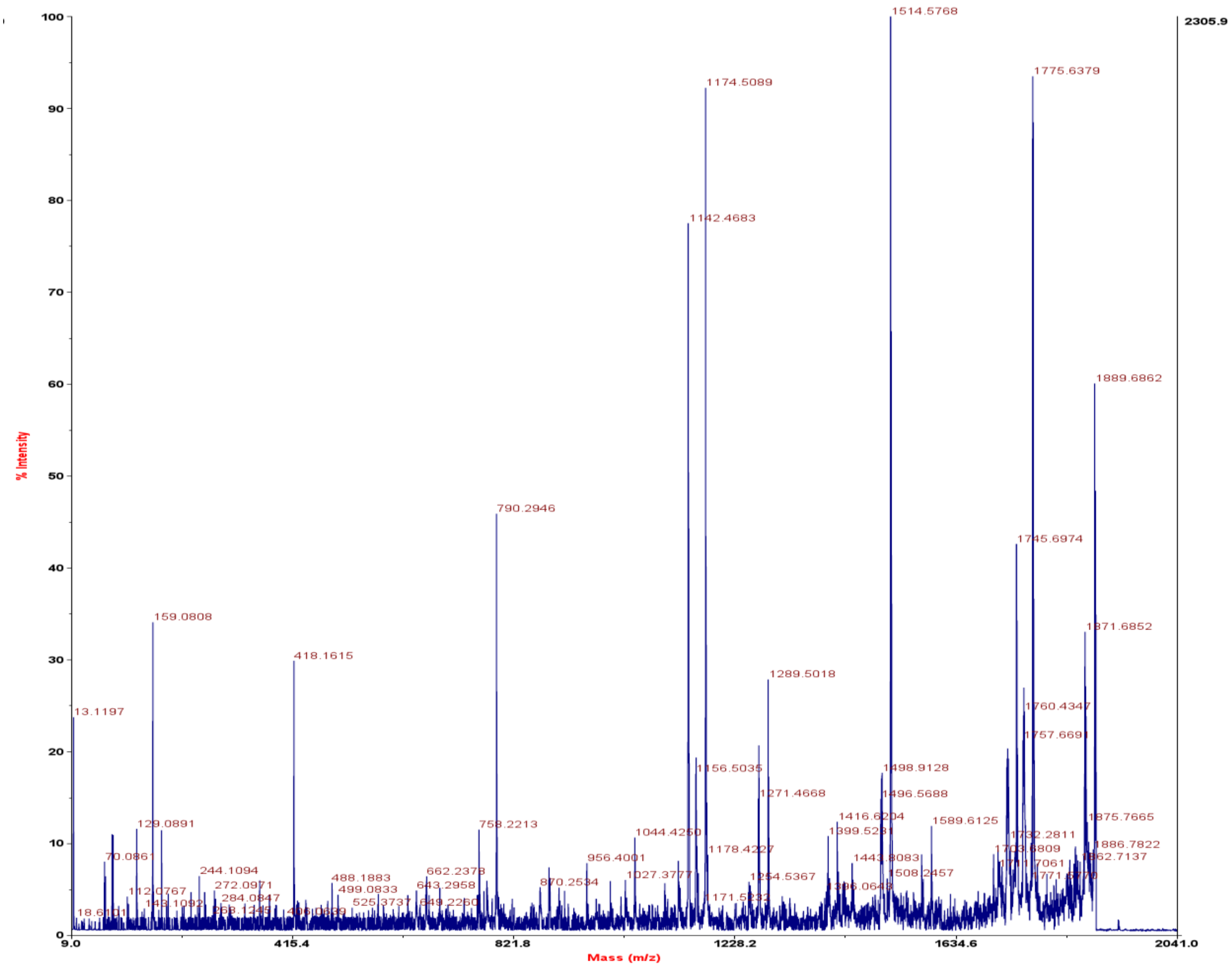

Fig. S2 Mass spectra of spot (1085) in resting cyst

A: Peptide mass fingerprinting of hypothetical protein TTHERM (1085) in resting cyst; B1-B12: MS/MS spectrum of hypothetical protein TTHERM (1085) in resting cyst.
