# Supplementary material for: Proteomic Approach to Reveal the Proteins Associated with Encystment of the Ciliate Euplotes encysticus
Source: PLoS One. 2014 May 16;9(5):e97362. doi: 10.1371/journal.pone.0097362 (PMC4023950; doi:10.1371/journal.pone.0097362)
Supplement: Figure S3 — Mass spectra of spot (1087) in resting cyst. A: Peptide mass fingerprinting of type II cytoskeletal (1087) in resting cyst; B1-B12: MS/MS spectrum of type II cytoskeletal (1087) in resting cyst. (PDF) [file pone.0097362.s003.pdf]

A

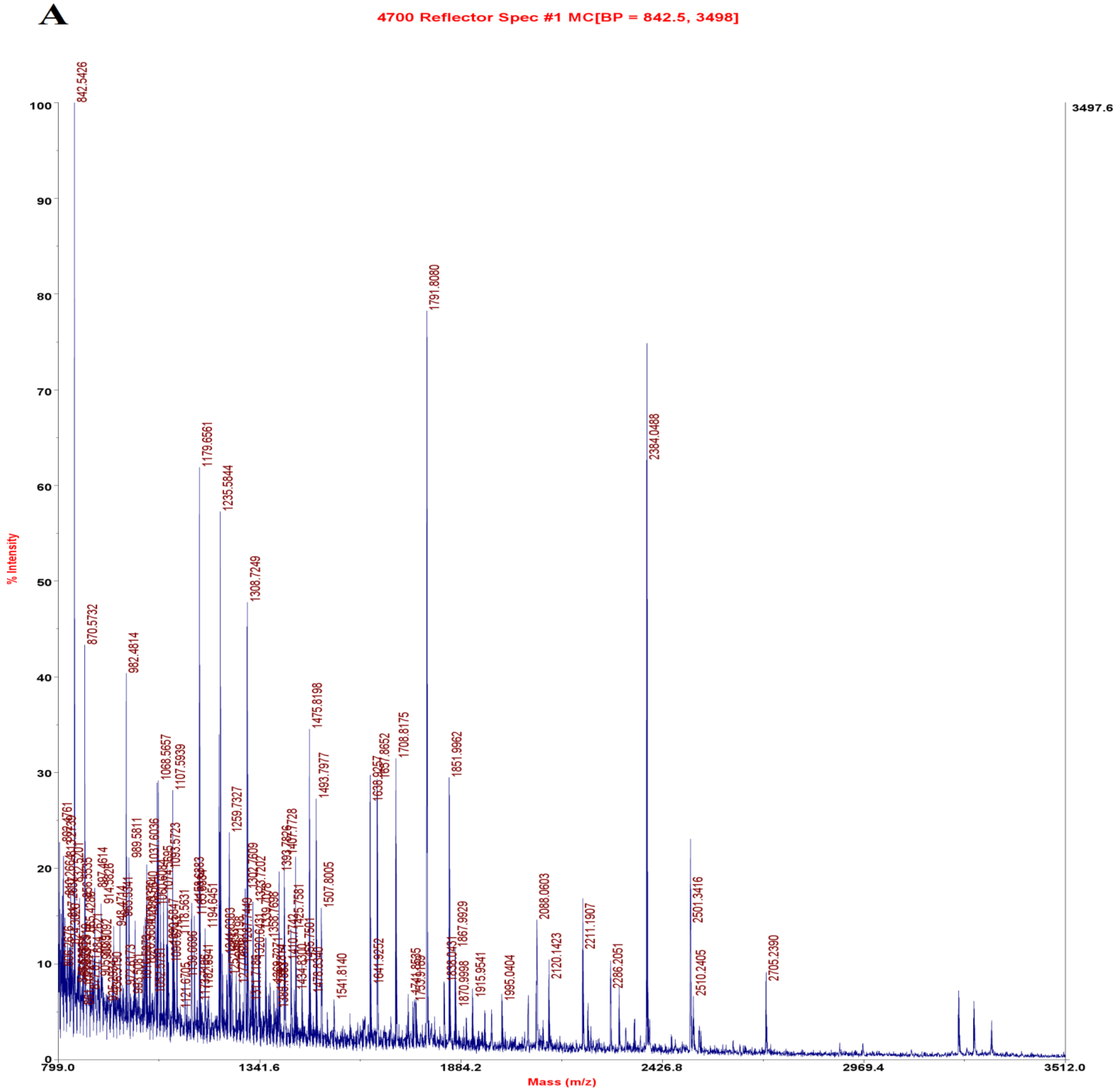
4700 Reflector Spec #1 MC[BP = 842.5, 3498]
% Intensity
Mass (m/z)
3497.6
100
90
80
70
60
50
40
30
20
10
0
799.0
1341.6
1884.2
2426.8
2969.4
3512.0
842.5426
1791.8080
2384.0488
1179.6561
1235.5844
1308.7249
870.5732
982.4814
1475.8198
1708.8175
1637.8652
1851.9962
1493.7977
1068.5657
1107.5939
1259.7327
1407.7728
1393.7826
989.5811
1037.6036
1093.5723
887.4614
914.3828
1507.8005
1425.7581
1194.6451
1867.9929
2088.0603
2211.1907
2120.1423
2286.2051
2501.3416
2510.2405
2705.2390
1995.0404
1915.9541
1870.9998
1641.9252
1541.8140
1121.6705

B1

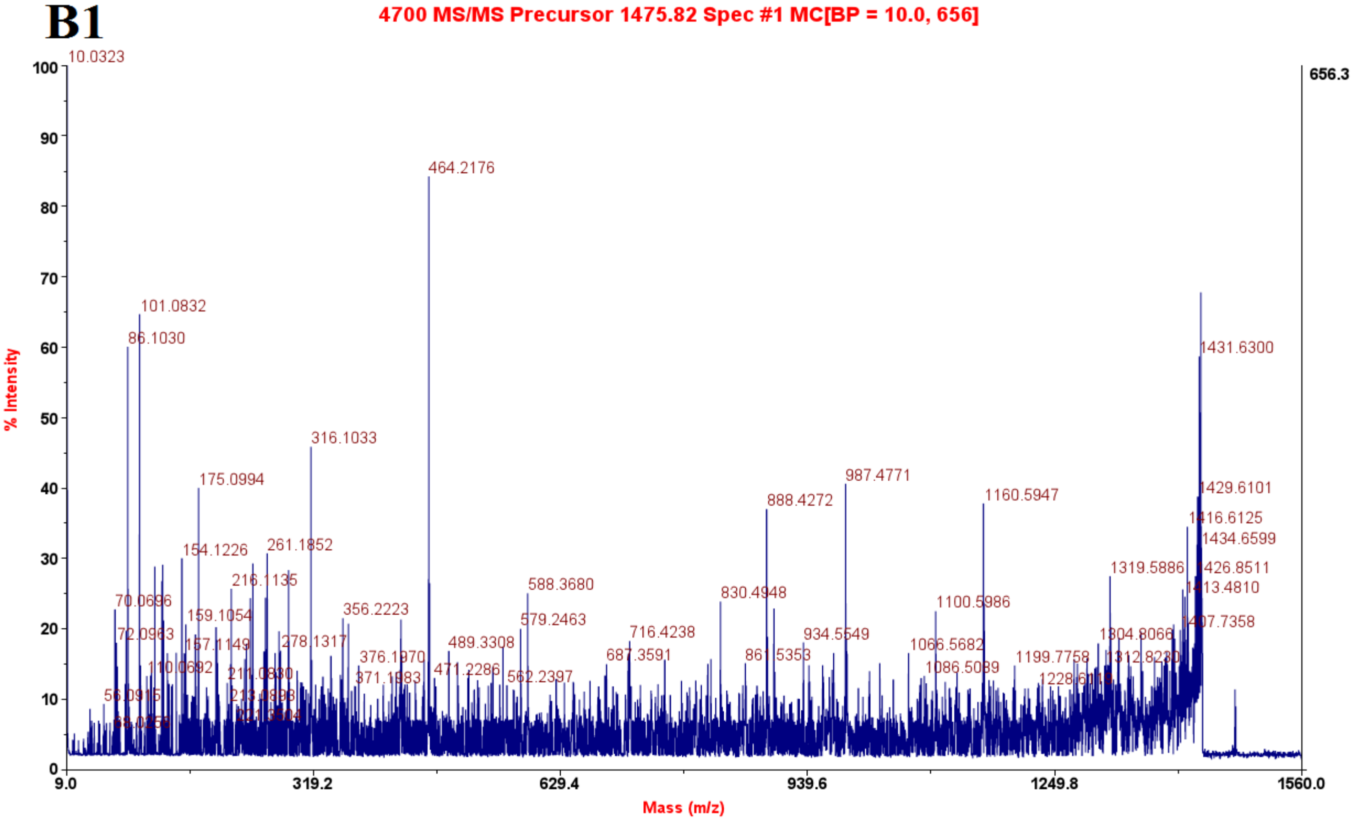
4700 MS/MS Precursor 1475.82 Spec #1 MC[BP = 10.0, 656]
% Intensity
Mass (m/z)
656.3
10.0323
464.2176
101.0832
86.1030
316.1033
175.0994
154.1226
261.1852
216.1135
70.0696
159.1054
72.0963
157.1149
278.1317
356.2223
376.1970
371.1983
489.3308
471.2286
588.3680
579.2463
562.2397
716.4238
687.3591
830.4948
888.4272
861.5353
934.5549
987.4771
1100.5986
1066.5682
1086.5089
1160.5947
1199.7758
1319.5886
1304.8066
1431.6300
1429.6101
1416.6125
1434.6599
1426.8511
1413.4810
1407.7358
9.0
319.2
629.4
939.6
1249.8
1560.0

B2

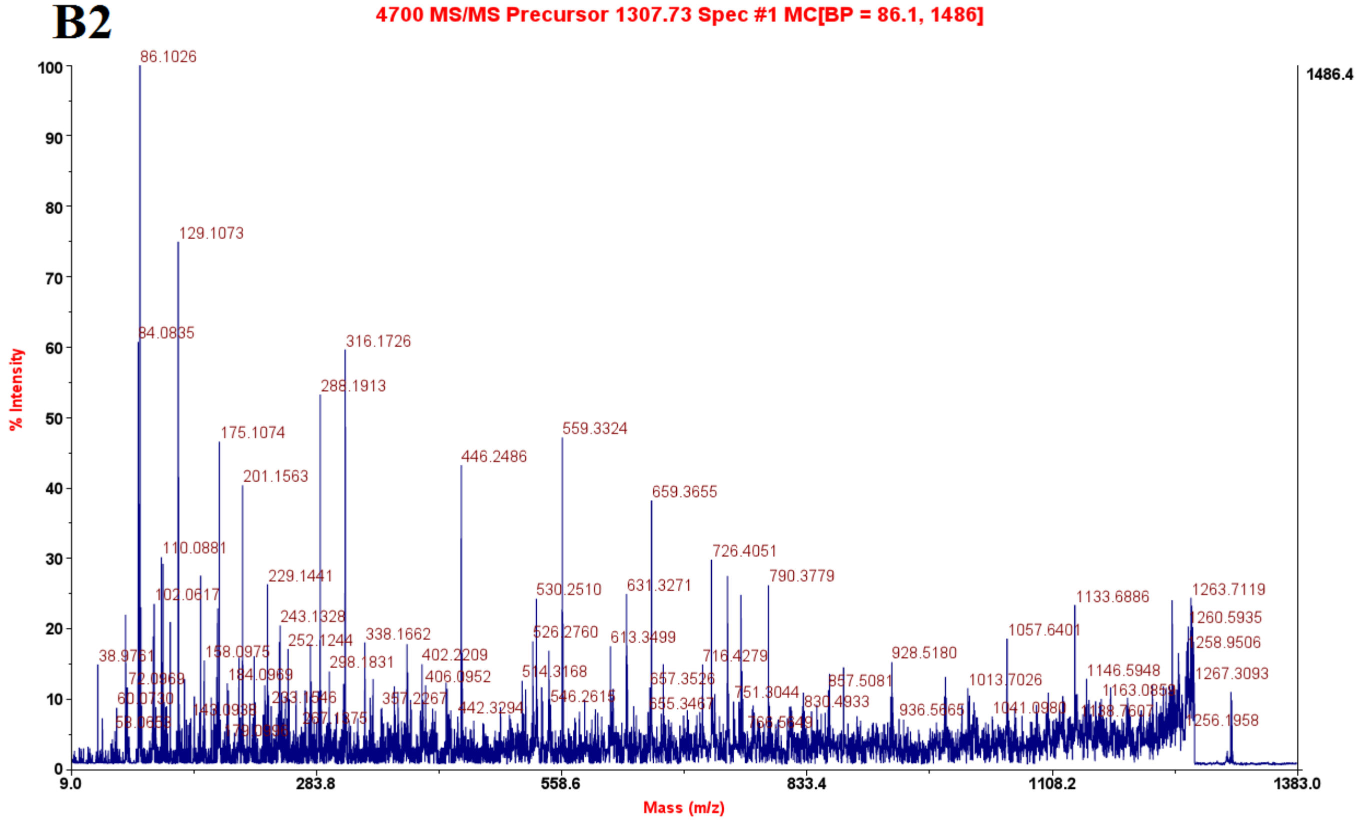
4700 MS/MS Precursor 1307.73 Spec #1 MC[BP = 86.1, 1486]
% Intensity
Mass (m/z)
1486.4
86.1026
129.1073
84.0835
316.1726
288.1913
175.1074
201.1563
110.0881
102.0617
229.1441
243.1328
252.1244
338.1662
298.1831
38.9761
72.0969
158.0975
184.0969
402.2209
406.0952
357.2267
442.3294
446.2486
559.3324
530.2510
526.2760
514.3168
546.2615
631.3271
613.3499
659.3655
657.3526
655.3467
726.4051
716.4279
790.3779
751.3044
857.5081
830.4933
928.5180
936.5665
1013.7026
1041.0980
1057.6401
1133.6886
1146.5948
1163.0858
1263.7119
1260.5935
1258.9506
1267.3093
1256.1958
9.0
283.8
558.6
833.4
1108.2
1383.0

B3

4700 MS/MS Precursor 1259.73 Spec #1 MC[BP = 1193.5, 1738]

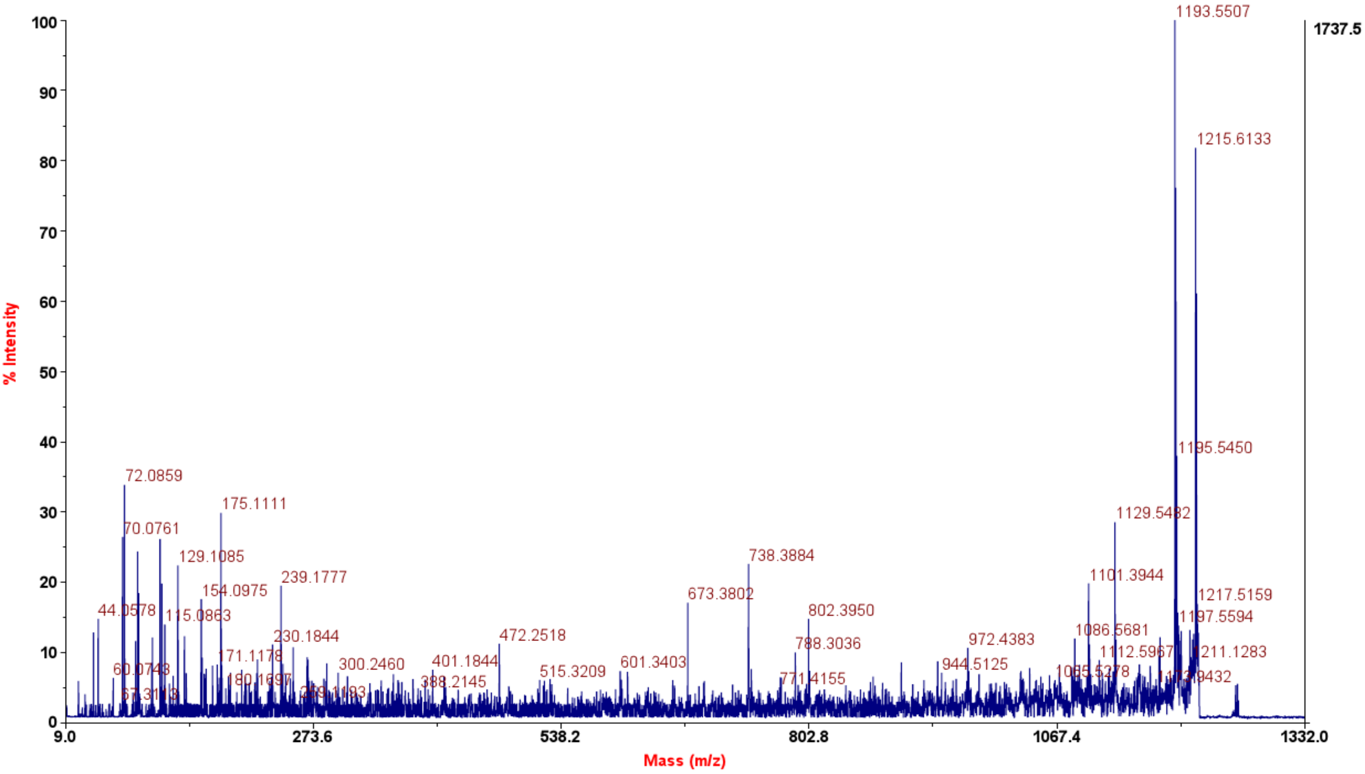

B4

4700 MS/MS Precursor 1235.58 Spec #1 MC[BP = 175.1, 2317]

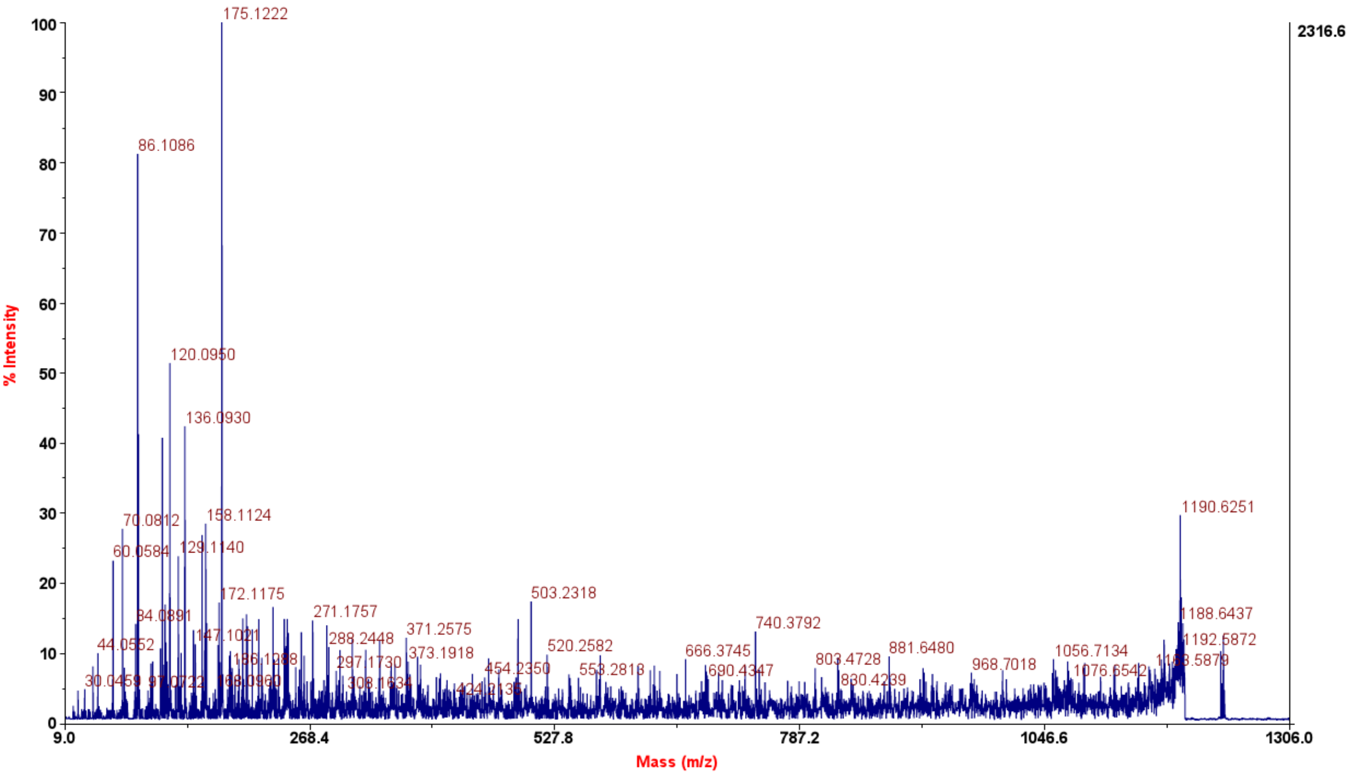

B5

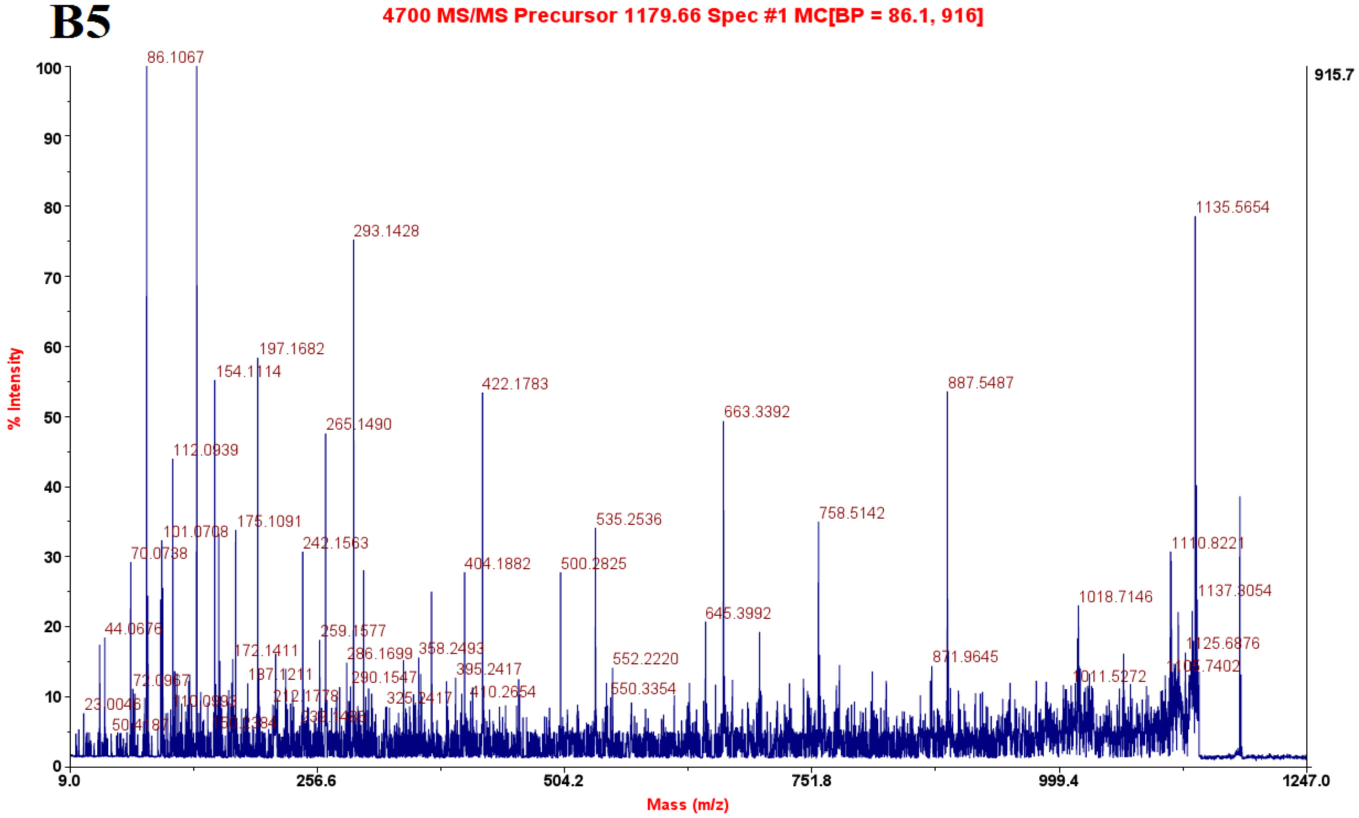
4700 MS/MS Precursor 1179.66 Spec #1 MC[BP = 86.1, 916]
% Intensity
Mass (m/z)
915.7
86.1067
293.1428
1135.5654
197.1682
154.1114
422.1783
887.5487
663.3392
265.1490
112.0939
535.2536
758.5142
101.0708
175.1091
70.0738
242.1563
1110.8221
404.1882
500.2825
1137.8054
1018.7146
645.3992
44.0676
259.1577
172.1411
358.2493
286.1699
871.9645
552.2220
1125.6876
395.2417
187.1211
290.1547
1105.7402
1011.5272
72.0967
212.1778
550.3354
410.2654
325.2417
23.0046
110.0996
50.4187
9.0
256.6
504.2
751.8
999.4
1247.0

B6

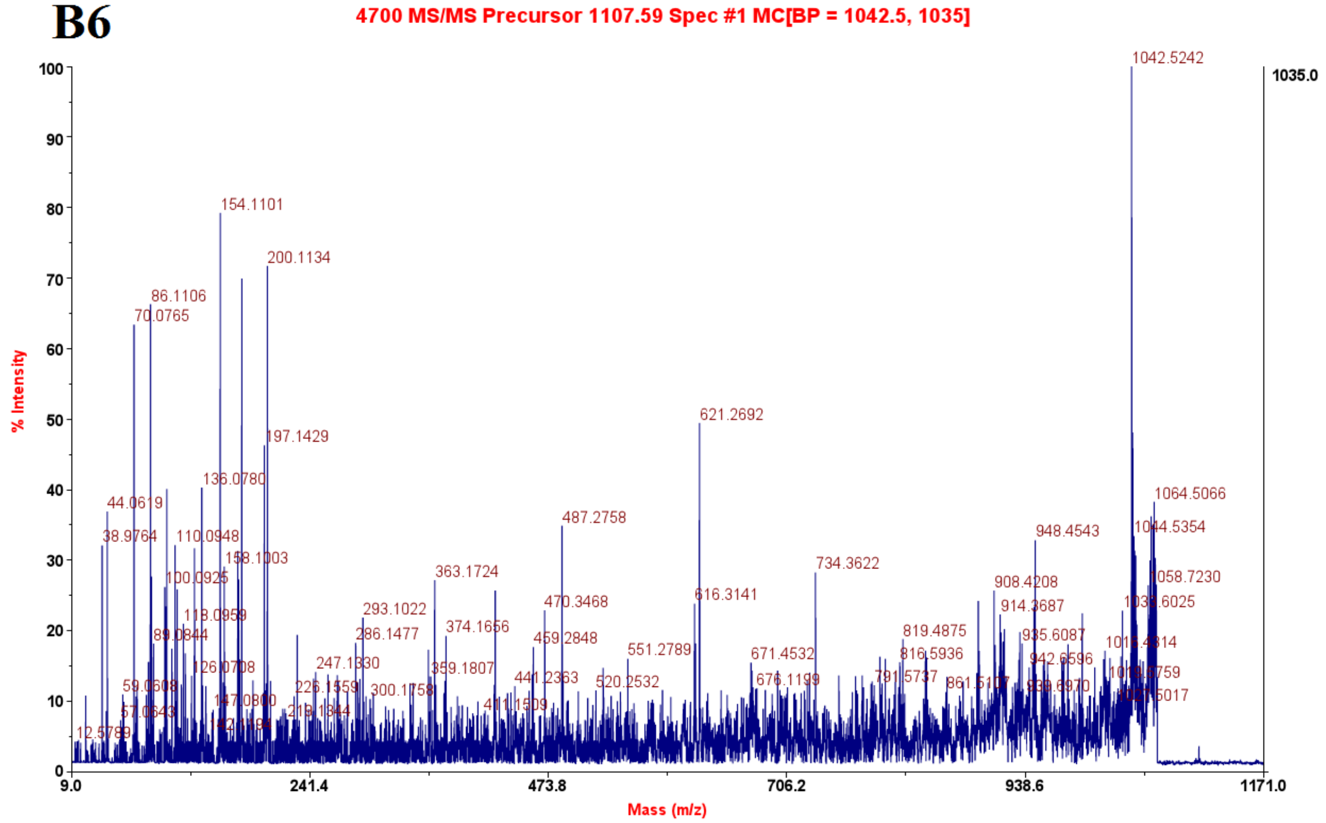
4700 MS/MS Precursor 1107.59 Spec #1 MC[BP = 1042.5, 1035]
% Intensity
Mass (m/z)
1035.0
1042.5242
154.1101
200.1134
86.1106
70.0765
621.2692
197.1429
136.0780
1064.5066
44.0619
487.2758
948.4543
1044.5354
38.9764
110.0948
158.1003
734.3622
363.1724
100.0925
908.4208
1058.7230
616.3141
470.3468
914.3687
118.0959
293.1022
374.1656
819.4875
89.0844
286.1477
459.2848
935.6087
551.2789
816.5936
671.4532
247.1330
126.0708
942.6596
441.2363
520.2532
300.1758
359.1807
676.1199
791.5737
861.5157
59.0608
147.0800
411.1509
57.0643
12.5789
9.0
241.4
473.8
706.2
938.6
1171.0

B7

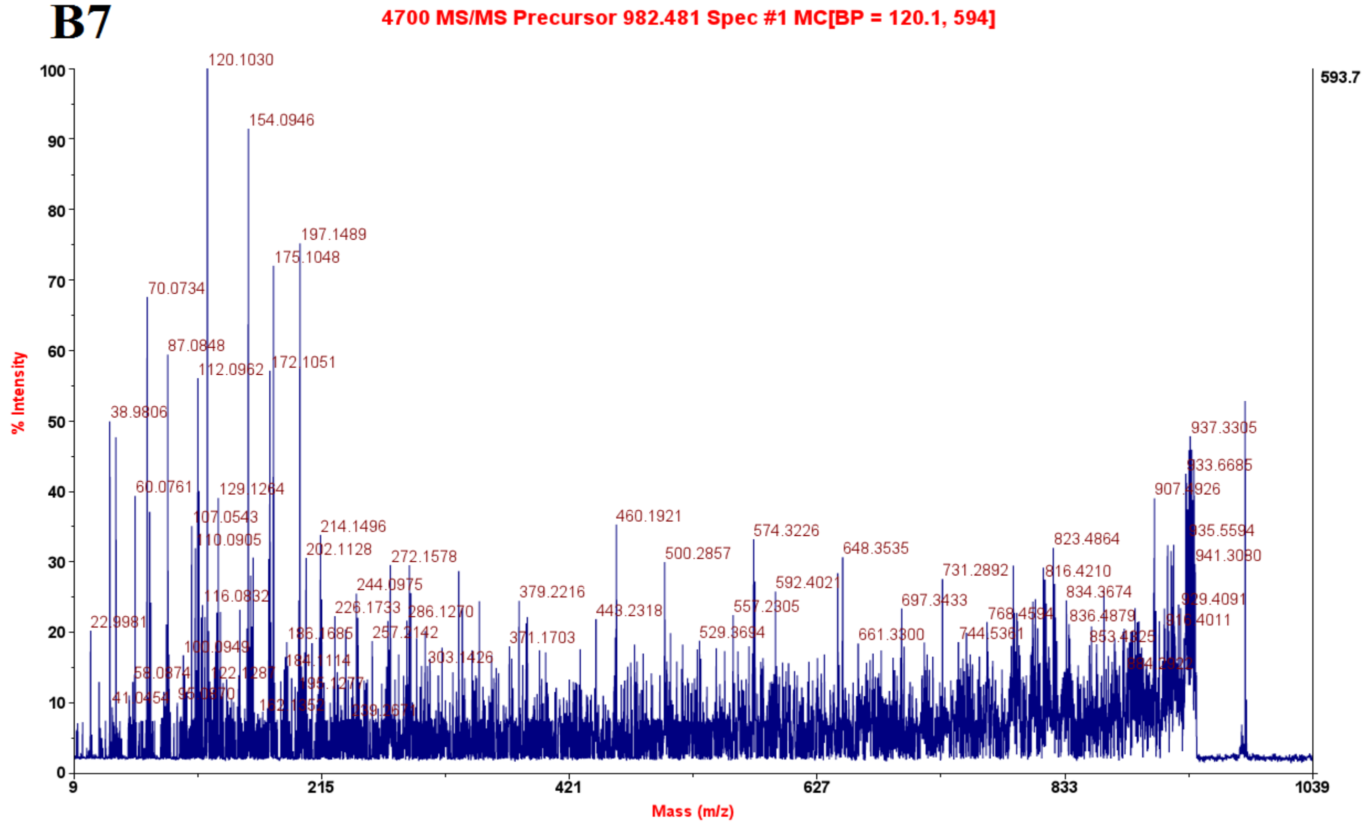
4700 MS/MS Precursor 982.481 Spec #1 MC[BP = 120.1, 594]
% Intensity
Mass (m/z)
593.7
120.1030
154.0946
197.1489
175.1048
70.0734
87.0848
112.0962
172.1051
38.9806
937.3305
60.0761
129.1264
107.0543
460.1921
574.3226
648.3535
823.4864
214.1496
202.1128
272.1578
500.2857
731.2892
816.4210
941.3080
244.0975
592.4021
834.3674
116.0832
379.2216
697.3433
226.1733
286.1270
443.2318
557.2305
768.4594
836.4879
22.9981
529.3694
661.3300
744.5361
371.1703
303.1426

B8

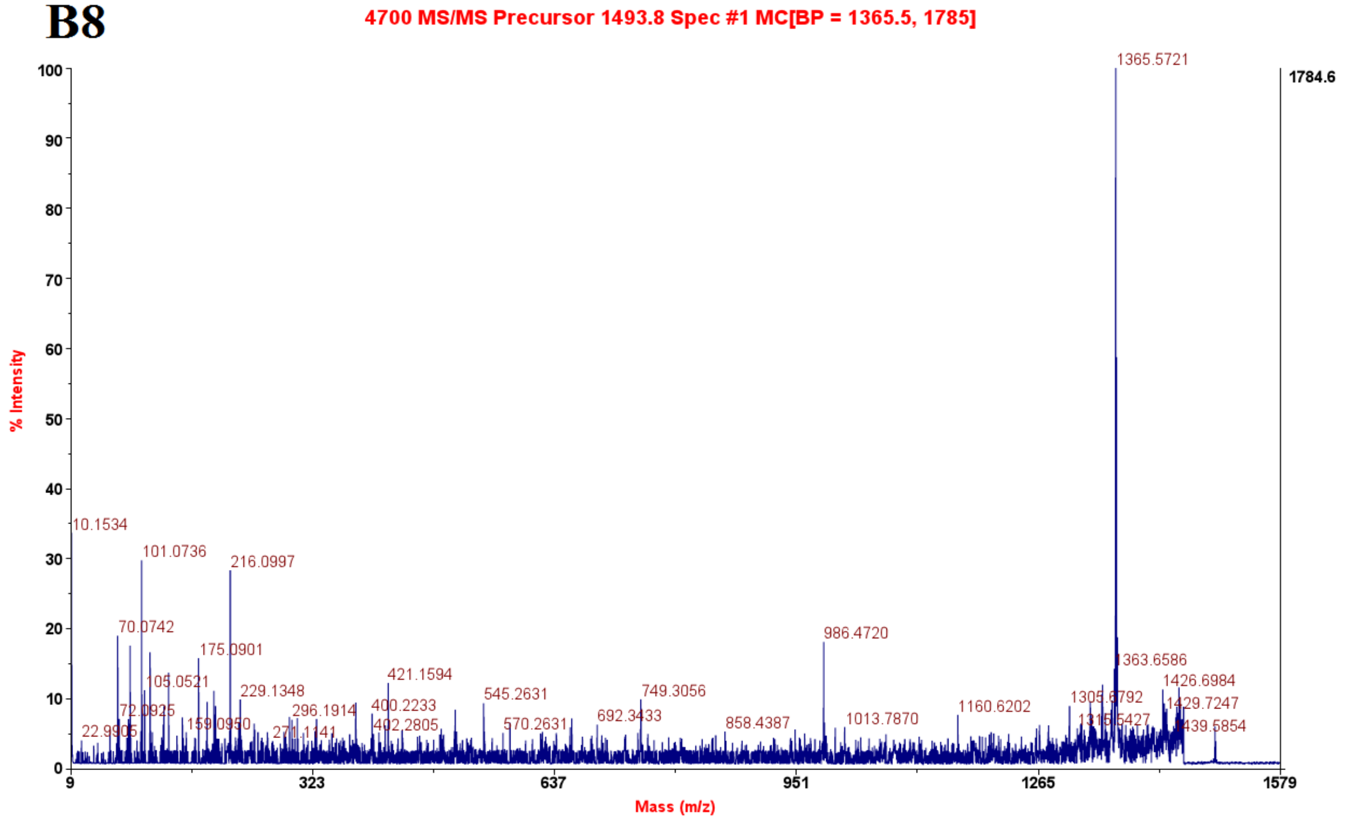
4700 MS/MS Precursor 1493.8 Spec #1 MC[BP = 1365.5, 1785]
% Intensity
Mass (m/z)
1784.6
1365.5721
101.0736
216.0997
70.0742
986.4720
175.0901
1363.6586
421.1594
1426.6984
105.0521
229.1348
545.2631
749.3056
1429.7247
296.1914
400.2233
1160.6202
692.3433
1013.7870
858.4387
402.2805
570.2631
159.0950
1439.5854

B9

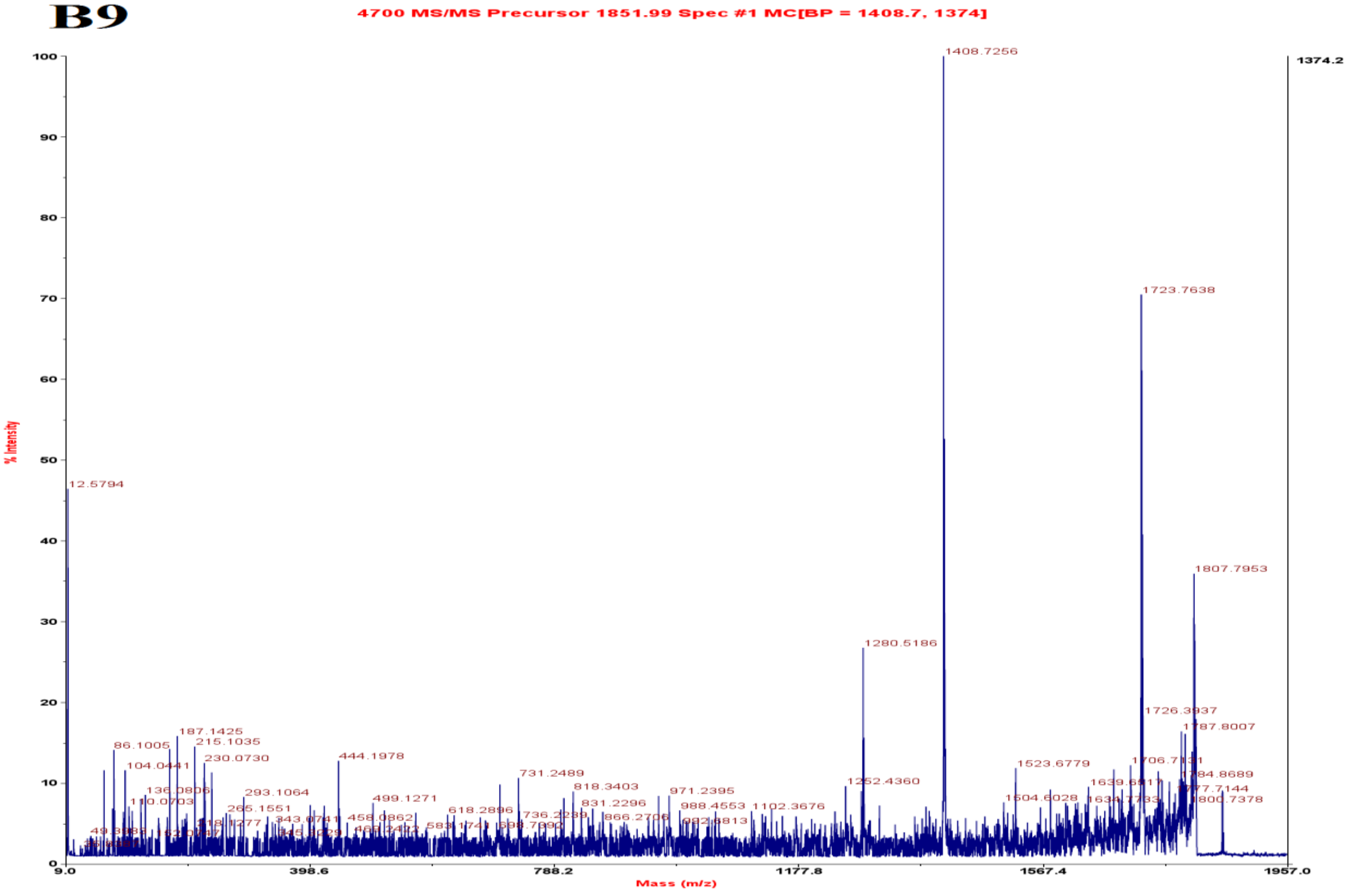
4700 MS/MS Precursor 1851.99 Spec #1 MC[BP = 1408.7, 1374]
1374.2
% Intensity
Mass (m/z)
9.0
398.6
788.2
1177.8
1567.4
1957.0
1408.7256
1723.7638
1807.7953
1280.5186
1726.3937
1787.8007
12.5794
187.1425
215.1035
86.1005
230.0730
104.0441
444.1978
731.2489
818.3403
971.2395
1252.4360
1523.6779
293.1064
499.1271
831.2296
988.4553
1102.3676
265.1551
618.2896
1604.6028

B10

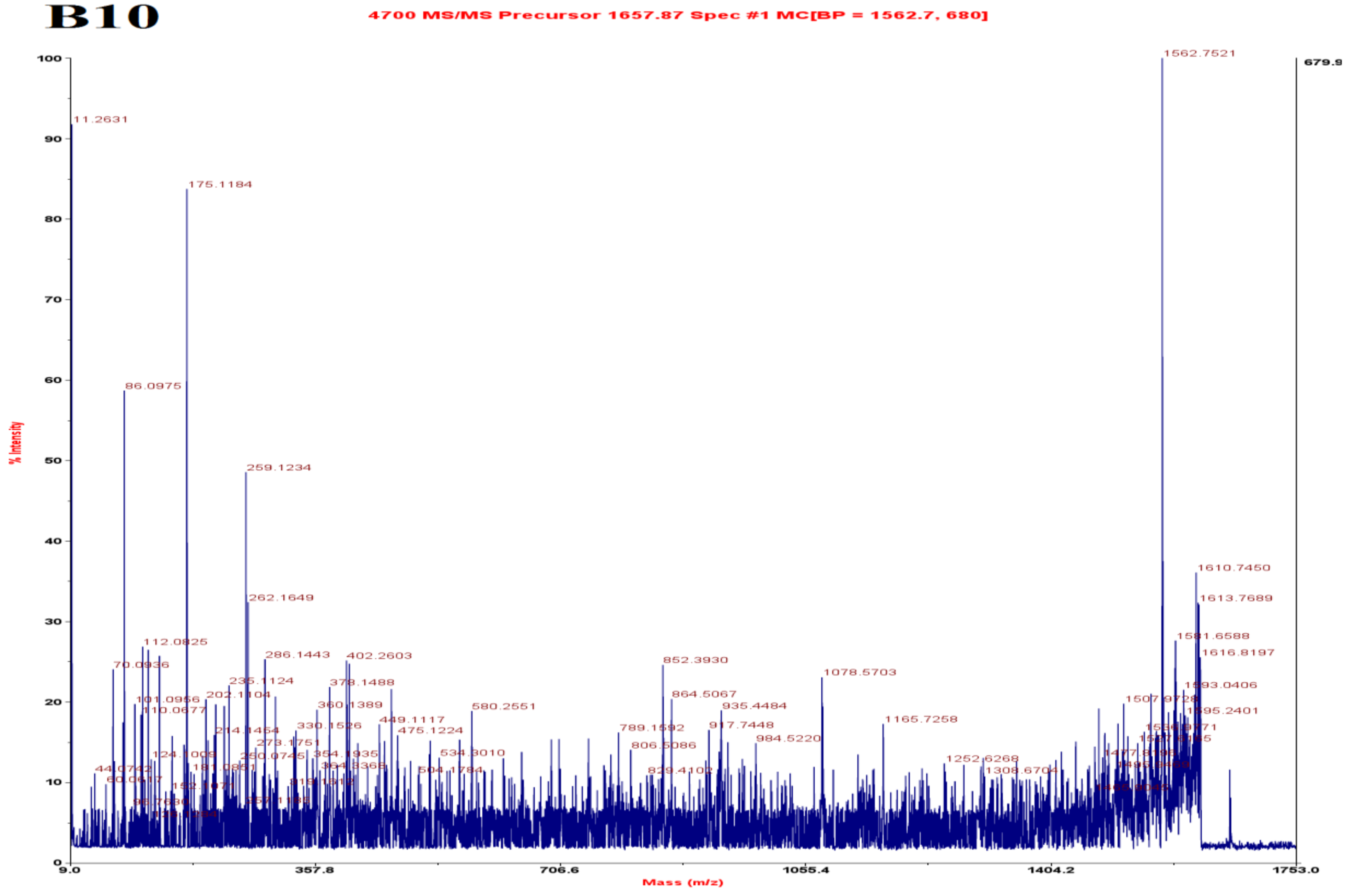
4700 MS/MS Precursor 1657.87 Spec #1 MC[BP = 1562.7, 680]
679.9
% Intensity
Mass (m/z)
9.0
357.8
706.6
1055.4
1404.2
1753.0
1562.7521
11.2631
175.1184
86.0975
259.1234
1610.7450
1613.7689
262.1649
112.0825
286.1443
402.2603
852.3930
1078.5703
1616.8197
70.0936
235.1124
378.1488
202.1104
864.5067
935.4484
580.2551
1165.7258
449.1117
475.1224
917.7448
789.1592
806.5086
984.5220
1252.6268
1308.6704
534.3010

B11

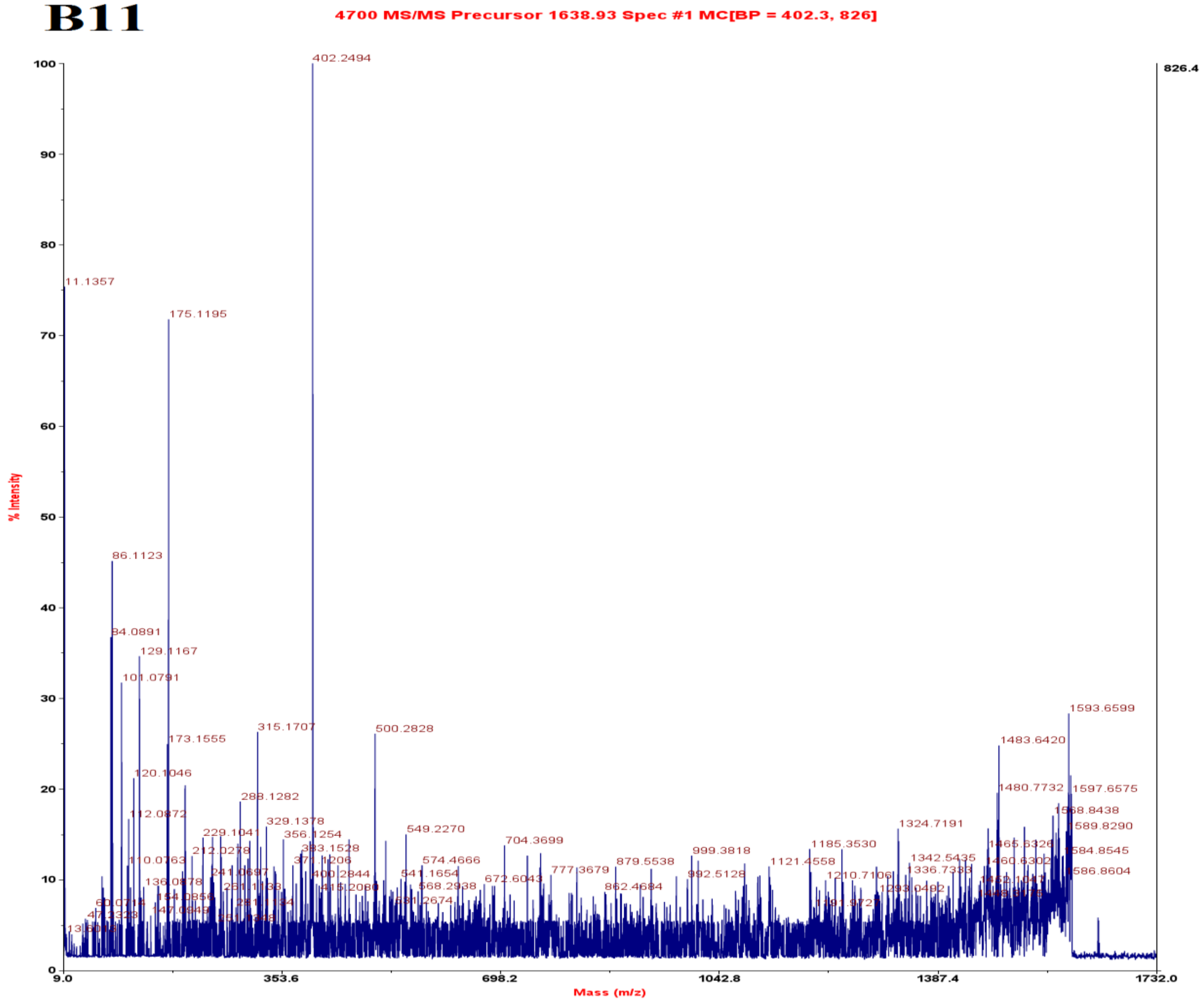

4700 MS/MS Precursor 1638.93 Spec #1 MC[BP = 402.3, 826]
% Intensity
Mass (m/z)
826.4
402.2494
175.1195
86.1123
84.0891
129.1167
101.0791
315.1707
500.2828
173.1555
120.1046
288.1282
329.1378
549.2270
704.3699
1593.6599
1483.6420
1480.7732
1597.6575
1324.7191
1185.3530
999.3818
879.5538
1121.4558
574.4666

B12

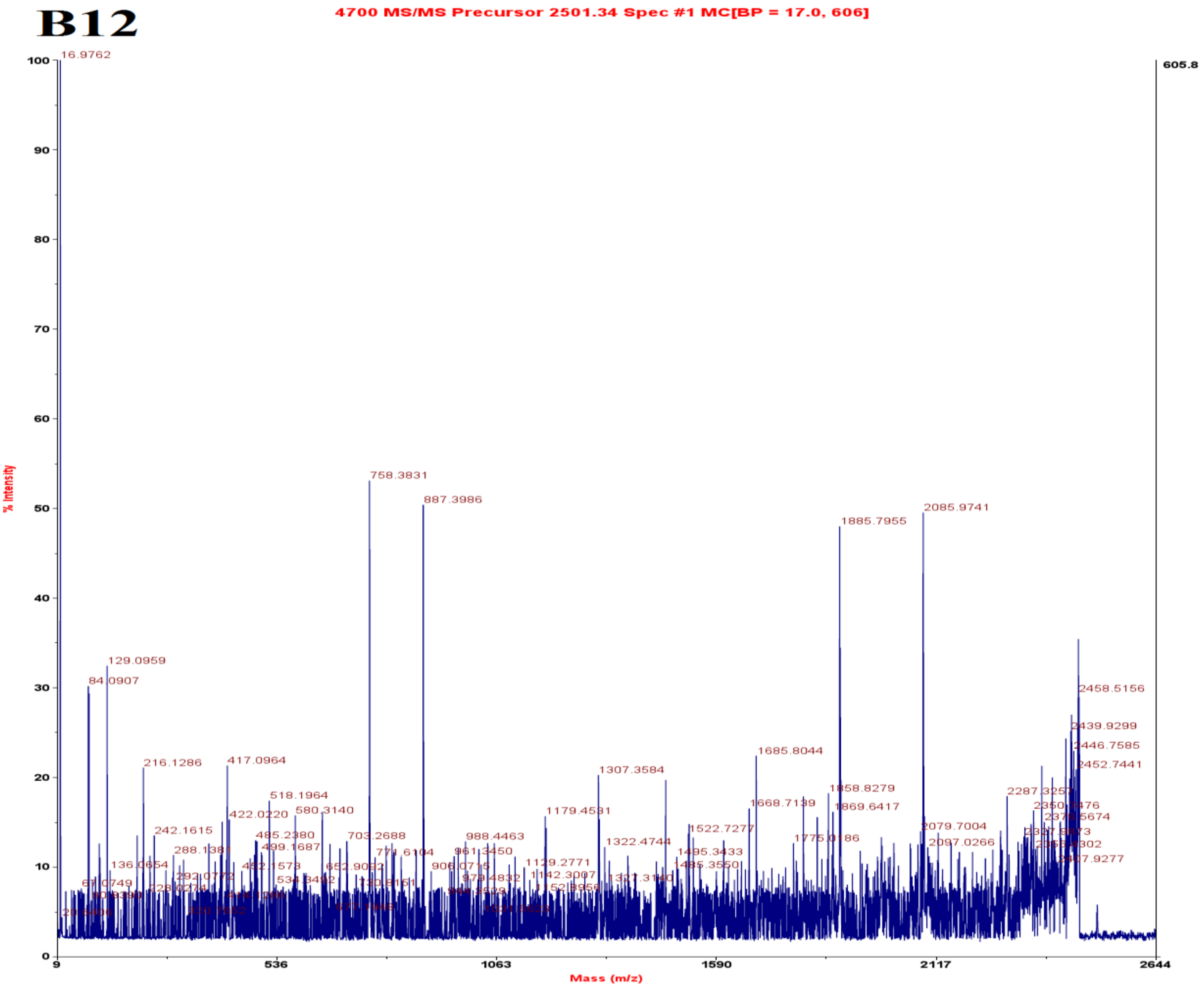

Fig. S3 Mass spectra of spot (1087) in resting cyst

A: Peptide mass fingerprinting of type II cytoskeletal (1087) in resting cyst; B1-B12: MS/MS spectrum of type II cytoskeletal (1087) in resting cyst.
