# Supplementary material for: Proteomic Approach to Reveal the Proteins Associated with Encystment of the Ciliate Euplotes encysticus
Source: PLoS One. 2014 May 16;9(5):e97362. doi: 10.1371/journal.pone.0097362 (PMC4023950; doi:10.1371/journal.pone.0097362)
Supplement: Figure S4 — Mass spectra of spot (1093) in resting cyst. A: Peptide mass fingerprinting of Calpain-like protein (1093) in resting cyst; B: MS/MS spectrum of Calpain-like protein (1093) in resting cyst. (PDF) [file pone.0097362.s004.pdf]

A

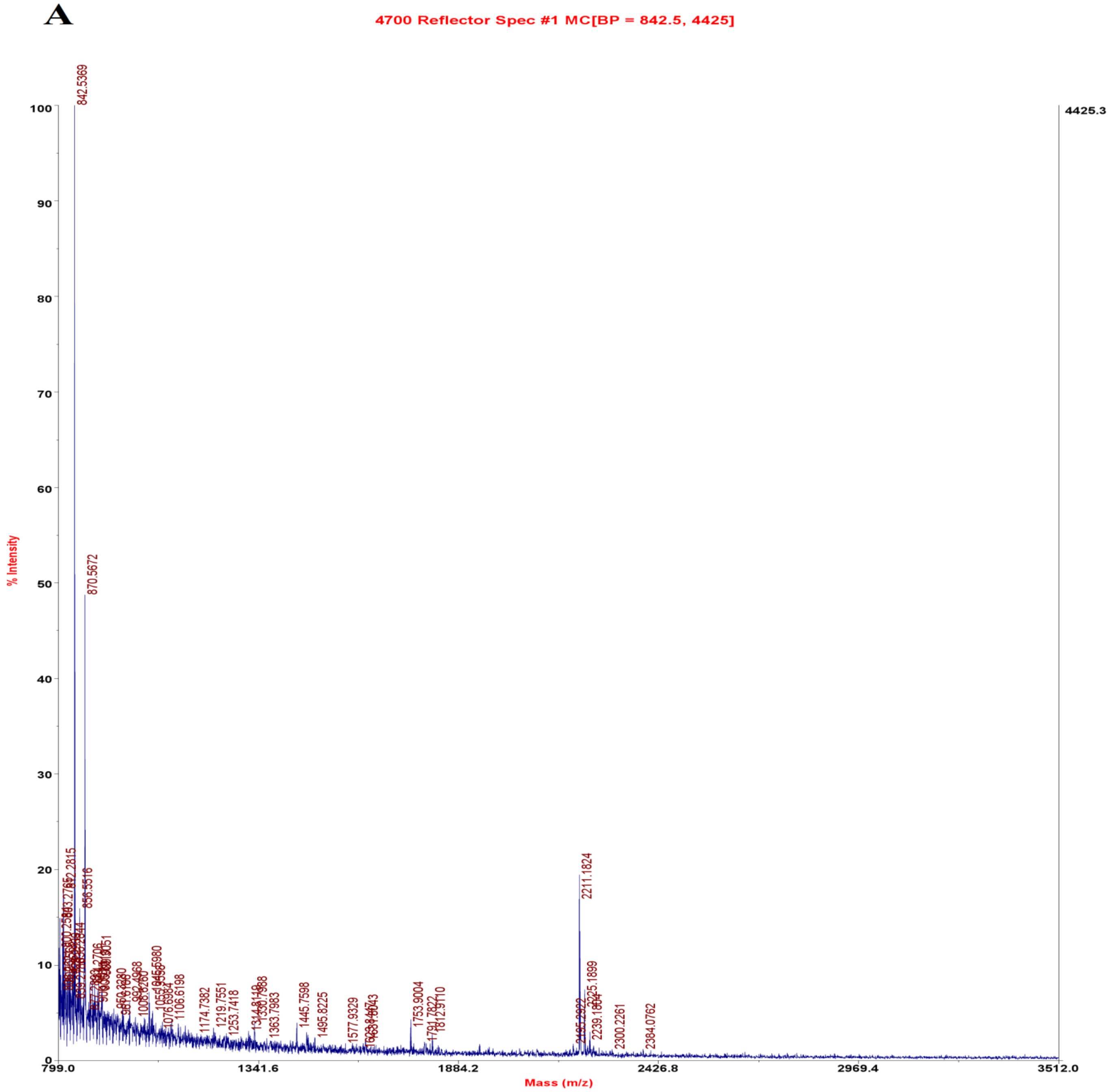
4700 Reflector Spec #1 MC[BP = 842.5, 4425]
% Intensity
Mass (m/z)
4425.3
842.5369
870.5672
856.5516
2211.1824
2225.1899
2239.1604
2300.2261
2384.0762
1753.9004
1812.9110
1577.9329
1495.8225
1445.7598
1363.7983
1253.7418
1219.7551
1174.7382
1106.6198
1076.5984
992.4968
959.3380
799.0
1341.6
1884.2
2426.8
2969.4
3512.0

B

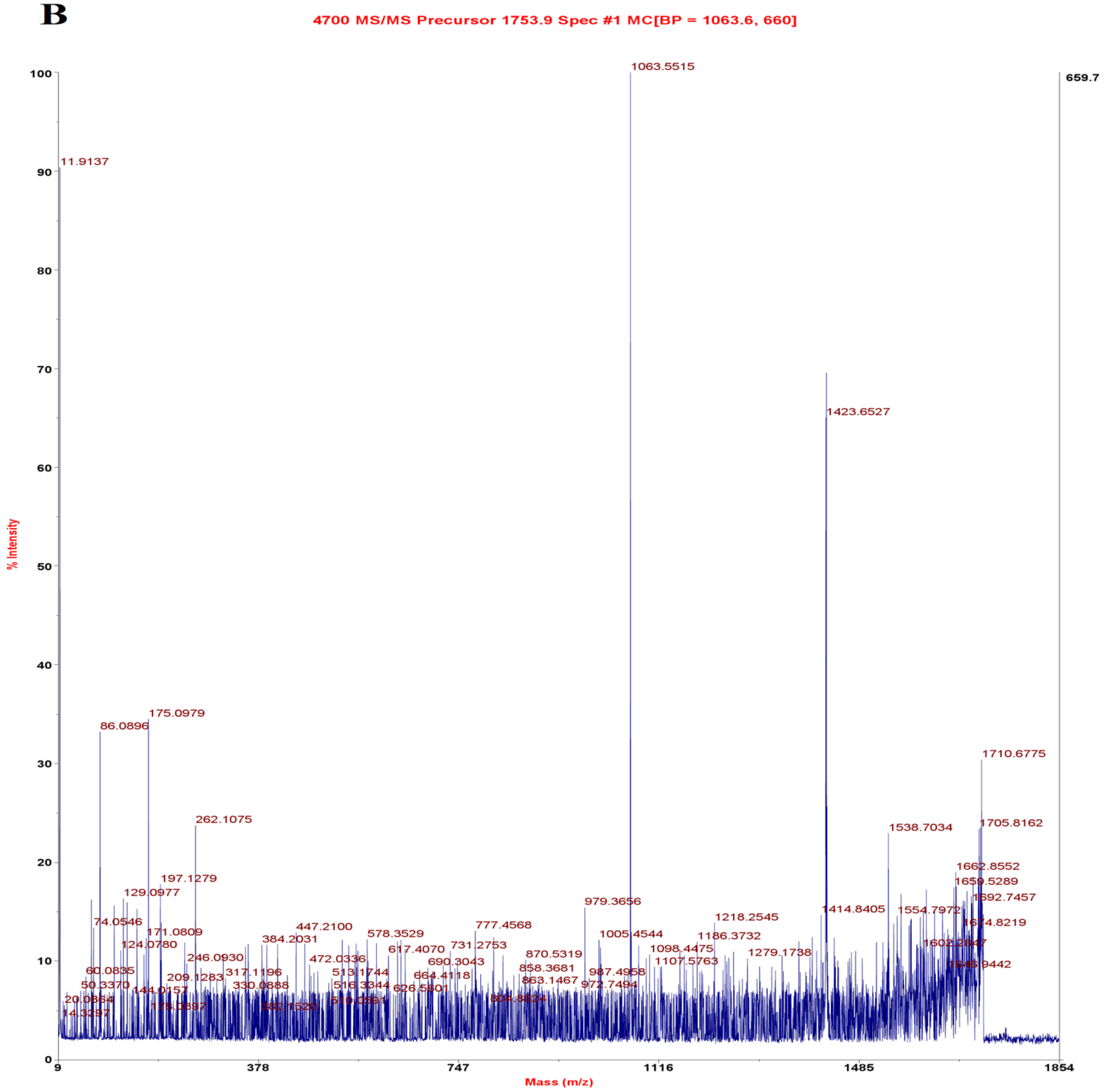

Fig. S4 Mass spectra of spot (1093) in resting cyst

A: Peptide mass fingerprinting of Calpain-like protein (1093) in resting cyst; B: MS/MS spectrum of Calpain-like protein (1093) in resting cyst.
