# Supplementary material for: Proteomic Approach to Reveal the Proteins Associated with Encystment of the Ciliate Euplotes encysticus
Source: PLoS One. 2014 May 16;9(5):e97362. doi: 10.1371/journal.pone.0097362 (PMC4023950; doi:10.1371/journal.pone.0097362)
Supplement: Figure S5 — Mass spectra of spot (1096) in resting cyst. A: Peptide mass fingerprinting of hypothetical protein IMG5 (1096) in resting cyst; B1-B5: MS/MS spectrum of hypothetical protein IMG5 (1096) in resting cyst. (PDF) [file pone.0097362.s005.pdf]

A

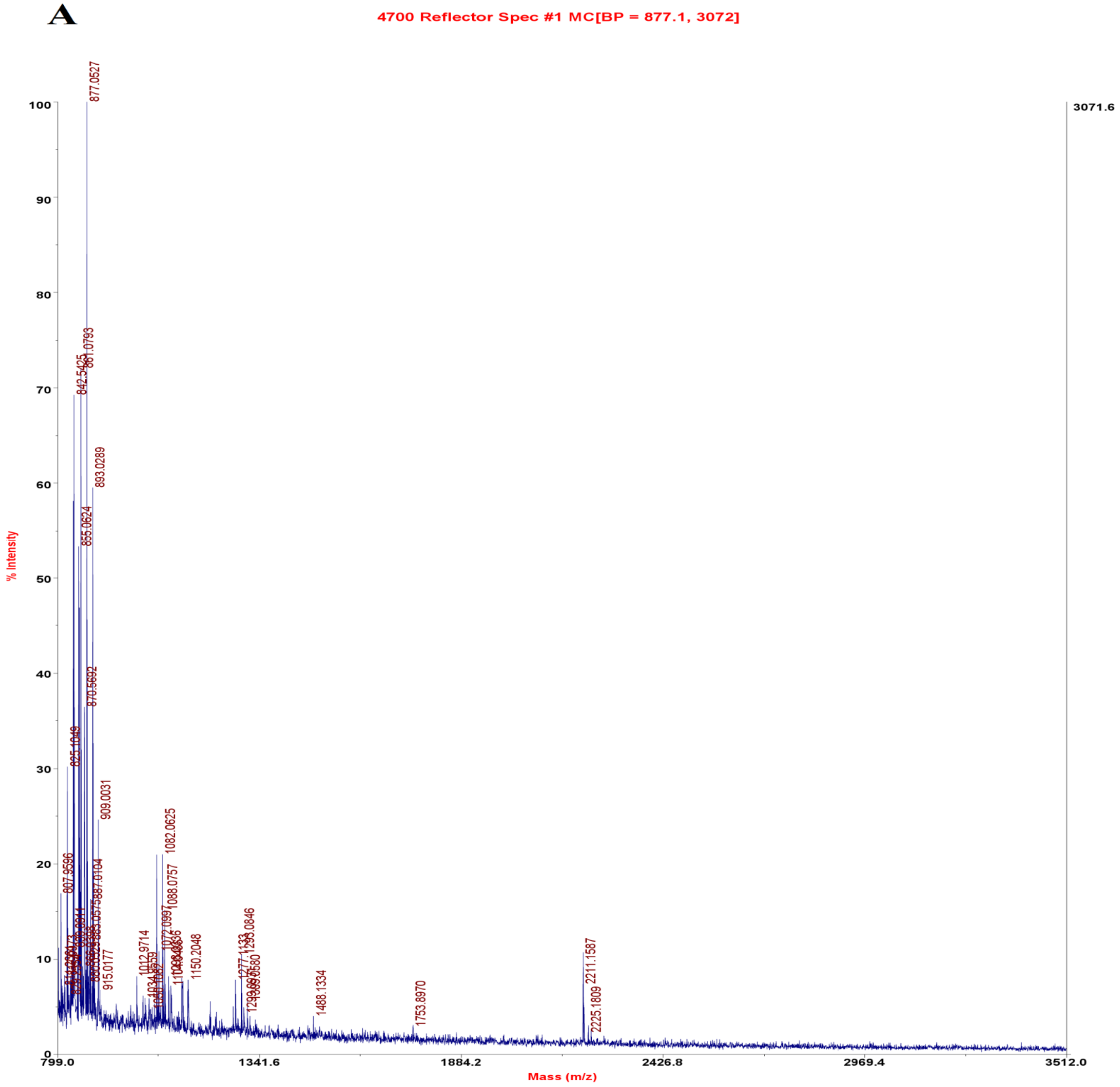
4700 Reflector Spec #1 MC[BP = 877.1, 3072]
% Intensity
Mass (m/z)
3071.6
877.0527
861.0793
842.5425
893.0289
855.0624
870.5692
825.1049
909.0031
807.9596
887.0104
915.0177
1082.0625
1088.0757
1012.9714
1150.2048
1293.0846
1488.1334
1753.8970
2211.1587
2225.1809
799.0
1341.6
1884.2
2426.8
2969.4
3512.0

B1

4700 MS/MS Precursor 1082.06 Spec #1 MC[BP = 893.0, 1302]

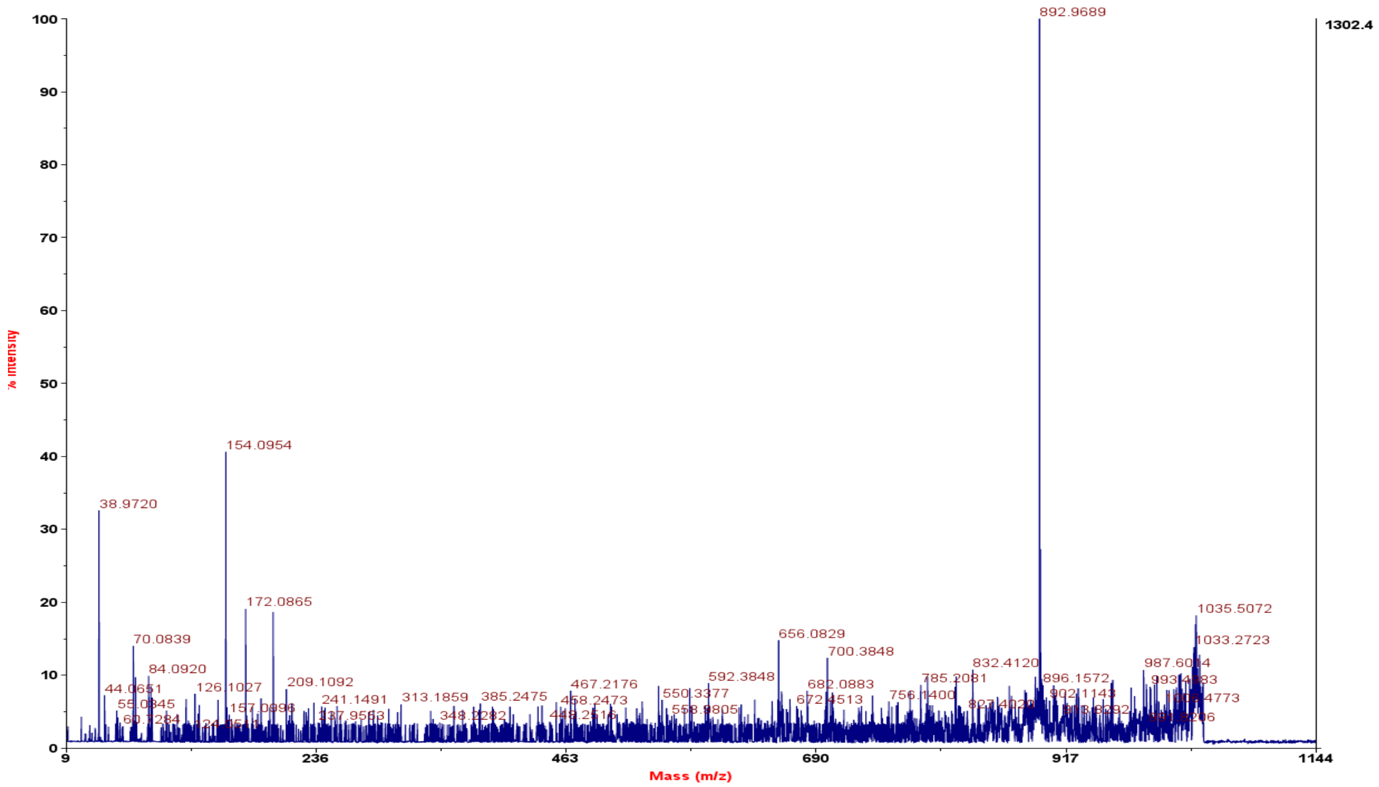

B2

4700 MS/MS Precursor 1066.09 Spec #1 MC[BP = 877.0, 1415]

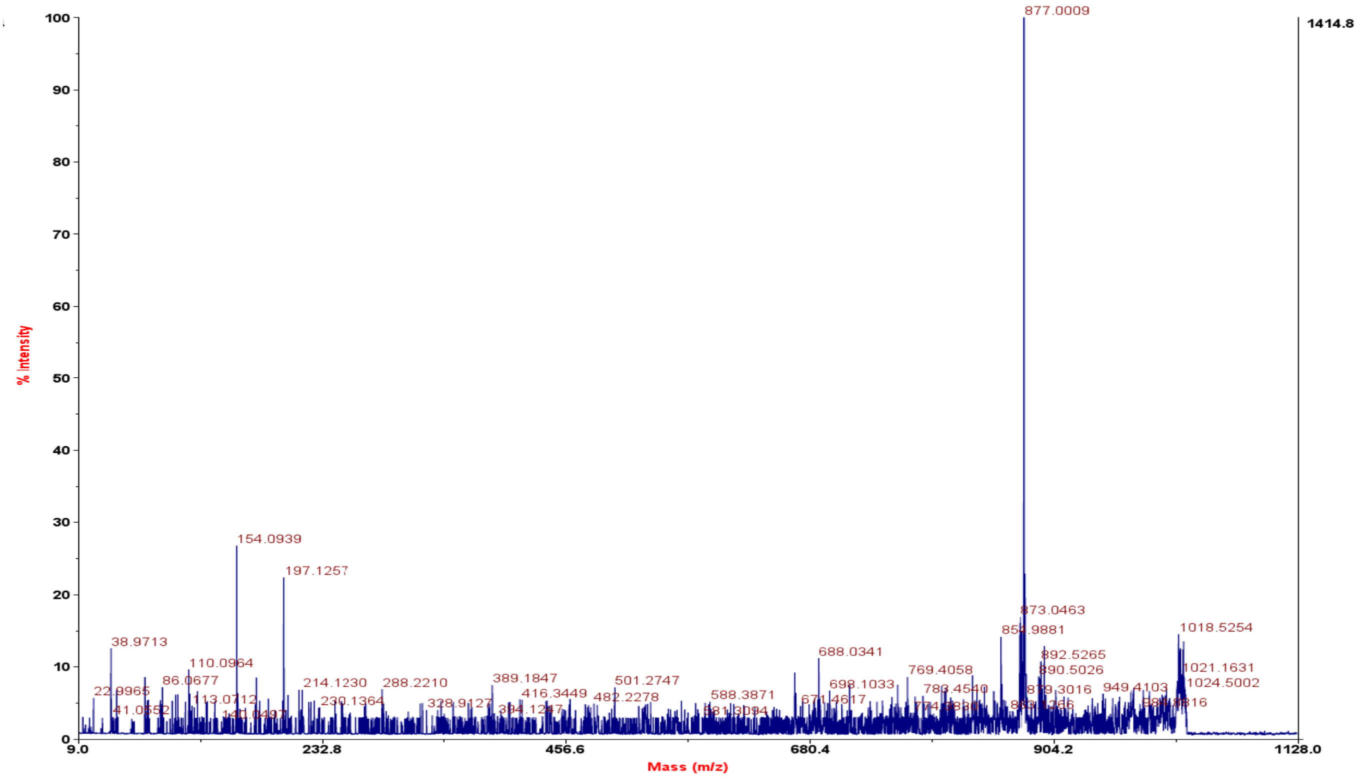

B3

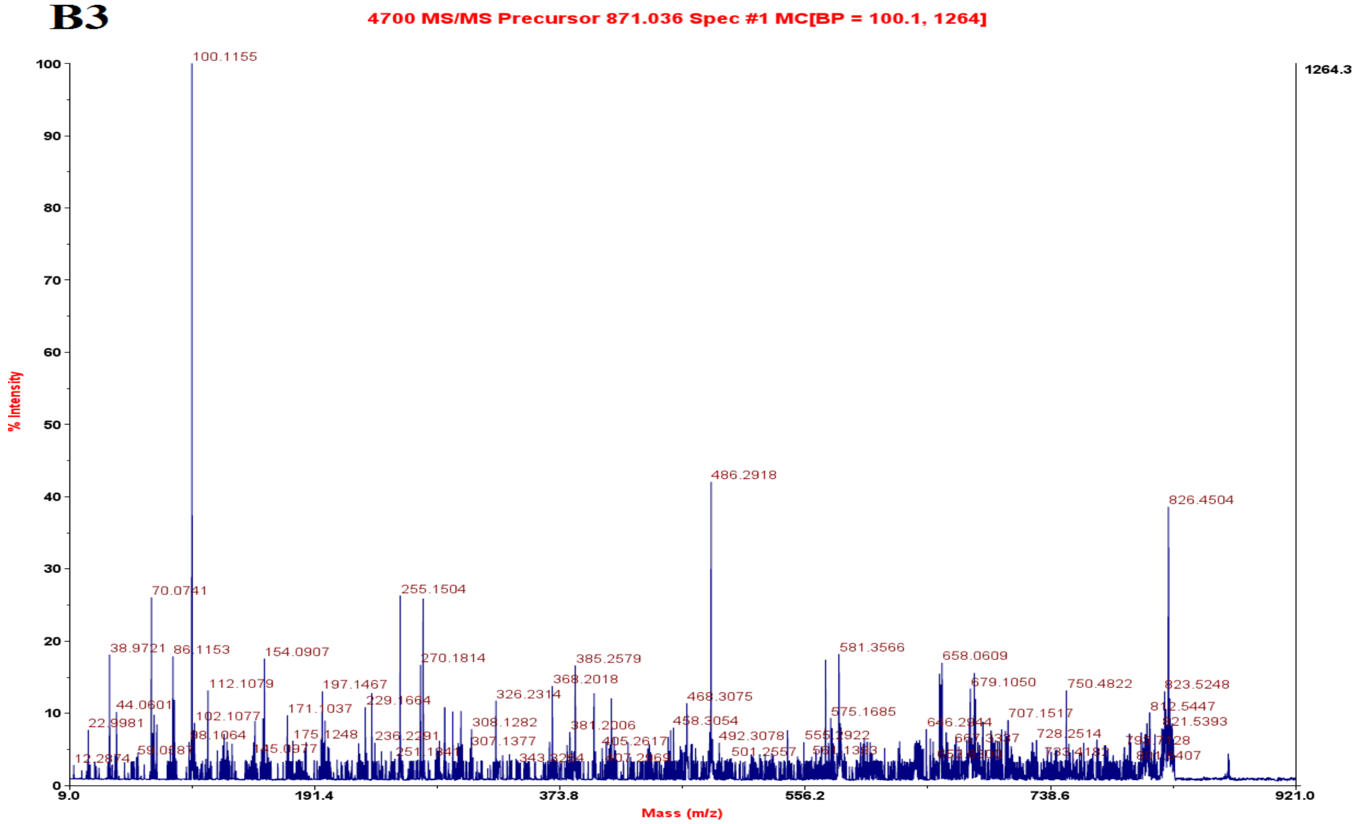
4700 MS/MS Precursor 871.036 Spec #1 MC[BP = 100.1, 1264]
% Intensity
Mass (m/z)
1264.3
100.1155
486.2918
826.4504
70.0741
255.1504
38.9721
86.1153
154.0907
581.3566
658.0609
270.1814
385.2579
368.2018
112.1079
197.1467
679.1050
750.4822
823.5248
326.2314
468.3075
229.1664
44.0601
171.1037
812.5447
575.1685
707.1517
102.1077
22.9981
381.2006
308.1282
458.3054
646.2844
821.5393
175.1248
236.2291
492.3078
728.2514
307.1377
405.2617
9.0
191.4
373.8
556.2
738.6
921.0

B4

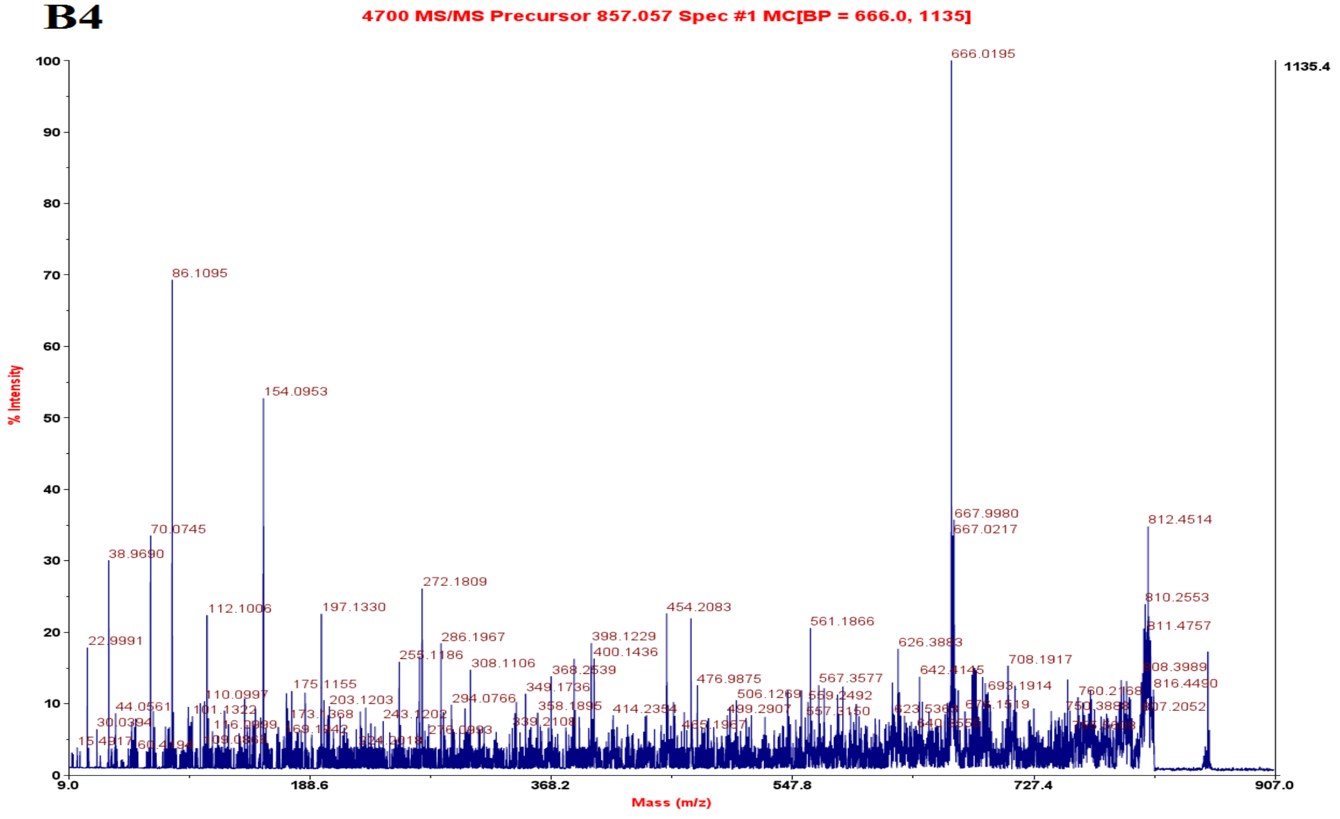
4700 MS/MS Precursor 857.057 Spec #1 MC[BP = 666.0, 1135]
% Intensity
Mass (m/z)
1135.4
666.0195
86.1095
154.0953
667.9980
667.0217
812.4514
70.0745
38.9690
272.1809
112.1006
197.1330
454.2083
810.2553
561.1866
22.9991
286.1967
398.1229
811.4757
626.3883
255.1186
400.1436
308.1106
708.1917
368.2539
642.4145
808.3989
175.1155
476.9875
567.3577
349.1736
816.4490
693.1914
110.0997
506.1269
760.2168
203.1203
294.0766
44.0561
358.1895
414.2354
499.2907
101.1322
243.1202
9.0
188.6
368.2
547.8
727.4
907.0

B5

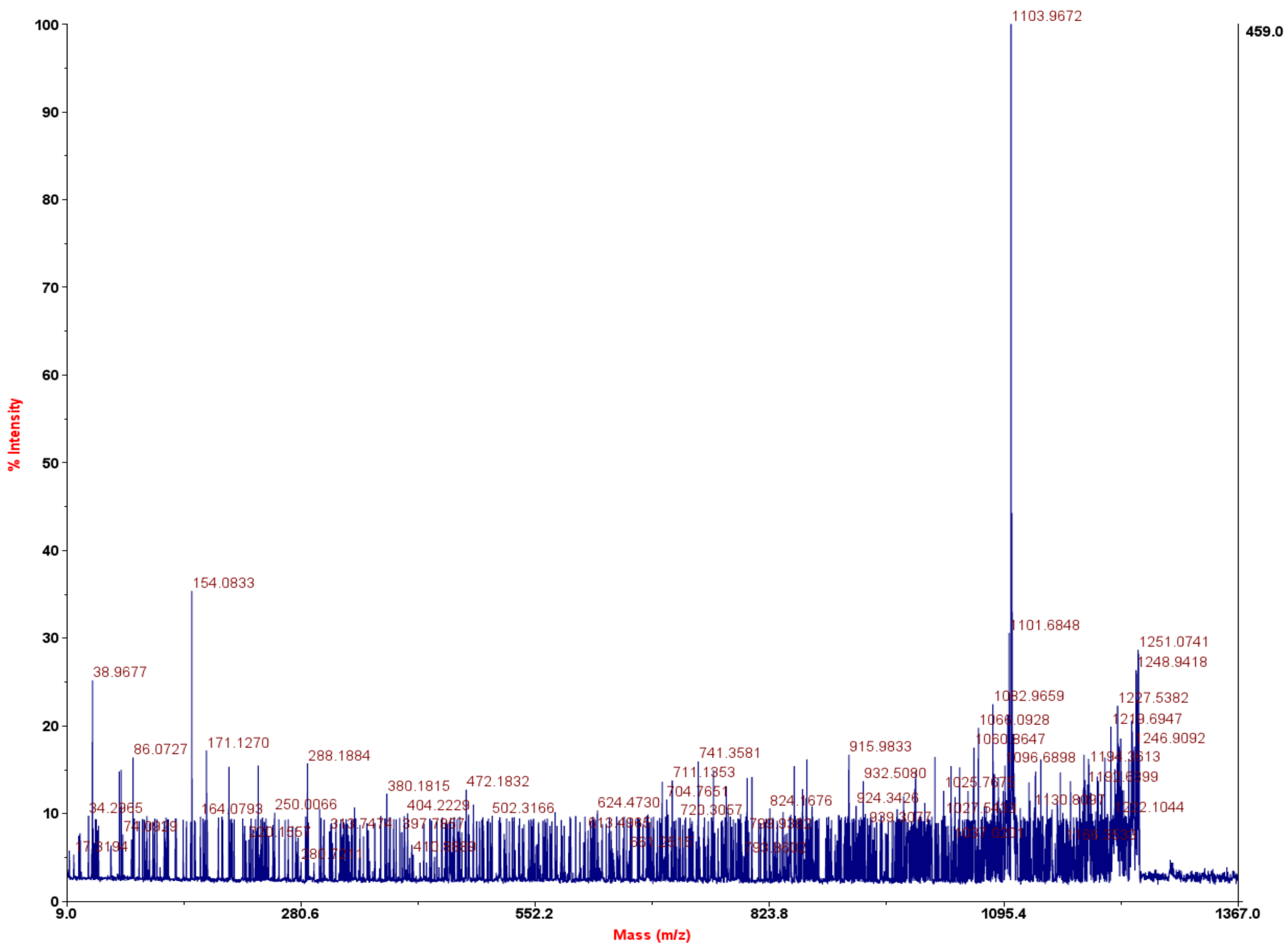

Fig. S5 Mass spectra of spot (1096) in resting cyst

A: Peptide mass fingerprinting of hypothetical protein IMG5 (1096) in resting cyst; B1-B5: MS/MS spectrum of hypothetical protein IMG5 (1096) in resting cyst.
