# Supplementary material for: Proteomic Approach to Reveal the Proteins Associated with Encystment of the Ciliate Euplotes encysticus
Source: PLoS One. 2014 May 16;9(5):e97362. doi: 10.1371/journal.pone.0097362 (PMC4023950; doi:10.1371/journal.pone.0097362)
Supplement: Figure S6 — Mass spectra of spot (1118) in resting cyst. A: Peptide mass fingerprinting of hypothetical protein OXYTRI (1118) in resting cyst; B: MS/MS spectrum of hypothetical protein OXYTRI (1118) in resting cyst. (PDF) [file pone.0097362.s006.pdf]

A

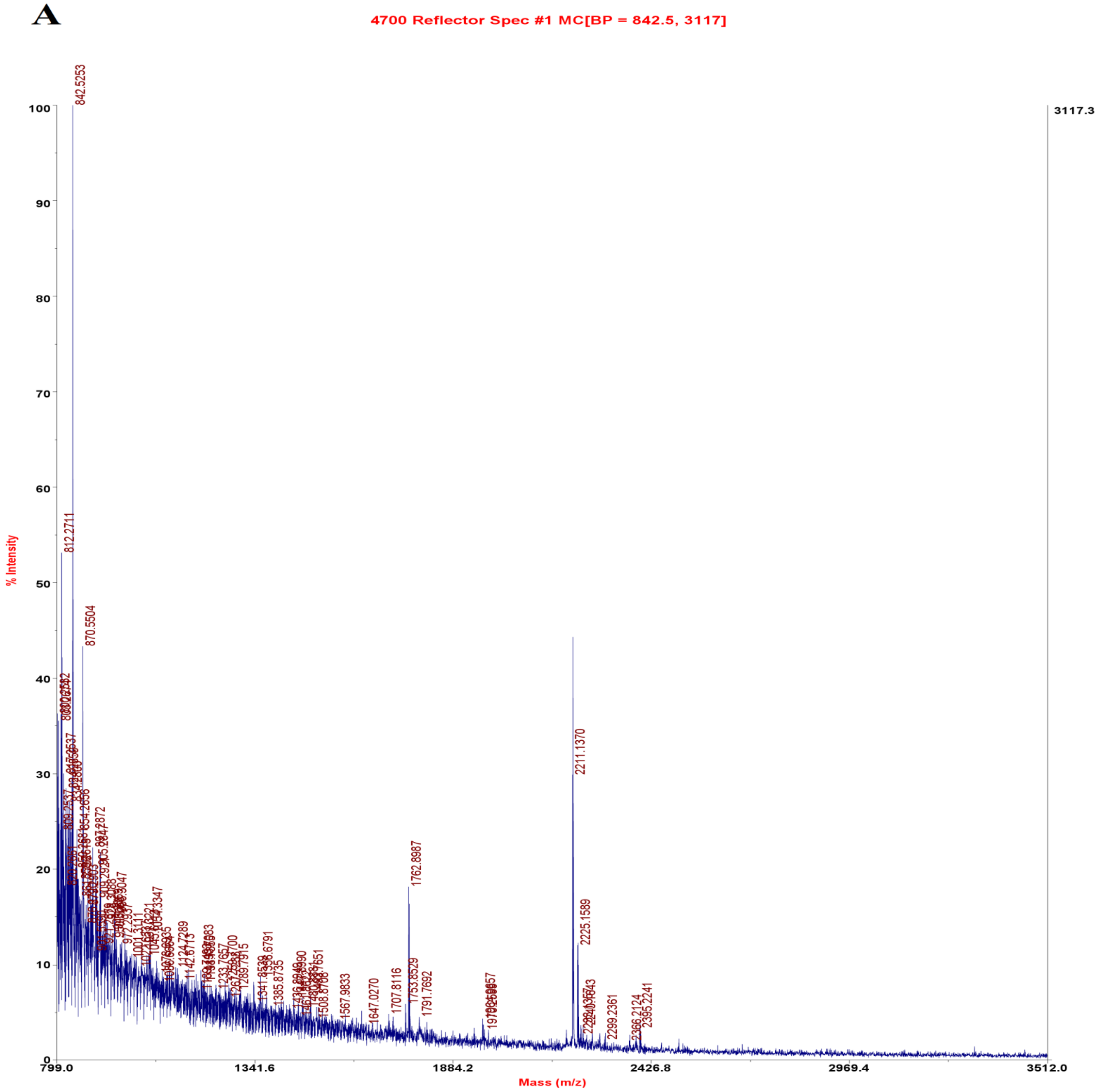
4700 Reflector Spec #1 MC[BP = 842.5, 3117]
% Intensity
Mass (m/z)
3117.3
842.5253
812.2711
870.5504
2211.1370
1762.8987
2225.1589
799.0
1341.6
1884.2
2426.8
2969.4
3512.0

B

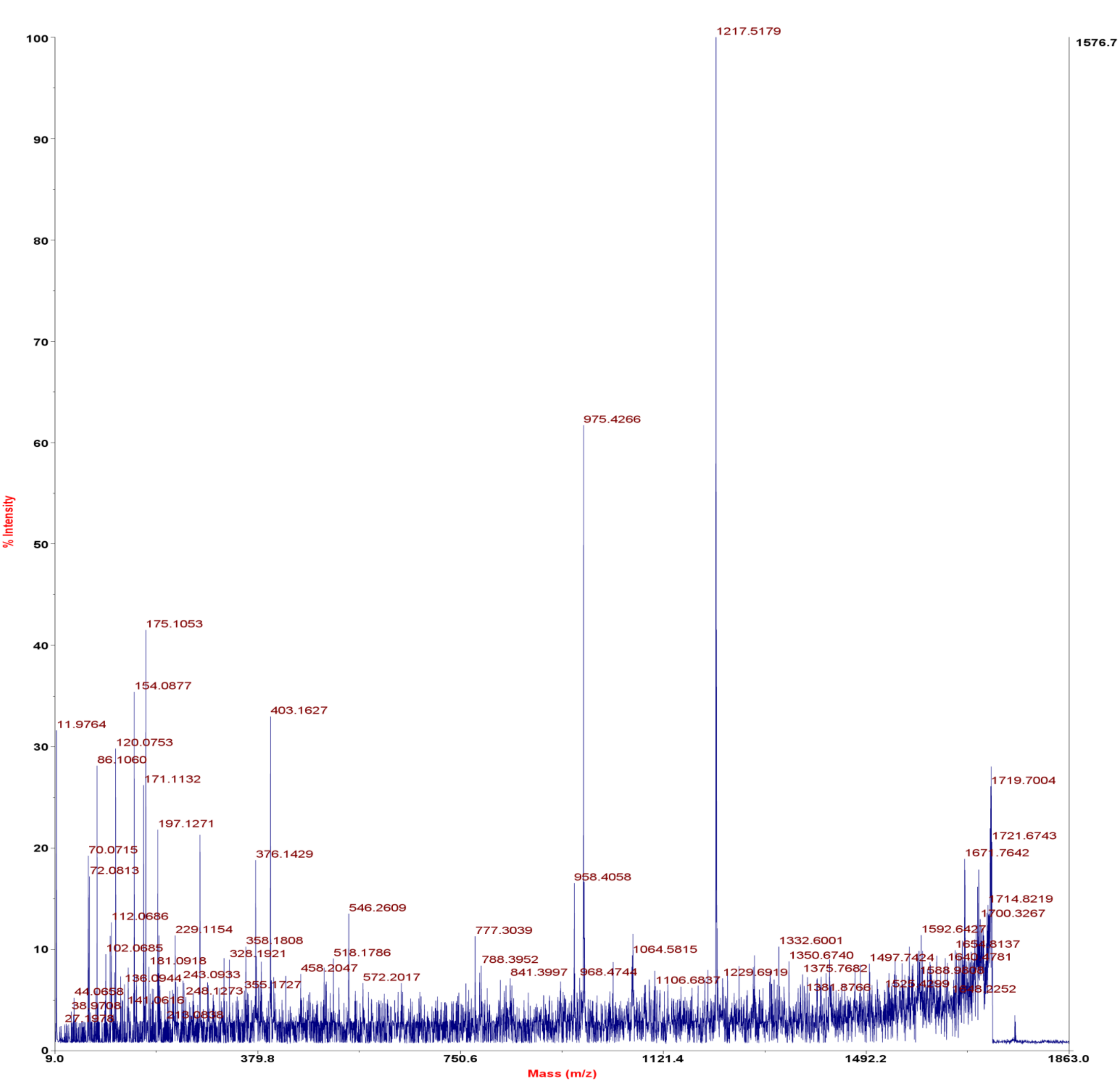

Fig. S6 Mass spectra of spot (1118) in resting cyst

A: Peptide mass fingerprinting of hypothetical protein OXYTRI (1118) in resting cyst; B: MS/MS spectrum of hypothetical protein OXYTRI (1118) in resting cyst.
