# Supplementary material for: Proteomic Approach to Reveal the Proteins Associated with Encystment of the Ciliate Euplotes encysticus
Source: PLoS One. 2014 May 16;9(5):e97362. doi: 10.1371/journal.pone.0097362 (PMC4023950; doi:10.1371/journal.pone.0097362)
Supplement: Figure S7 — Mass spectra of spot (1120) in resting cyst. A: Peptide mass fingerprinting of ribosomal protein-serine acetyltransferase (1120) in resting cyst; B: MS/MS spectrum of ribosomal protein-serine acetyltransferase (1120) in resting cyst. (PDF) [file pone.0097362.s007.pdf]

A

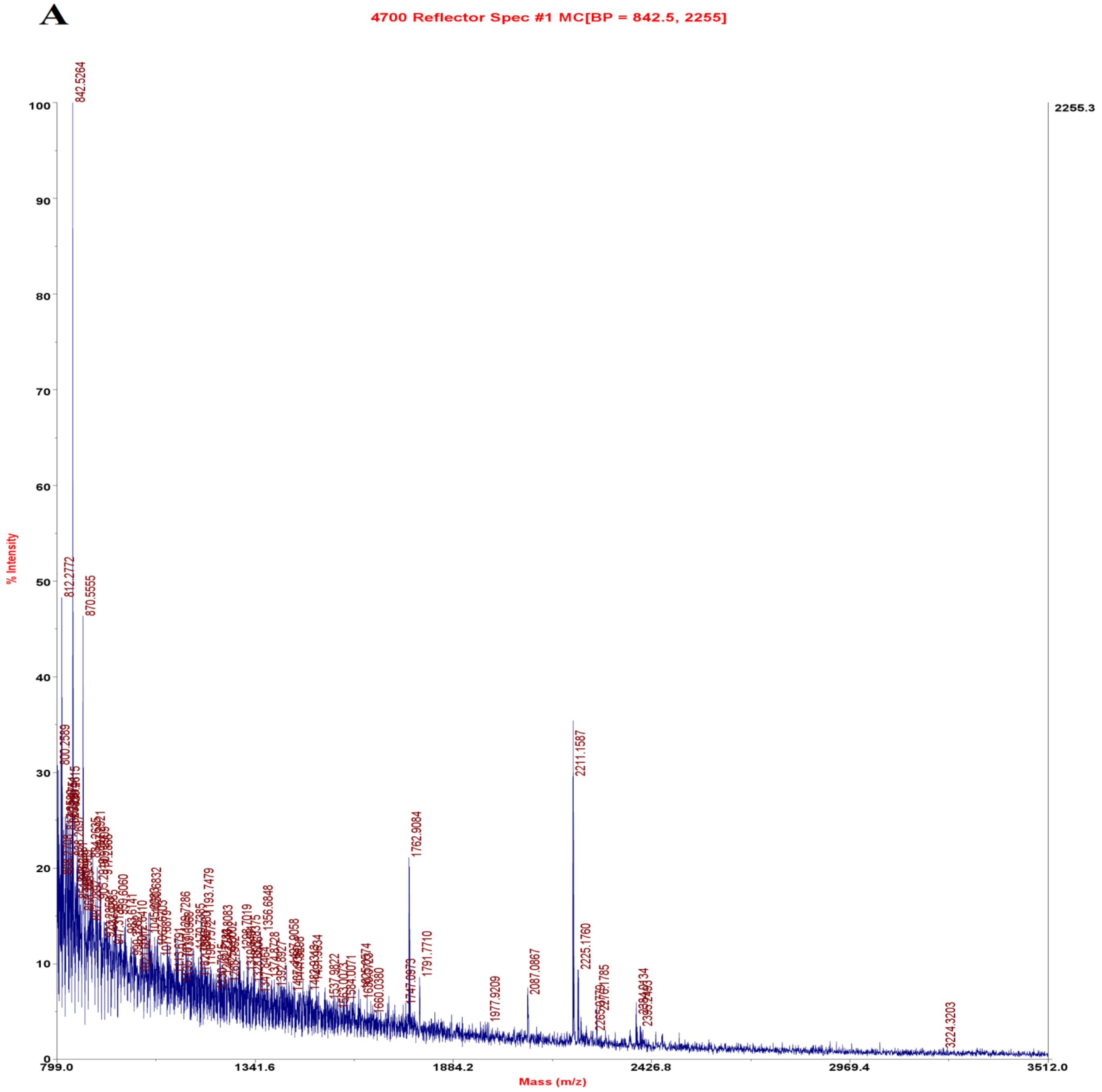
4700 Reflector Spec #1 MC[BP = 842.5, 2255]
% Intensity
Mass (m/z)
2255.3
799.0
1341.6
1884.2
2426.8
2969.4
3512.0
842.5264
812.2772
870.5555
800.2569
1762.9084
1791.7710
1747.0873
2211.1587
2225.1760
2087.0867
1977.9209
3224.3203
1660.0380
1356.6848
1193.7479

B

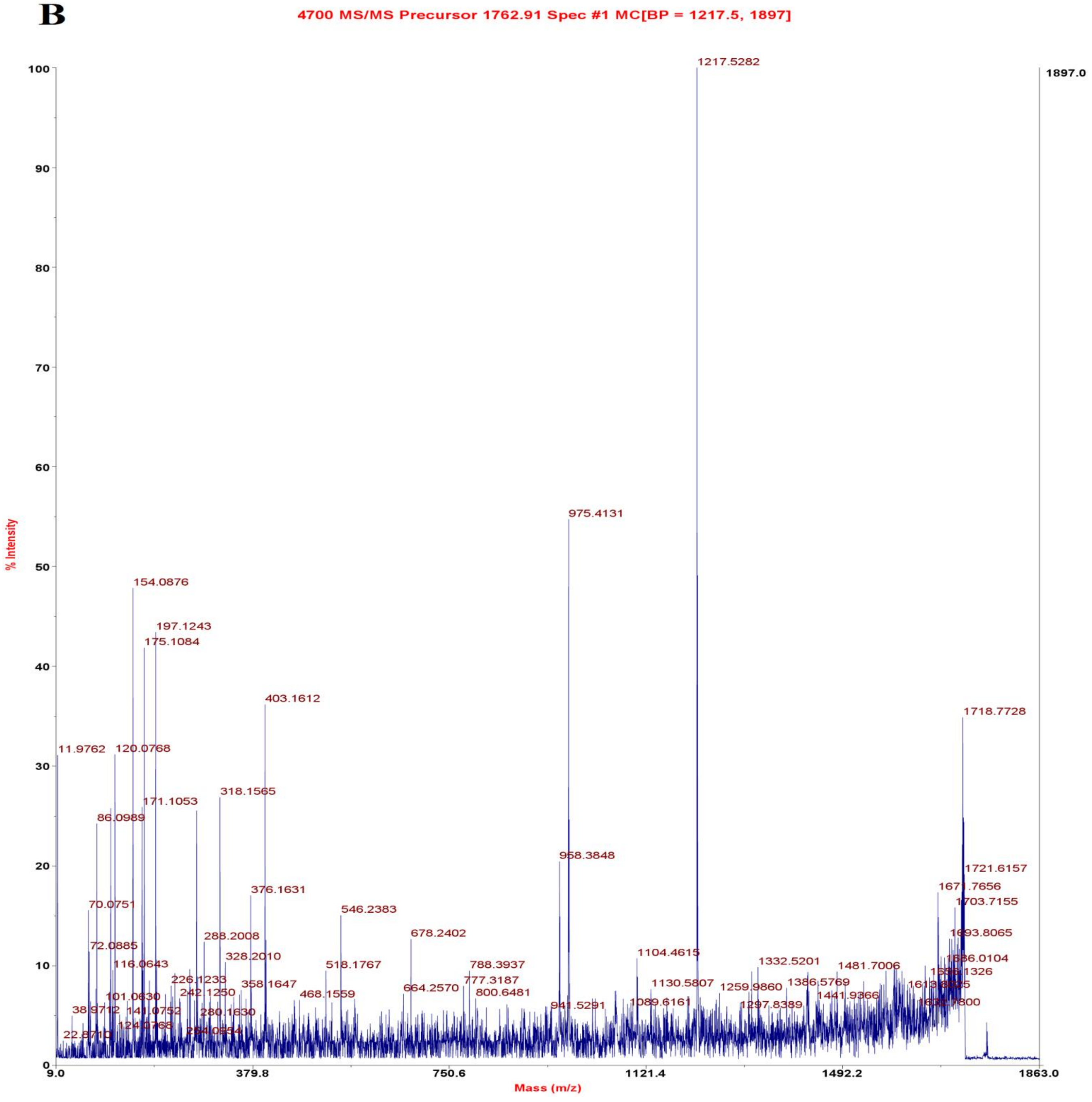

Fig. S7 Mass spectra of spot (1120) in resting cyst

A: Peptide mass fingerprinting of ribosomal protein-serine acetyltransferase (1120) inresting cyst; B: MS/MS spectrum of ribosomal protein-serine acetyltransferase (1120) in resting cyst.
