# Supplementary material for: Proteomic Approach to Reveal the Proteins Associated with Encystment of the Ciliate Euplotes encysticus
Source: PLoS One. 2014 May 16;9(5):e97362. doi: 10.1371/journal.pone.0097362 (PMC4023950; doi:10.1371/journal.pone.0097362)
Supplement: Figure S8 — Mass spectra of spot (1136) in resting cyst. A: Peptide mass fingerprinting of Nop16 domain containing protein (1136) in resting cyst; B1-B6: MS/MS spectrum of Nop16 domain containing protein (1136) in resting cyst. (PDF) [file pone.0097362.s008.pdf]

A

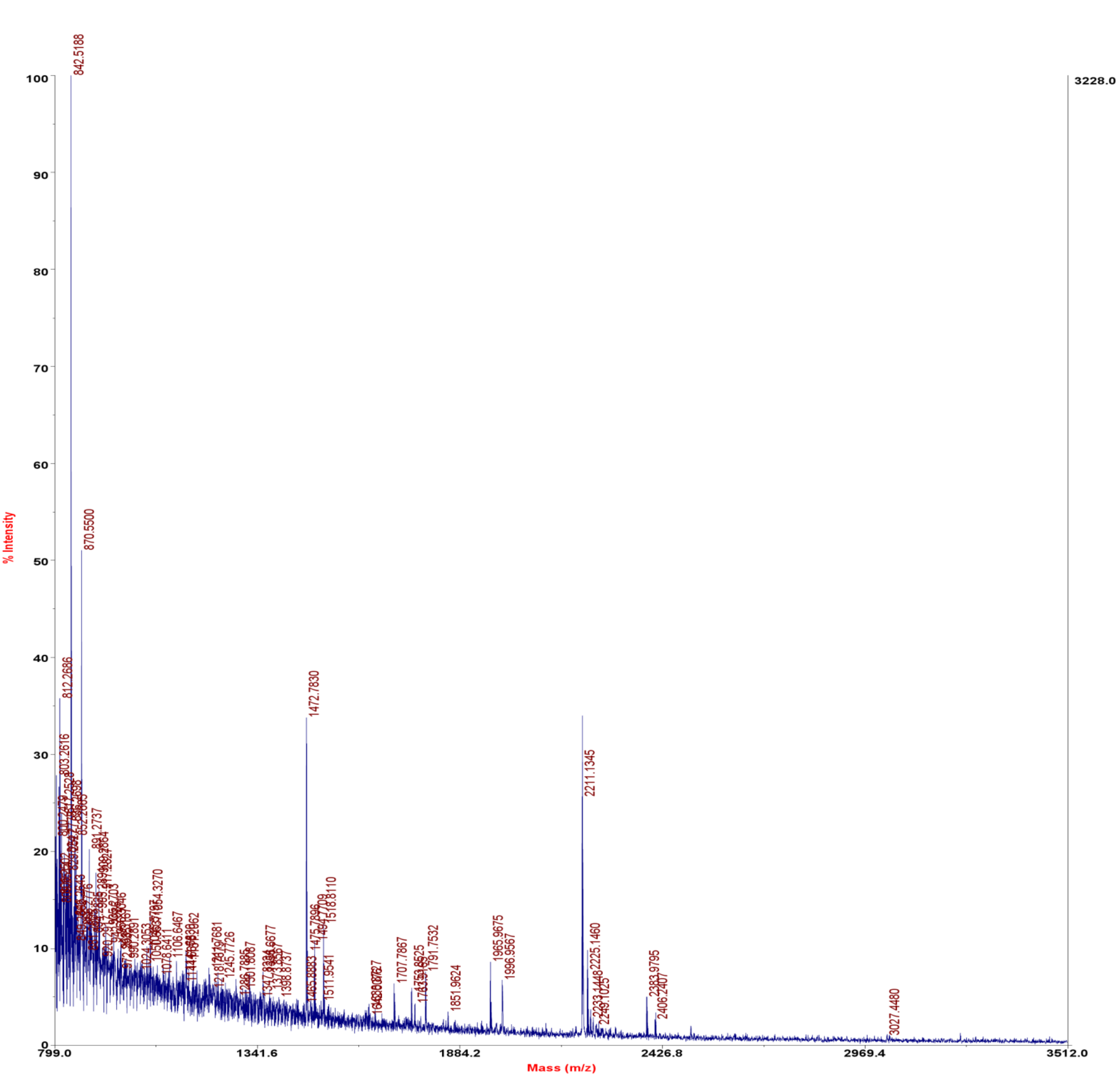
4700 Reflector Spec #1 MC[BP = 842.5, 3228]
% Intensity
Mass (m/z)
3228.0
842.5188
870.5500
812.2686
803.2616
1472.7830
2211.1345
2225.1460
1965.9675
1996.9567
1707.7867
1791.7532
1851.9624
2383.9795
2406.2407
3027.4480
1518.8110
1511.9541
1054.3270
1106.6467
1245.7726
891.2737
799.0
1341.6
1884.2
2426.8
2969.4
3512.0

B1

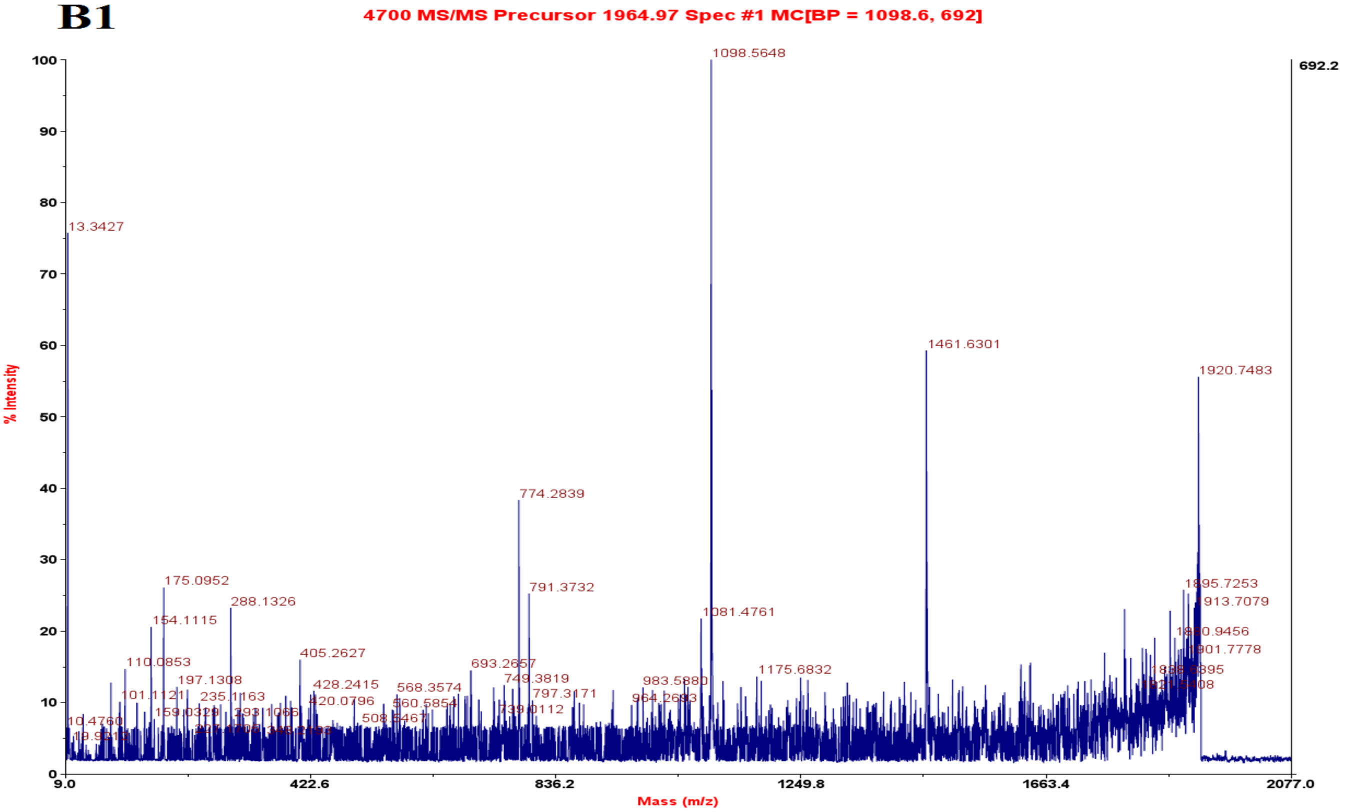

4700 MS/MS Precursor 1964.97 Spec #1 MC[BP = 1098.6, 692]
% Intensity
Mass (m/z)
692.2
1098.5648
13.3427
1461.6301
1920.7483
774.2839
791.3732
175.0952
288.1326
154.1115
1081.4761
1895.7253
1913.7079
405.2627
110.0853
693.2657
197.1308
749.3819
983.5880
1175.6832
428.2415
568.3574
797.3171
235.1163
420.0796
560.5854
964.2693
9.0
422.6
836.2
1249.8
1663.4
2077.0

B2

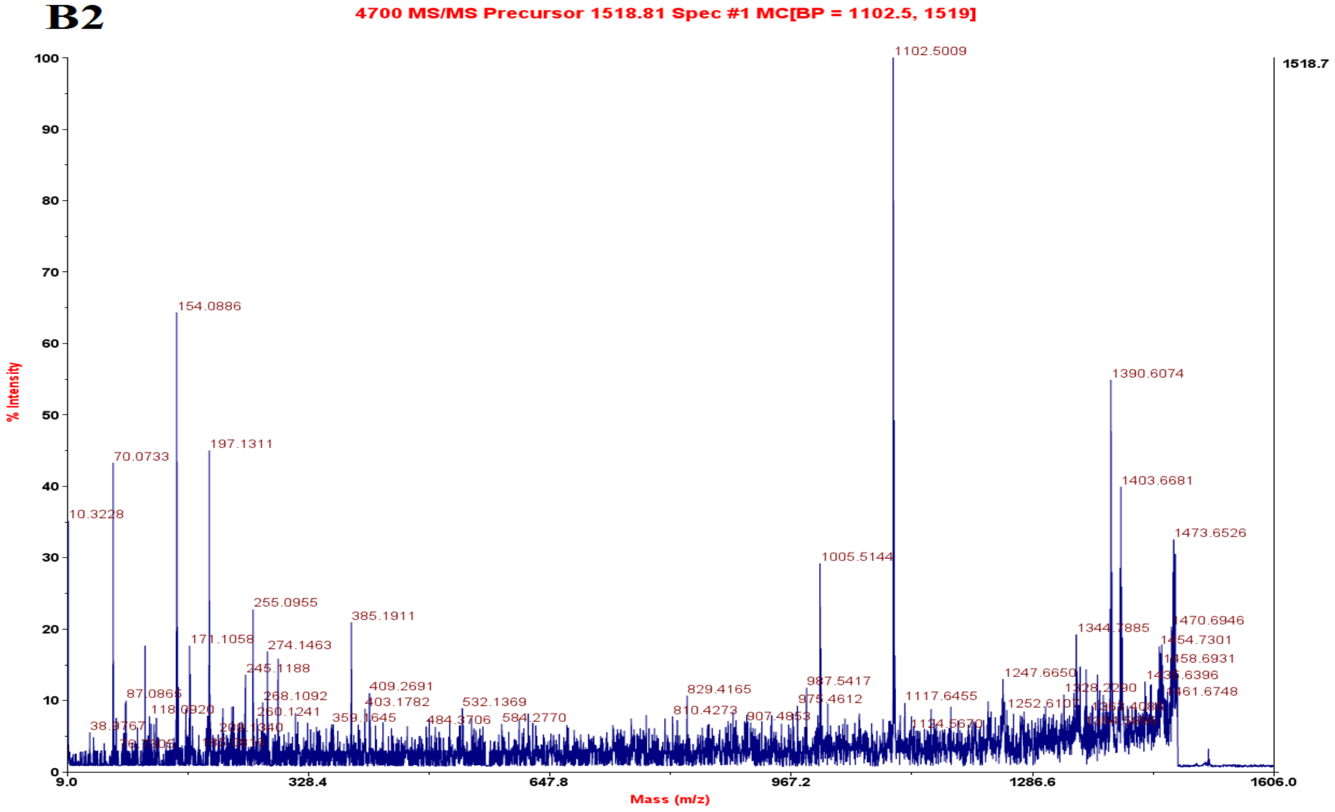

4700 MS/MS Precursor 1518.81 Spec #1 MC[BP = 1102.5, 1519]
% Intensity
Mass (m/z)
1518.7
1102.5009
154.0886
1390.6074
197.1311
70.0733
1403.6681
10.3228
1473.6526
1005.5144
255.0955
385.1911
1344.7885
1470.6946
171.1058
274.1463
1454.7301
1458.6931
245.1188
1247.6650
987.5417
409.2691
829.4165
87.0865
268.1092
1117.6455
975.4612
403.1782
532.1369
118.0920
260.1241
810.4273
1252.6100
1461.6748
907.4853
359.1645
484.3706
584.2770
38.9767
9.0
328.4
647.8
967.2
1286.6
1606.0

B3

4700 MS/MS Precursor 1493.75 Spec #1 MC[BP = 154.1, 1078]

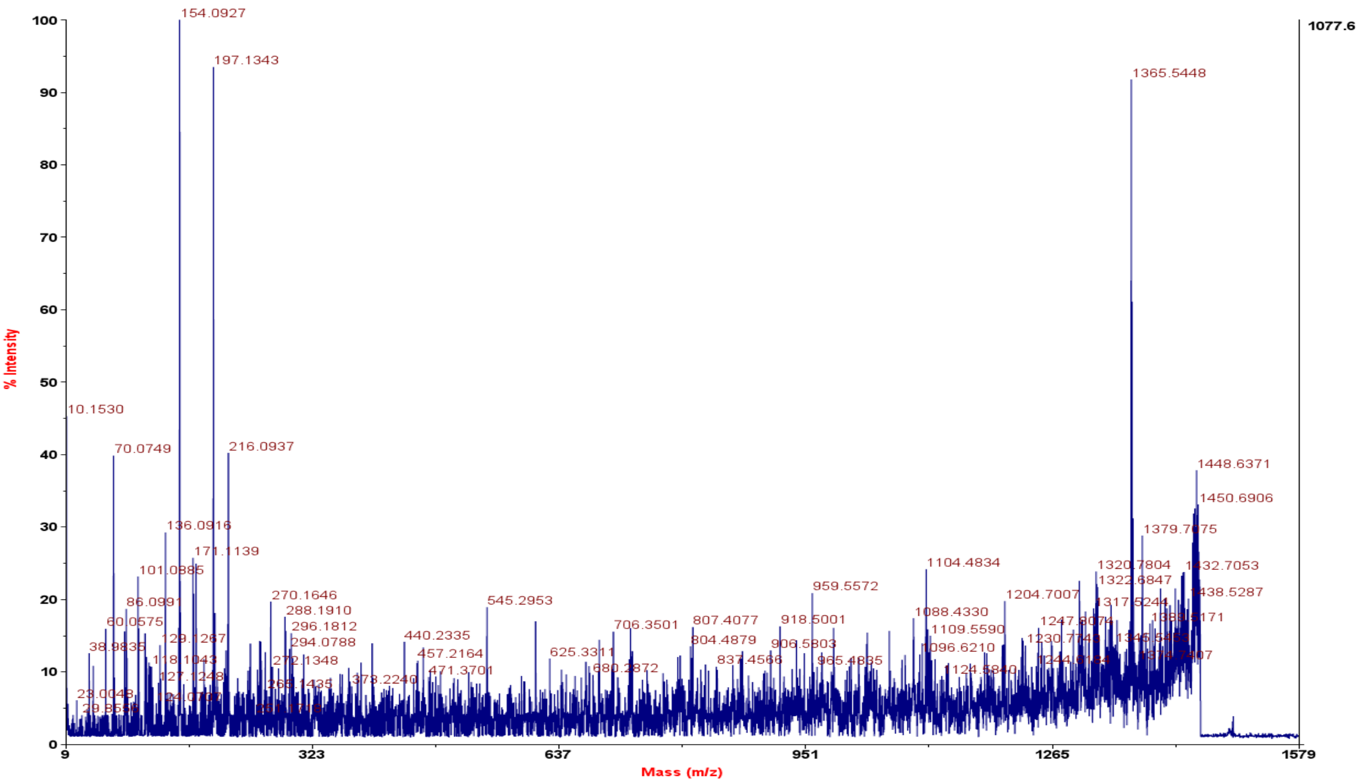

B4

4700 MS/MS Precursor 1472.78 Spec #1 MC[BP = 457.2, 1200]

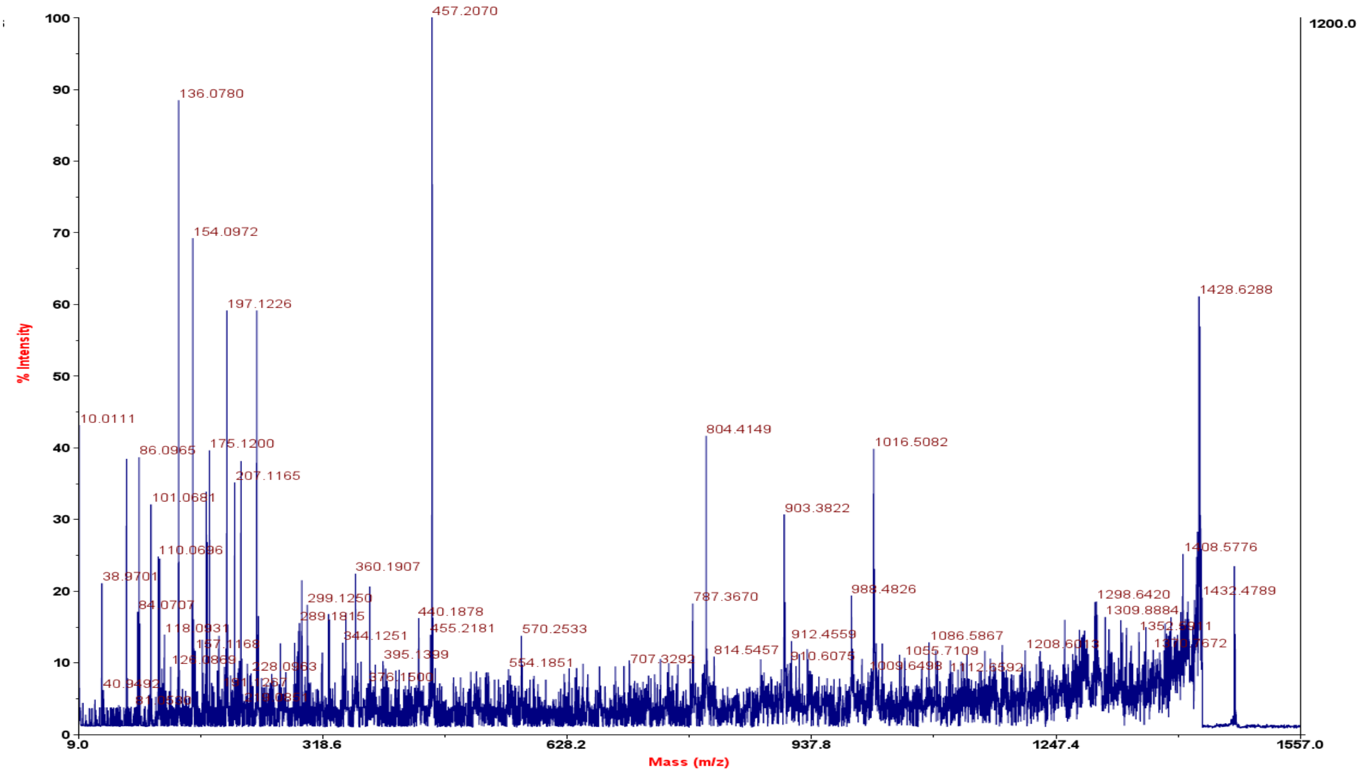

B5

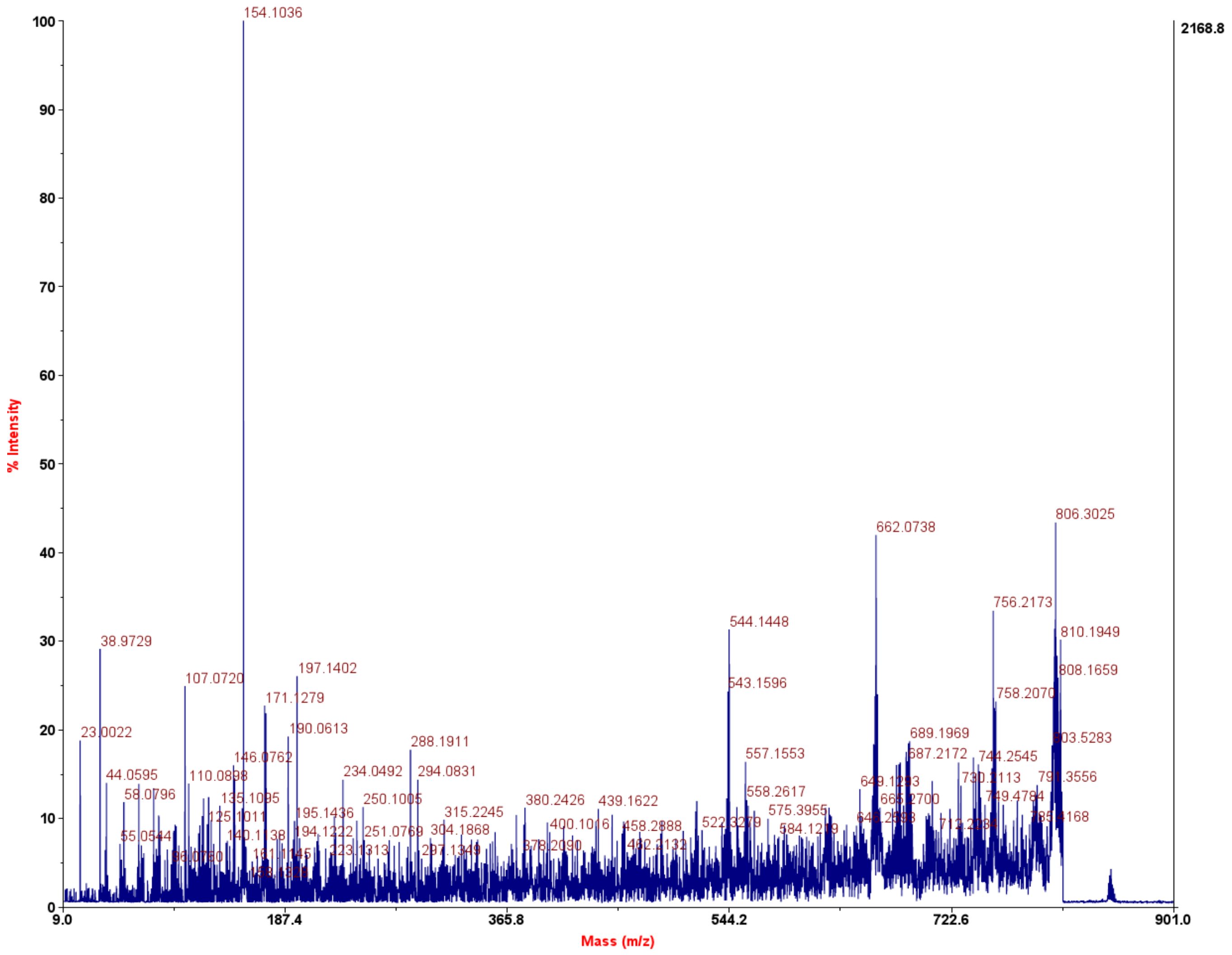

4700 MS/MS Precursor 852.266 Spec #1 MC[BP = 154.1, 2169]

B6

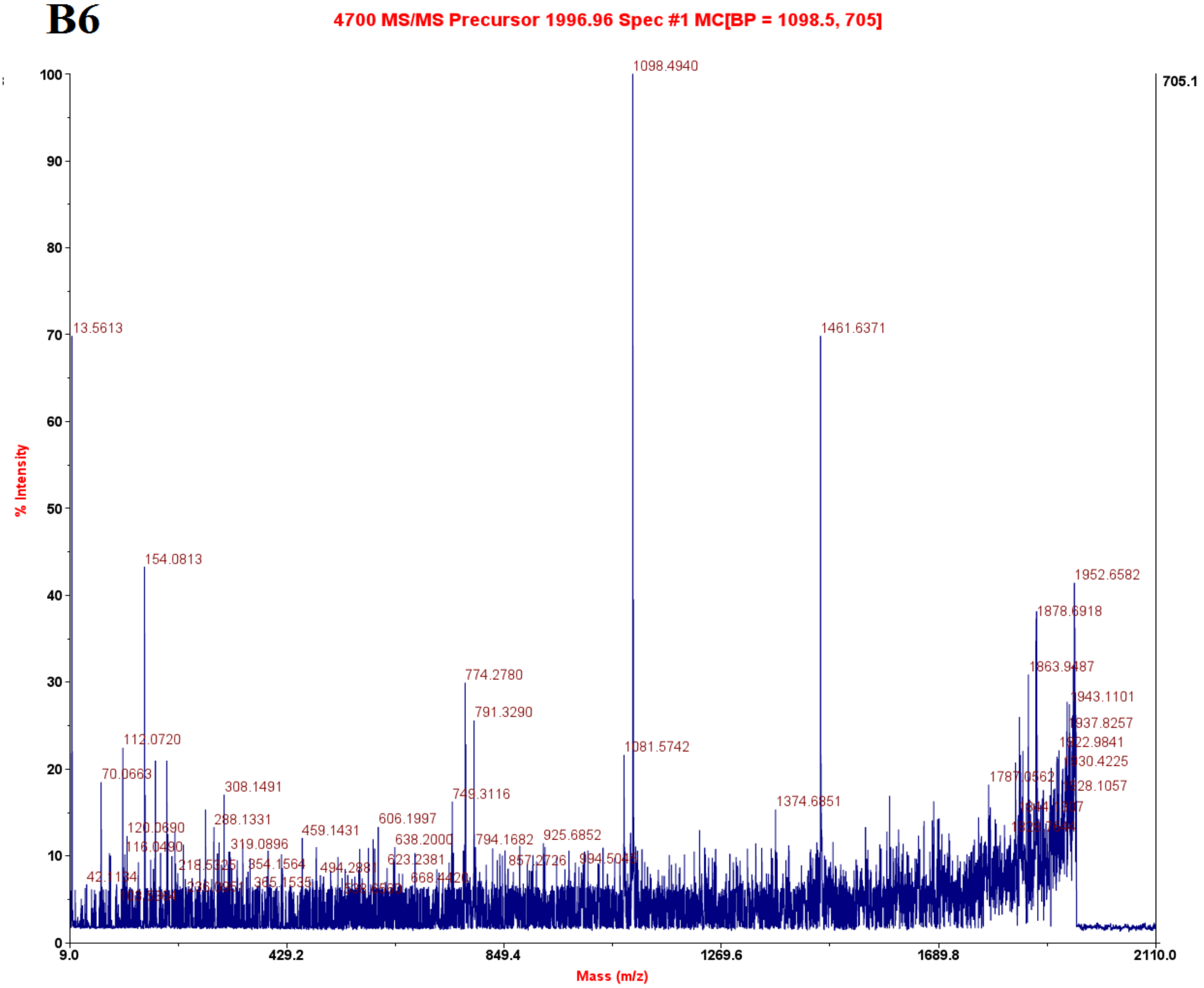

Fig. S8 Mass spectra of spot (1136) in resting cyst

A: Peptide mass fingerprinting of Nop16 domain containing protein (1136) in resting cyst; B1-B6: MS/MS spectrum of Nop16 domain containing protein (1136) in resting cyst.
