# Supplementary material for: Proteomic Approach to Reveal the Proteins Associated with Encystment of the Ciliate Euplotes encysticus
Source: PLoS One. 2014 May 16;9(5):e97362. doi: 10.1371/journal.pone.0097362 (PMC4023950; doi:10.1371/journal.pone.0097362)
Supplement: Figure S9 — Mass spectra of spot (1137) in resting cyst. A: Peptide mass fingerprinting of spot (1137) in resting cyst; B1-B3: MS/MS spectrum of spot (1137) in resting cyst. (PDF) [file pone.0097362.s009.pdf]

A

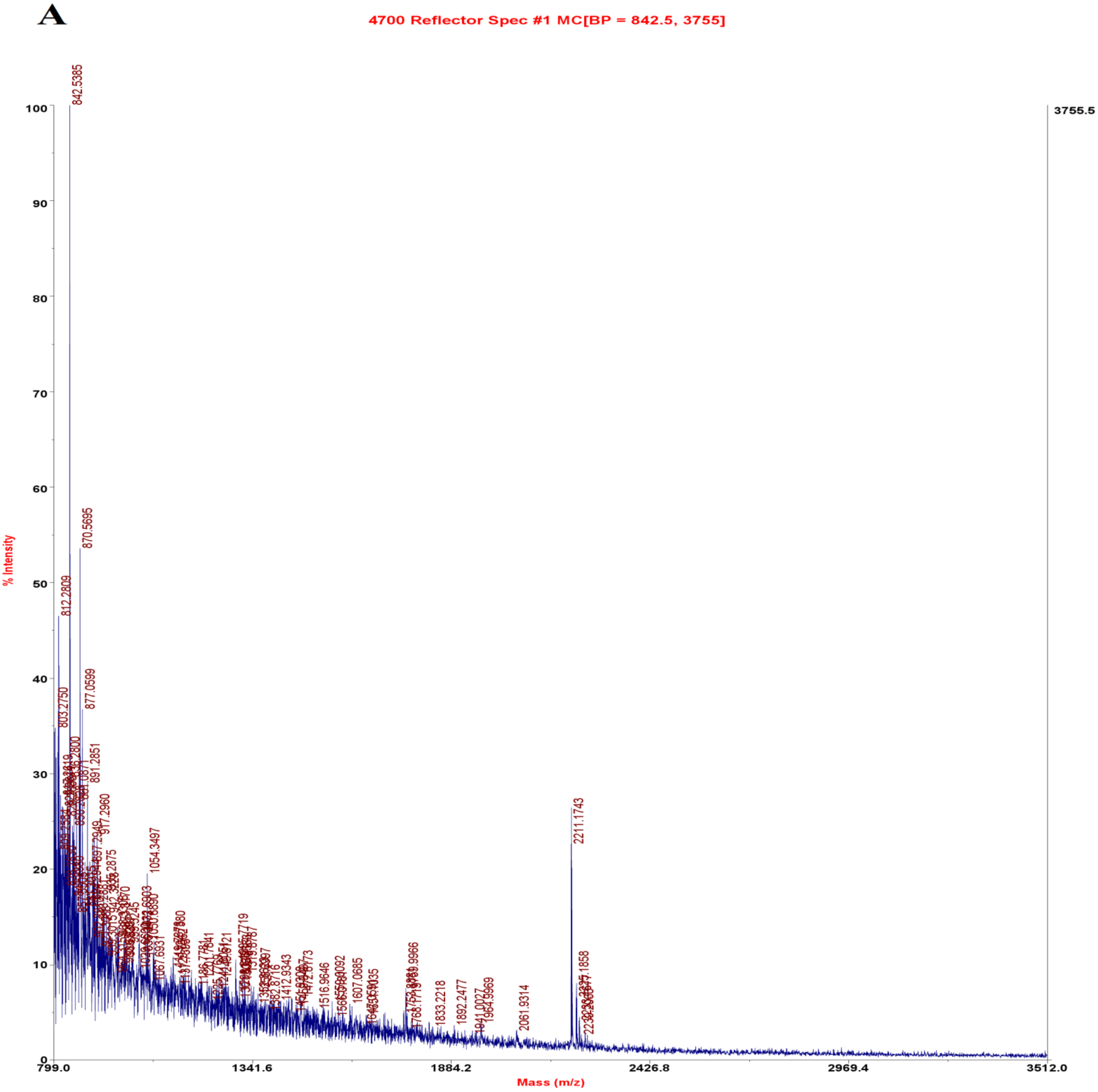
4700 Reflector Spec #1 MC[BP = 842.5, 3755]
% Intensity
Mass (m/z)
3755.5
842.5385
870.5695
812.2809
877.0599
803.2750
891.2851
917.2960
1054.3497
2211.1743
2061.9314
1964.9969
1892.2477
1833.2218
1607.0685
1516.9646
1412.9343
799.0
1341.6
1884.2
2426.8
2969.4
3512.0

B1

4700 MS/MS Precursor 891.285 Spec #1 MC[BP = 154.1, 2825]

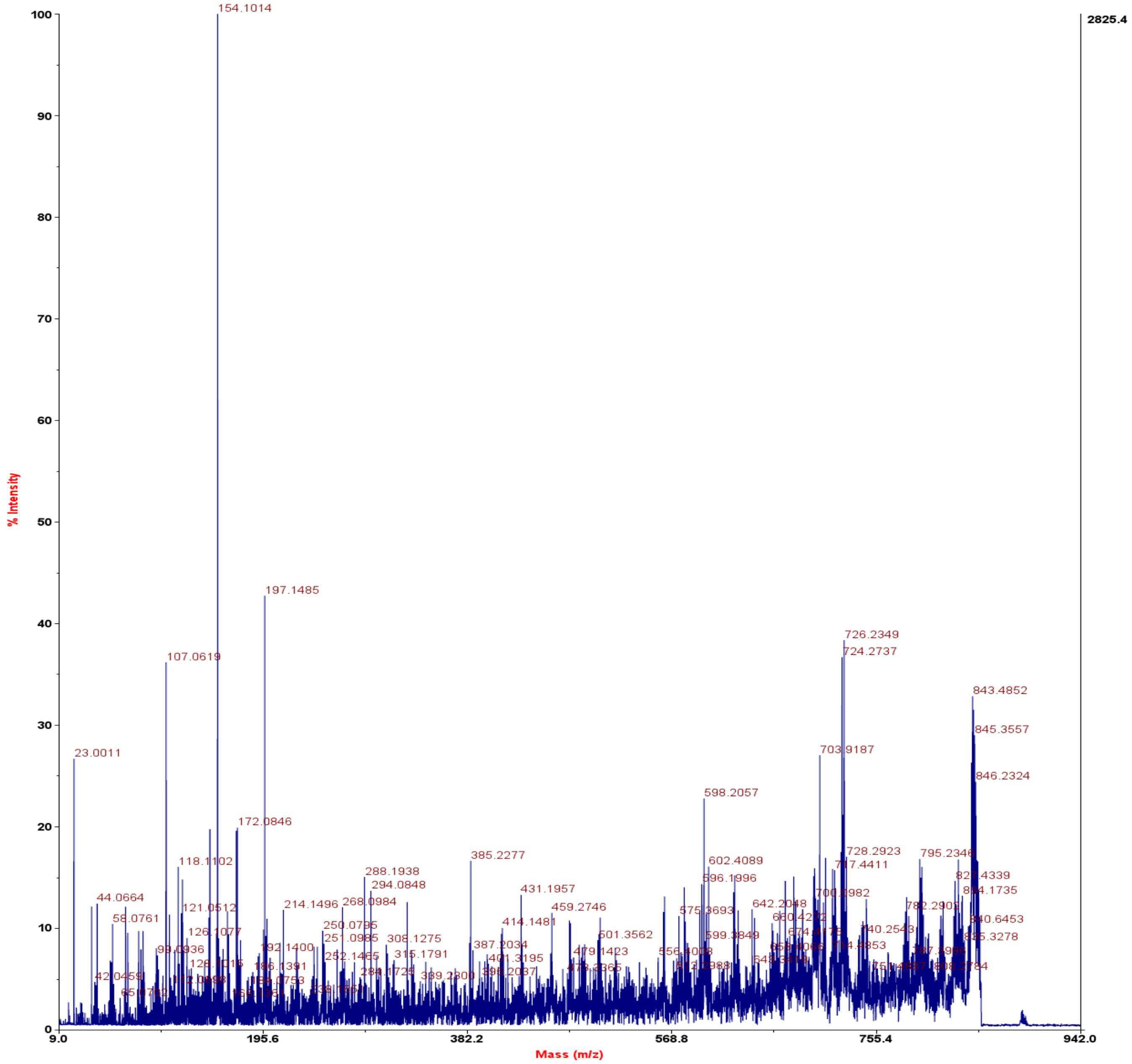

B2

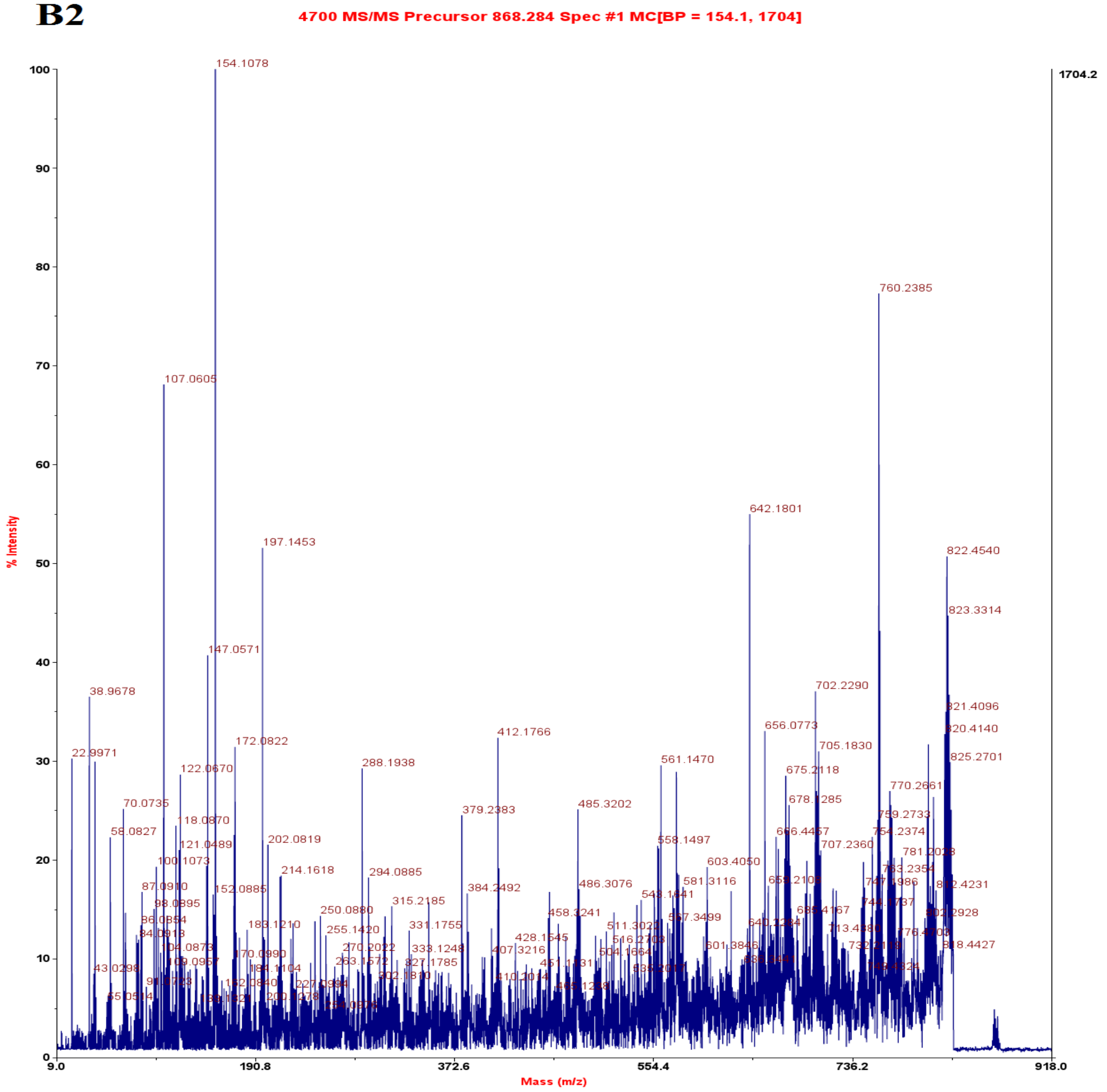
4700 MS/MS Precursor 868.284 Spec #1 MC[BP = 154.1, 1704]
% Intensity
Mass (m/z)
1704.2
100
90
80
70
60
50
40
30
20
10
0
9.0
190.8
372.6
554.4
736.2
918.0
154.1078
760.2385
107.0605
642.1801
197.1453
822.4540
823.3314
147.0571
38.9678
702.2290
821.4096
656.0773
820.4140
412.1766
172.0822
22.9971
705.1830
825.2701
288.1938
561.1470
675.2118
122.0670
770.2661
70.0735
485.3202
379.2383
678.1285
118.0870
759.2733
58.0827
666.4457
754.2374
558.1497
202.0819
121.0489
707.2360
603.4050
781.2028
100.1073
214.1618
294.0885
581.3116
486.3076
384.2492
152.0885
87.0910
315.2185
98.0895
250.0880
458.3241
567.3499
86.0854
331.1755
183.1210
255.1420
84.0913
428.1545
333.1248
270.2022
407.3216
43.0298
170.0990
327.1785
263.1572
410.2014
55.0514

B3

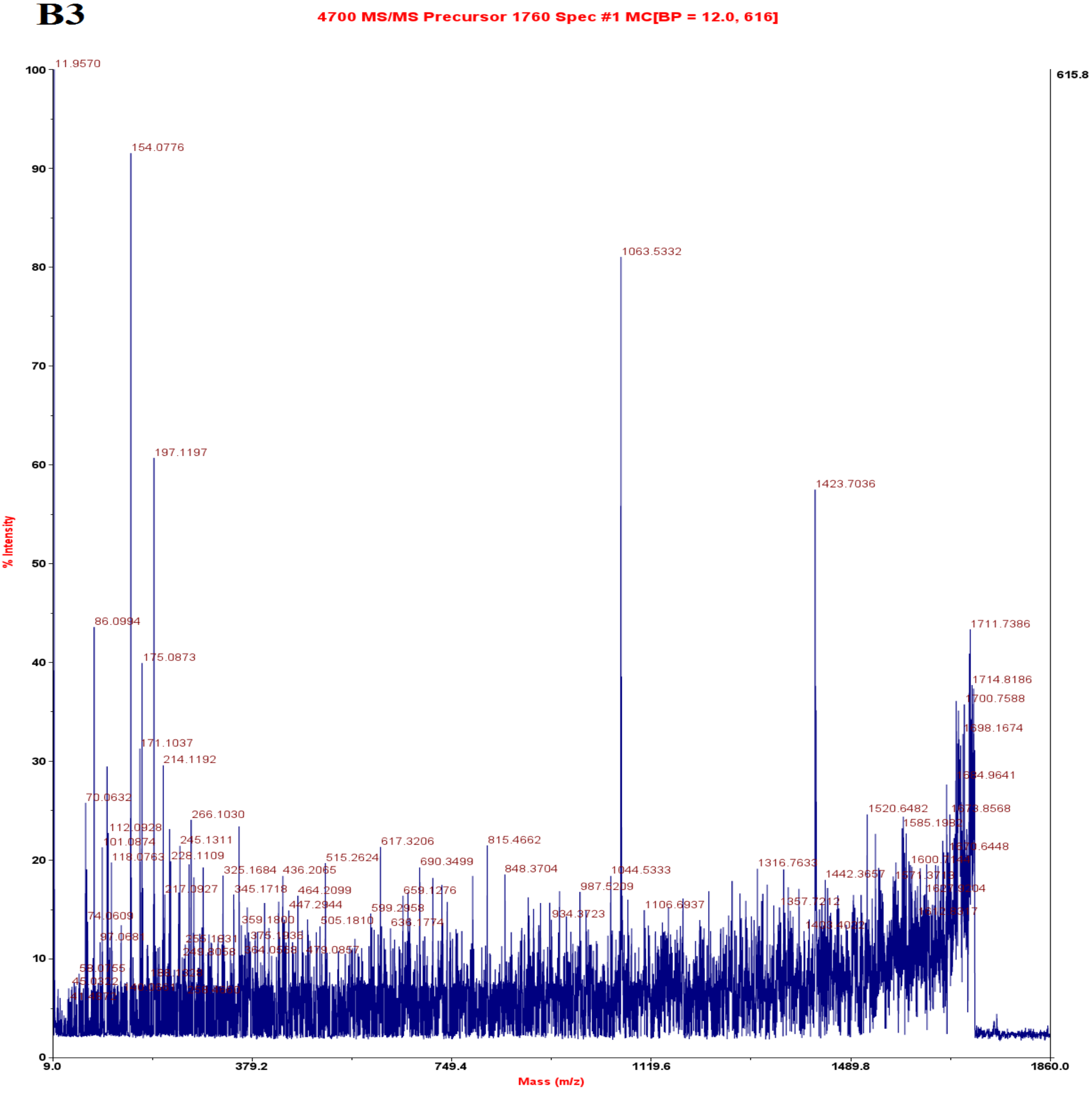

Fig. S9 Mass spectra of spot (1137) in resting cyst

A: Peptide mass fingerprinting of spot (1137) in resting cyst; B1-B3: MS/MS spectrum of spot (1137) in resting cyst.
