# Supplementary material for: Proteomic Approach to Reveal the Proteins Associated with Encystment of the Ciliate Euplotes encysticus
Source: PLoS One. 2014 May 16;9(5):e97362. doi: 10.1371/journal.pone.0097362 (PMC4023950; doi:10.1371/journal.pone.0097362)
Supplement: Figure S10 — Mass spectra of spot (1148) in resting cyst. A: Peptide mass fingerprinting of keratin (1148) in resting cyst; B1-B12: MS/MS spectrum of keratin (1148) in resting cyst. (PDF) [file pone.0097362.s010.pdf]

A

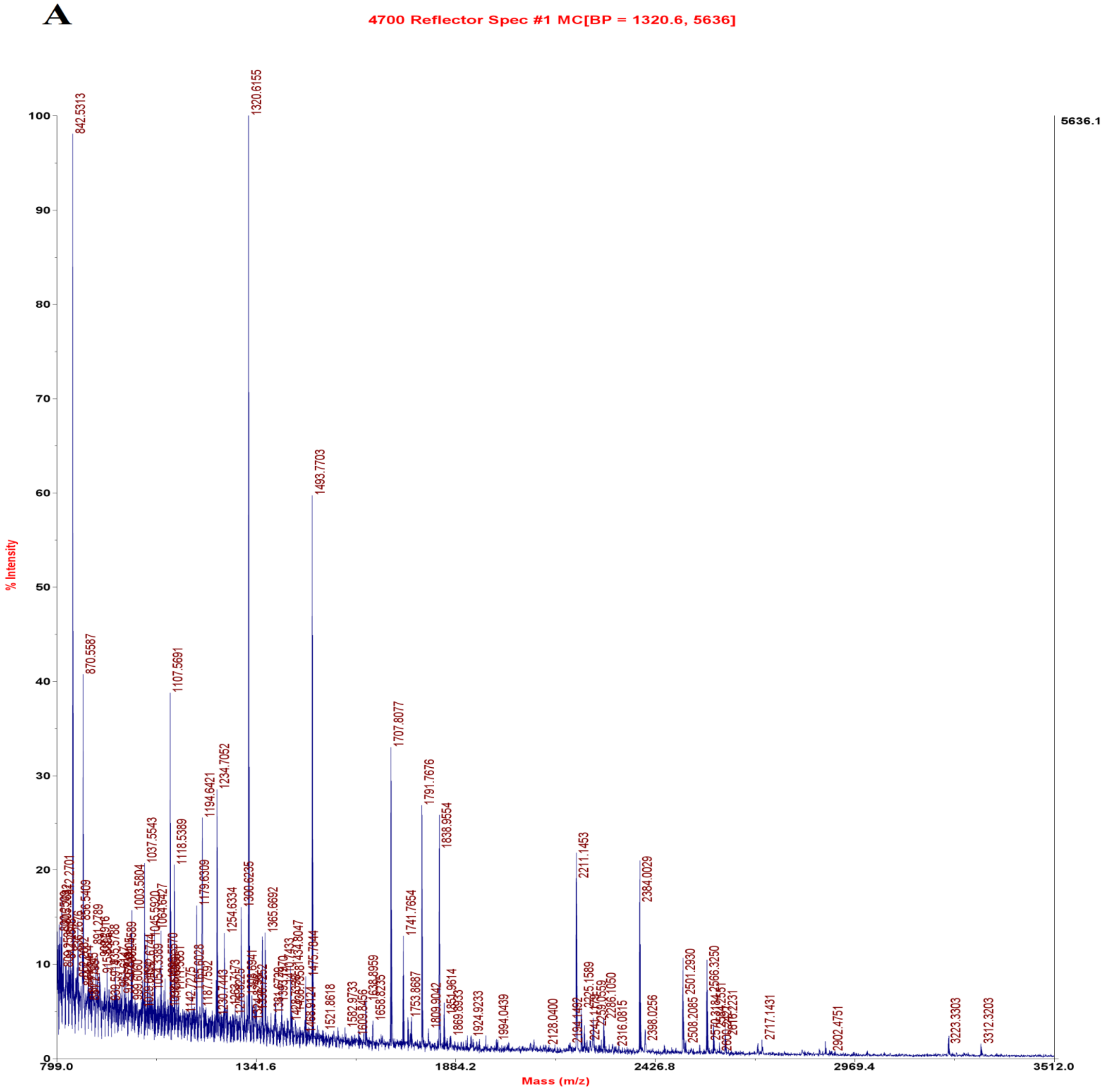
4700 Reflector Spec #1 MC[BP = 1320.6, 5636]
% Intensity
Mass (m/z)
5636.1
842.5313
870.5587
1107.5691
1118.5389
1194.6421
1234.7052
1320.6155
1493.7703
1707.8077
1741.7654
1791.7676
1838.9554
2211.1453
2384.0029
2501.2930
2566.3250
2717.1431
2902.4751
3223.3303
3312.3203

B1

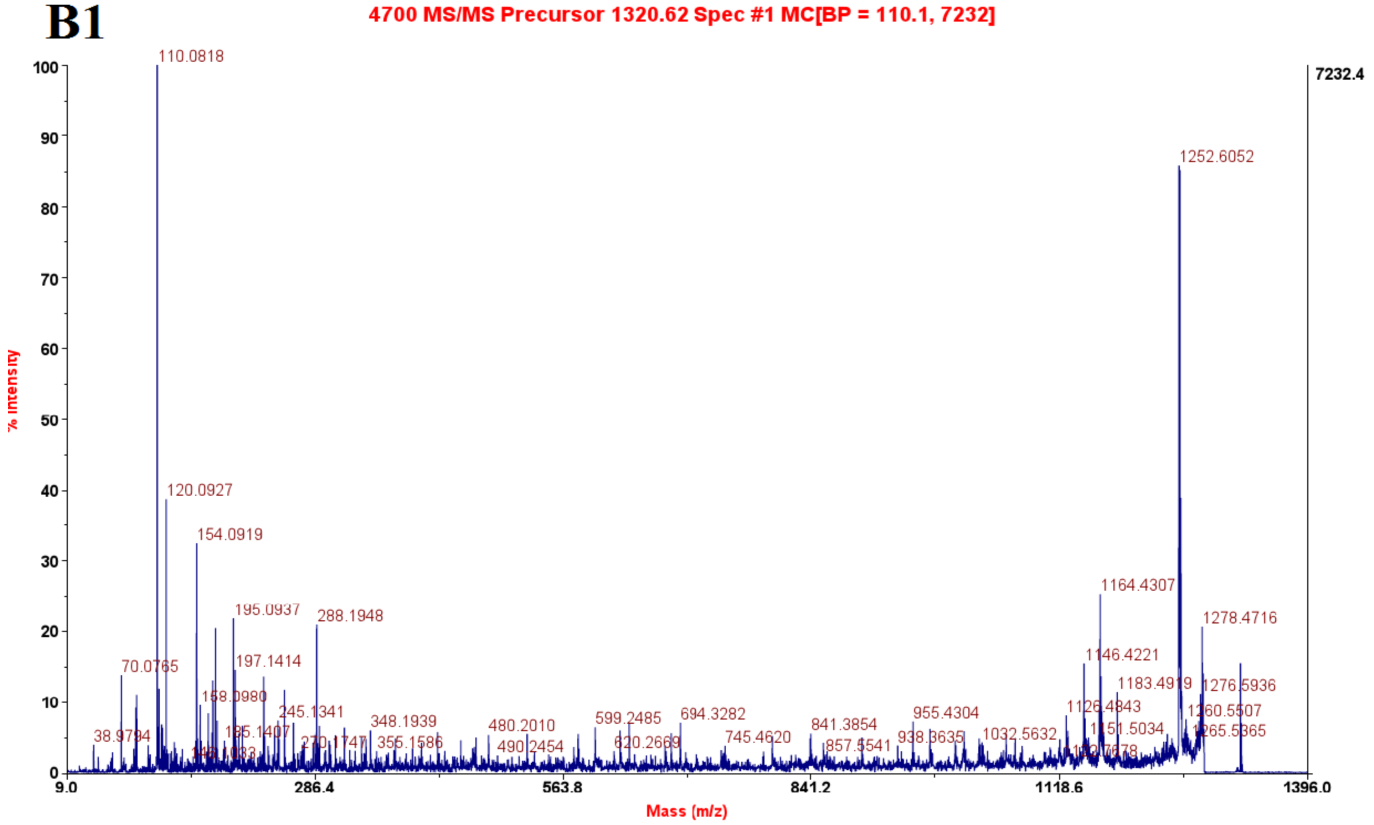
4700 MS/MS Precursor 1320.62 Spec #1 MC[BP = 110.1, 7232]
7232.4
% Intensity
Mass (m/z)
110.0818
1252.6052
120.0927
154.0919
195.0937
288.1948
197.1414
70.0765
1164.4307
1278.4716
1146.4221
1183.4919
1276.5936
1126.4843
1260.5507
1151.5034
1265.5365
158.0980
245.1341
38.9794
348.1939
480.2010
599.2485
694.3282
841.3854
955.4304
745.4620
1032.5632
355.1586
490.2454
620.2669
857.5541
938.3635

B2

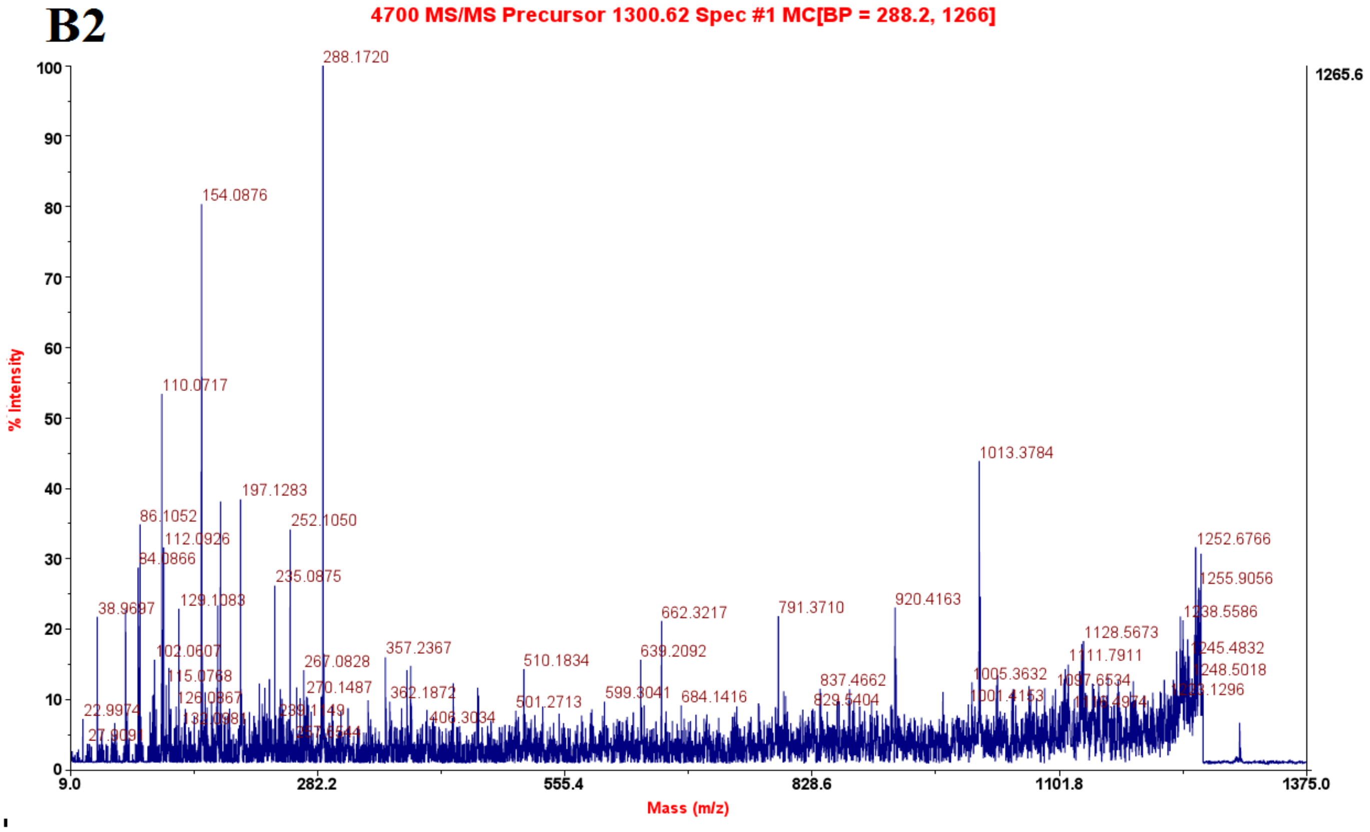
4700 MS/MS Precursor 1300.62 Spec #1 MC[BP = 288.2, 1266]
1265.6
% Intensity
Mass (m/z)
288.1720
154.0876
110.0717
197.1283
86.1052
252.1050
112.0926
84.0866
235.0875
38.9697
129.1083
1013.3784
1252.6766
1255.9056
1238.5586
1128.5673
1111.7911
1245.4832
1248.5018
920.4163
791.3710
662.3217
639.2092
510.1834
357.2367
267.0828
270.1487
102.0607
115.0768
22.9974
27.9091
362.1872
406.3034
501.2713
599.3041
684.1416
837.4662
829.5404
1005.3632
1001.4153
1097.6534

B3

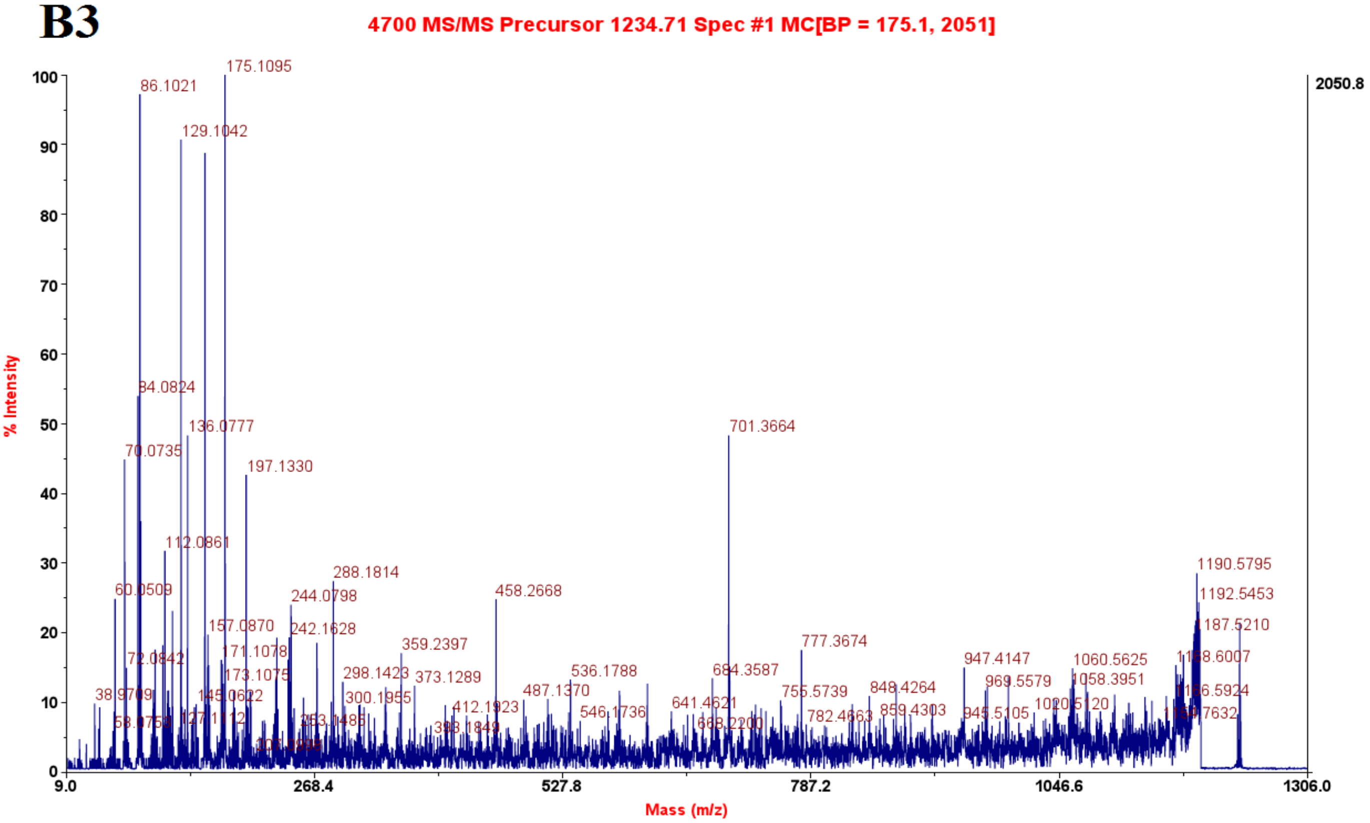
4700 MS/MS Precursor 1234.71 Spec #1 MC[BP = 175.1, 2051]
% Intensity
Mass (m/z)
2050.8
175.1095
86.1021
129.1042
84.0824
136.0777
70.0735
197.1330
112.0861
701.3664
288.1814
244.0798
458.2668
60.0509
157.0870
242.1628
359.2397
777.3674
1190.5795
1192.5453
187.5210
947.4147
1060.5625
969.5579
1058.3951
298.1423
373.1289
536.1788
684.3587
755.5739
848.4264
487.1370
300.1955
641.4621
412.1923
546.1736
859.4303
782.4663
945.5105

B4

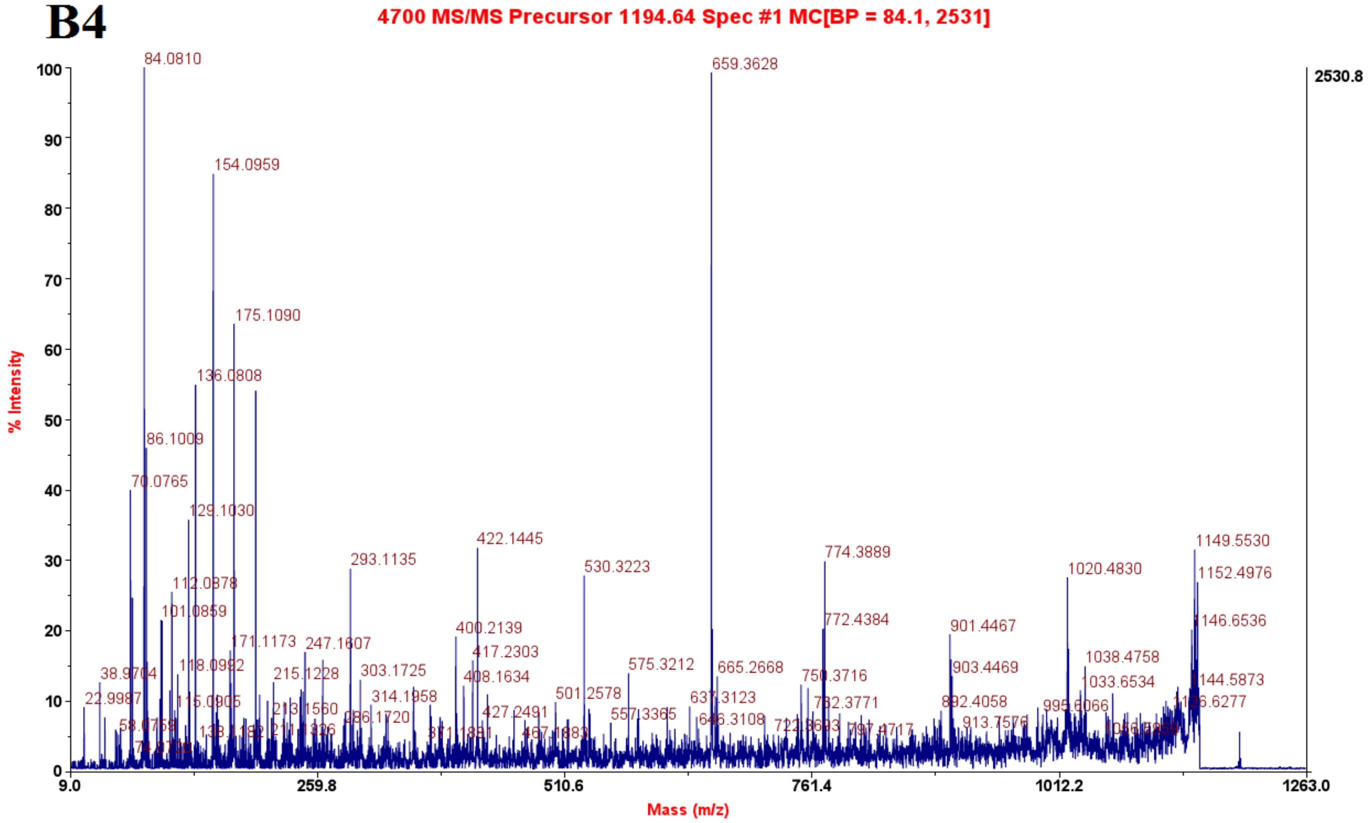
4700 MS/MS Precursor 1194.64 Spec #1 MC[BP = 84.1, 2531]
% Intensity
Mass (m/z)
2530.8
84.0810
659.3628
154.0959
175.1090
136.0808
86.1009
70.0765
129.1030
422.1445
293.1135
530.3223
774.3889
1149.5530
1152.4976
1020.4830
112.0878
101.0859
400.2139
772.4384
901.4467
1146.6536
417.2303
171.1173
247.1607
575.3212
665.2668
903.4469
1038.4758
38.9704
215.1228
303.1725
408.1634
750.3716
1033.6534
144.5873
22.9987
501.2578
314.1958
637.3123
892.4058
427.2491
557.3365
913.7576

B5

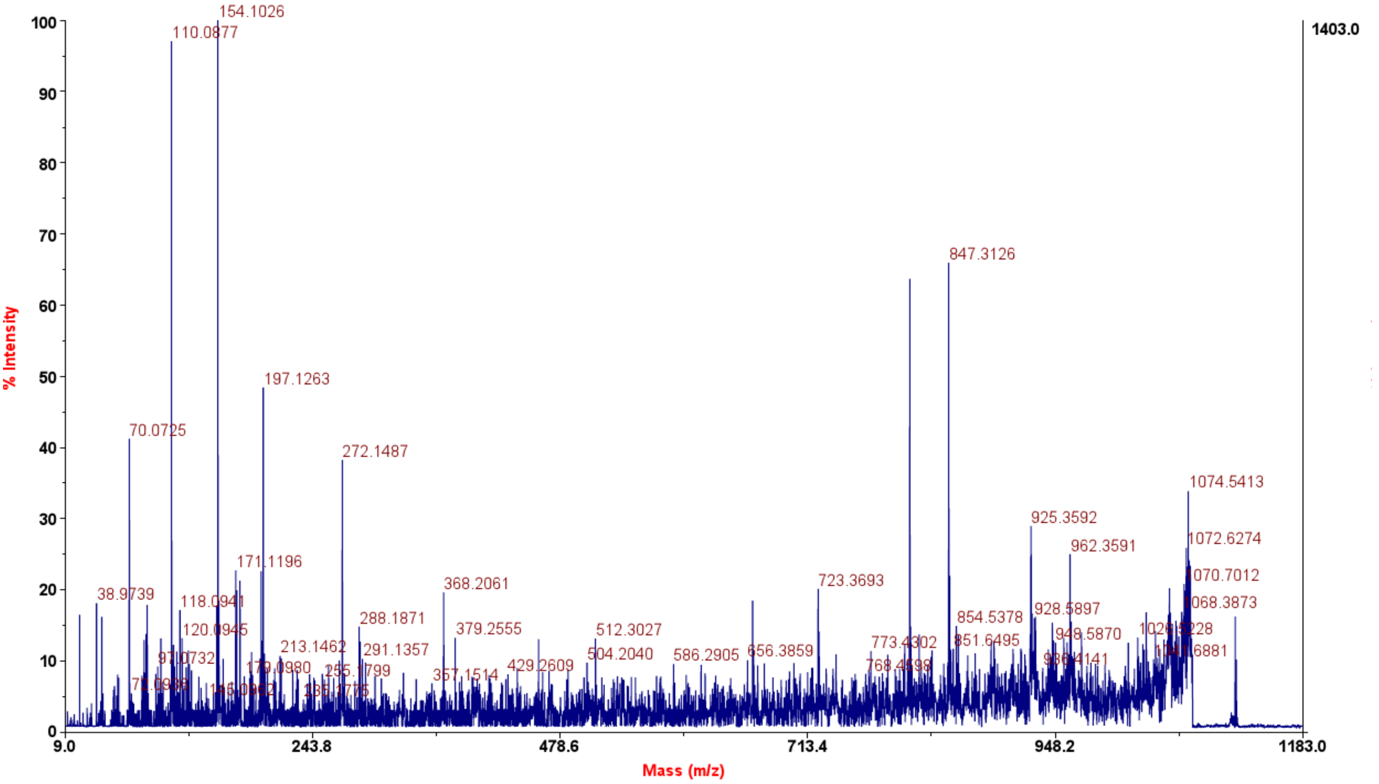
4700 MS/MS Precursor 1118.54 Spec #1 MC[BP = 154.1, 1403]
% Intensity
Mass (m/z)
1403.0
154.1026
110.0877
847.3126
197.1263
70.0725
272.1487
1074.5413
925.3592
962.3591
1072.6274
171.1196
368.2061
723.3693
38.9739
118.0941
288.1871
854.5378
120.0945
379.2555
512.3027
656.3859
773.4302
851.6495
213.1462
291.1357
504.2040
586.2905
429.2609
768.4498

B6

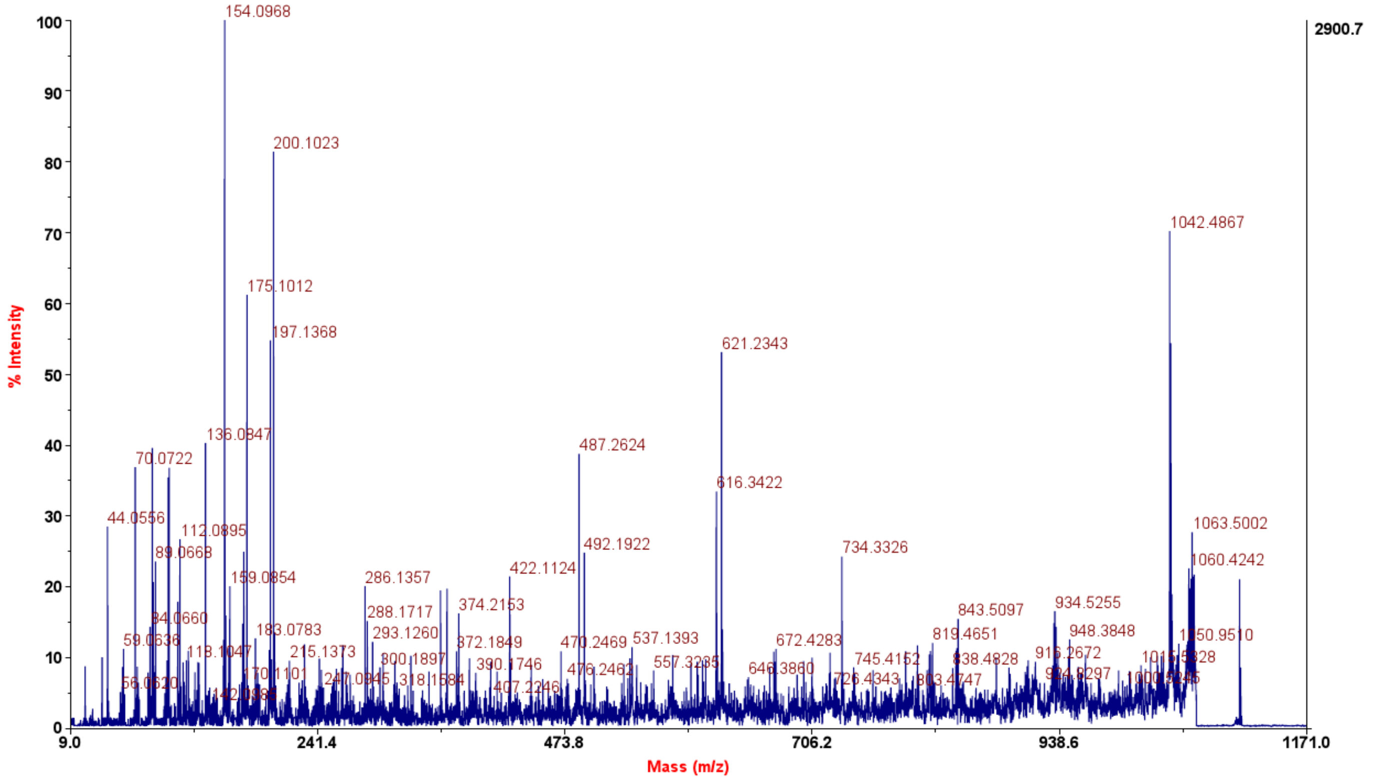
4700 MS/MS Precursor 1107.57 Spec #1 MC[BP = 154.1, 2901]
% Intensity
Mass (m/z)
2900.7
154.0968
200.1023
1042.4867
175.1012
197.1368
621.2343
136.0847
70.0722
487.2624
616.3422
44.0556
112.0895
1063.5002
89.0668
492.1922
734.3326
1060.4242
159.0854
286.1357
422.1124
374.2153
934.5255
288.1717
843.5097
84.0660
183.0783
293.1260
372.1849
948.3848
59.0636
819.4651
537.1393
470.2469
672.4283
1050.9510
118.1047
215.1373
300.1897
390.1746
745.4152
838.4828
557.3235
476.2462
646.3860
726.4343
407.2246

B7

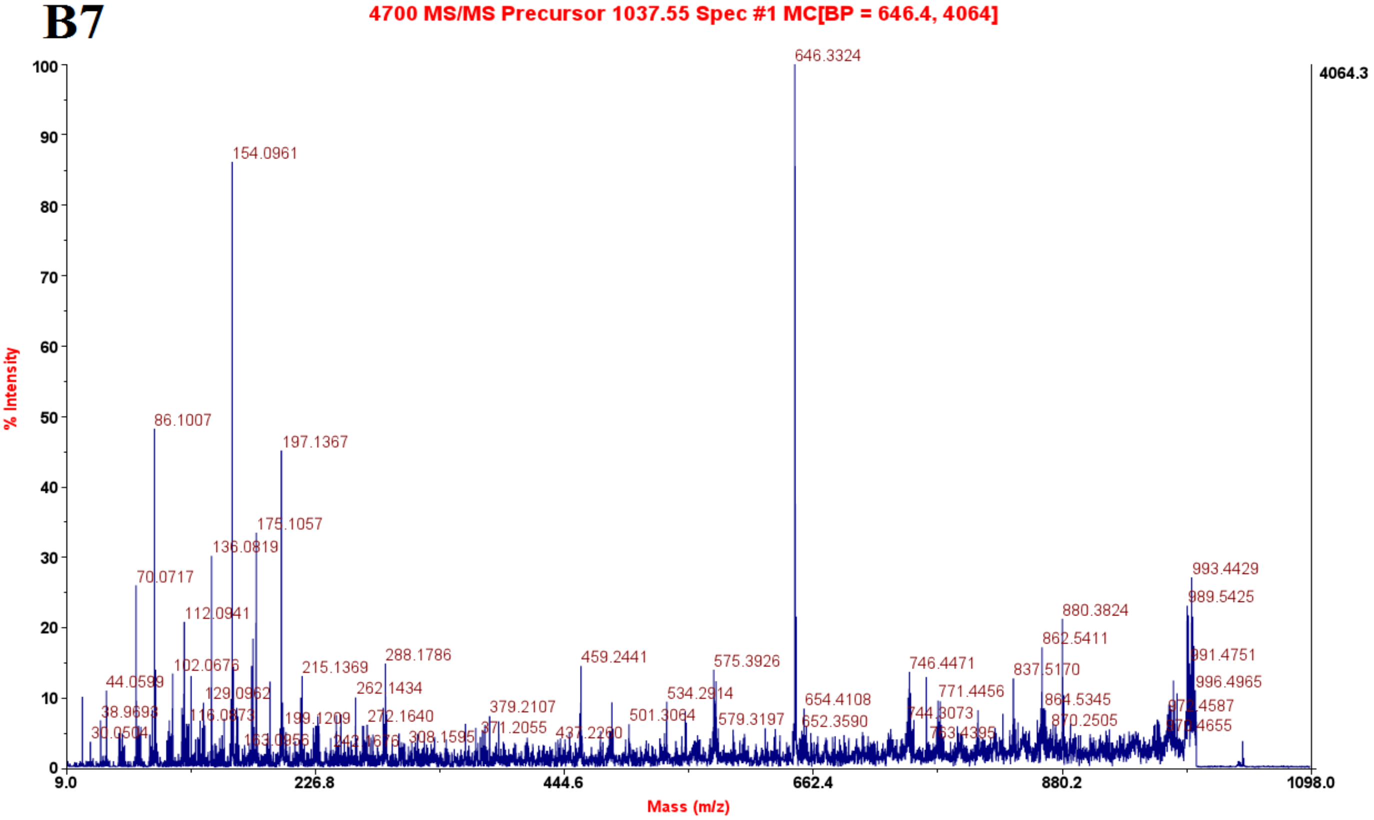
4700 MS/MS Precursor 1037.55 Spec #1 MC[BP = 646.4, 4064]
% Intensity
Mass (m/z)
4064.3
646.3324
154.0961
86.1007
197.1367
175.1057
136.0819
70.0717
112.0941
102.0676
44.0599
129.0962
38.9698
116.0873
30.0504
215.1369
288.1786
262.1434
272.1640
199.1209
379.2107
371.2055
308.1595
459.2441
437.2260
575.3926
534.2914
501.3064
579.3197
654.4108
652.3590
746.4471
771.4456
744.3073
763.4395
837.5170
862.5411
880.3824
864.5345
870.2505
993.4429
989.5425
991.4751
996.4965
9.0
226.8
444.6
662.4
880.2
1098.0

B8

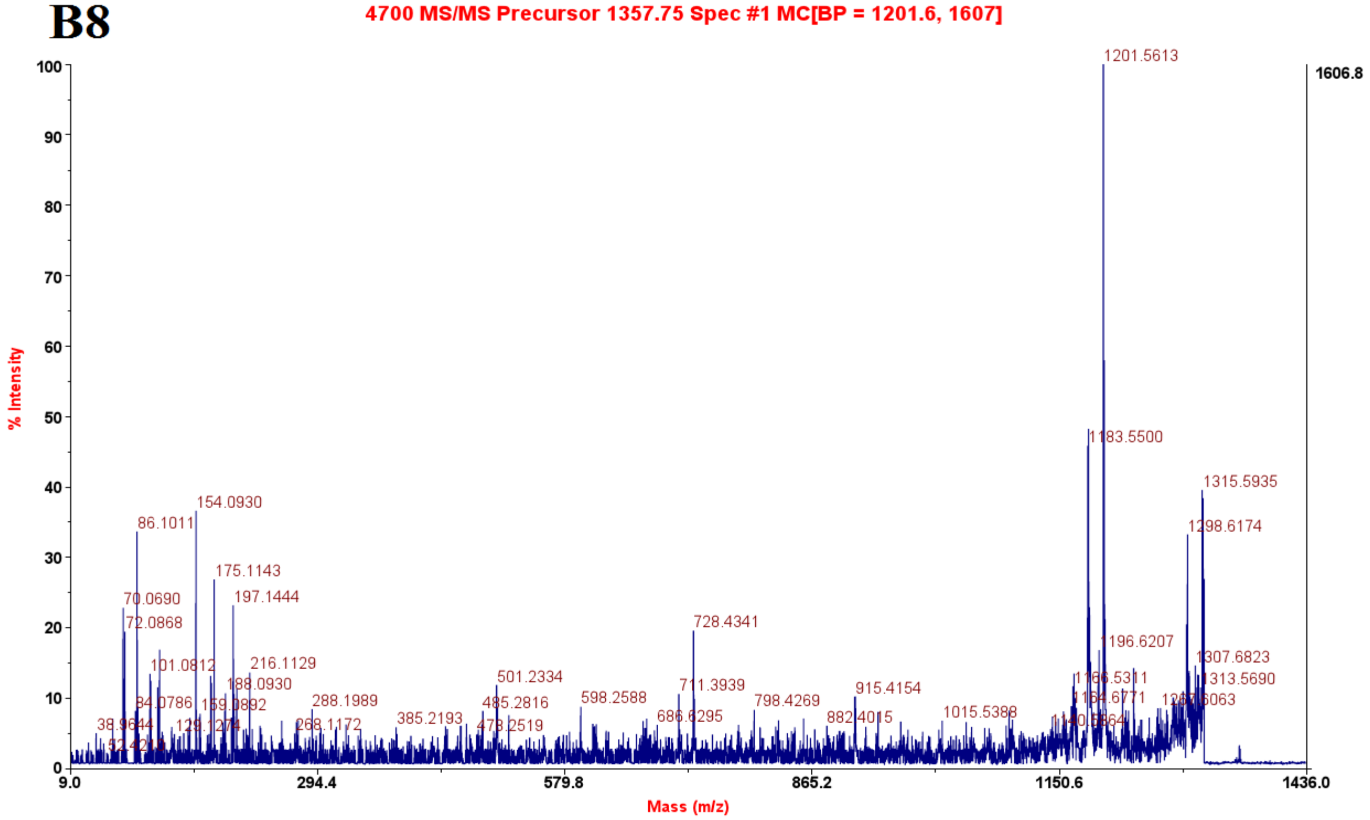
4700 MS/MS Precursor 1357.75 Spec #1 MC[BP = 1201.6, 1607]
% Intensity
Mass (m/z)
1606.8
1201.5613
1183.5500
1315.5935
1298.6174
154.0930
86.1011
175.1143
197.1444
70.0690
72.0868
101.0812
216.1129
188.0930
84.0786
159.0892
38.9644
288.1989
268.1172
385.2193
501.2334
485.2816
473.2519
598.2588
728.4341
711.3939
686.6295
798.4269
915.4154
882.4015
1015.5388
1196.6207
1307.6823
1313.5690
9.0
294.4
579.8
865.2
1150.6
1436.0

B9

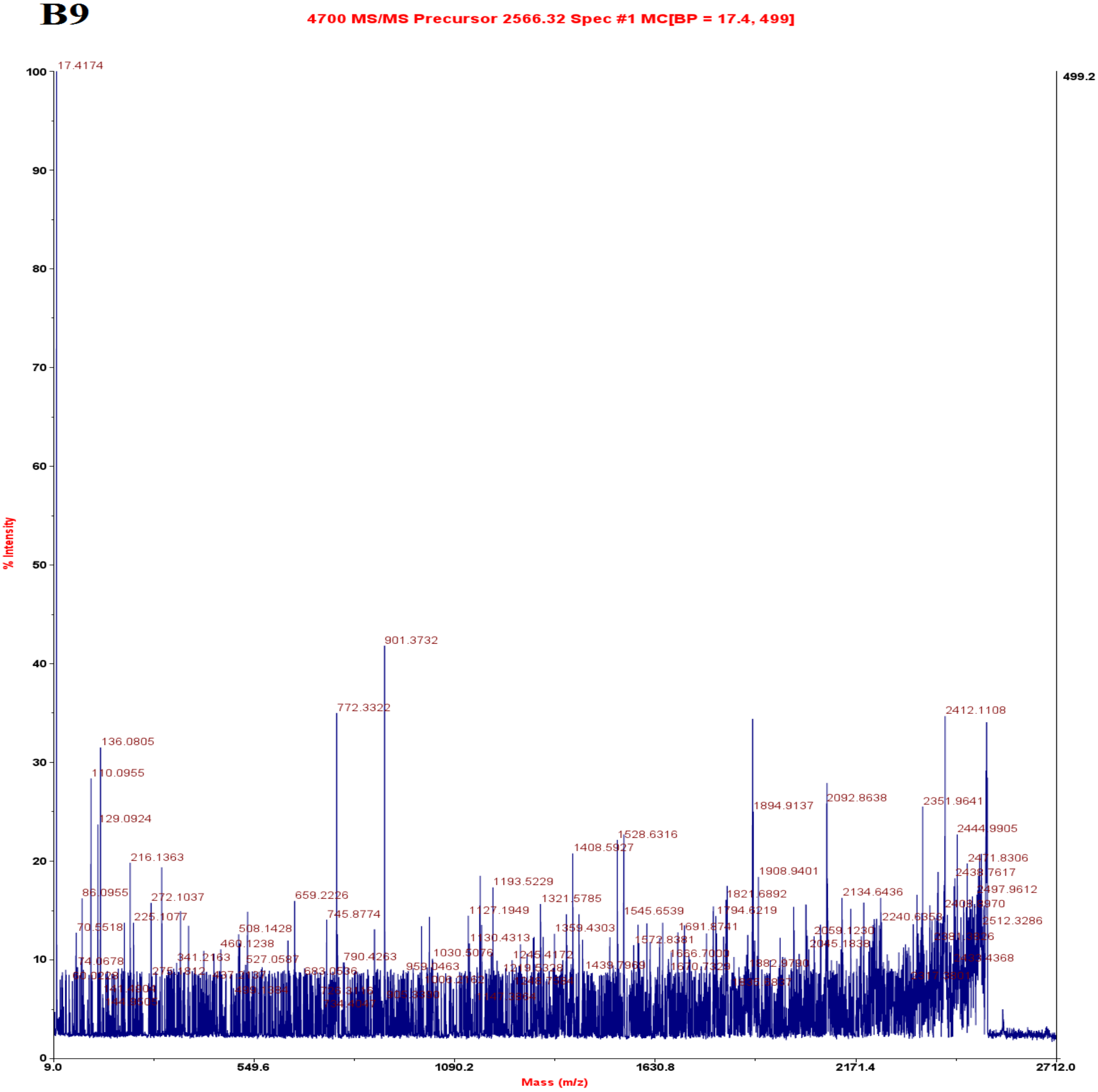
4700 MS/MS Precursor 2566.32 Spec #1 MC[BP = 17.4, 499]
% Intensity
Mass (m/z)
499.2
17.4174
136.0805
110.0955
129.0924
216.1363
86.0955
272.1037
225.1077
70.5518
74.0678
341.2163
460.1238
508.1428
527.0587
659.2226
745.8774
772.3322
790.4263
901.3732
1030.5076
1127.1949
1130.4313
1193.5229
1245.4172
1321.5785
1359.4303
1408.5927
1528.6316
1545.6539
1572.8381
1666.7000
1691.8741
1794.6219
1821.6892
1894.9137
1908.9401
2045.1838
2059.1230
2092.8638
2134.6436
2240.6358
2351.9641
2412.1108
2444.9905
2471.8306
2497.9612
2512.3286
9.0
549.6
1090.2
1630.8
2171.4
2712.0

B10

4700 MS/MS Precursor 2501.29 Spec #1 MC[BP = 2459.9, 559]

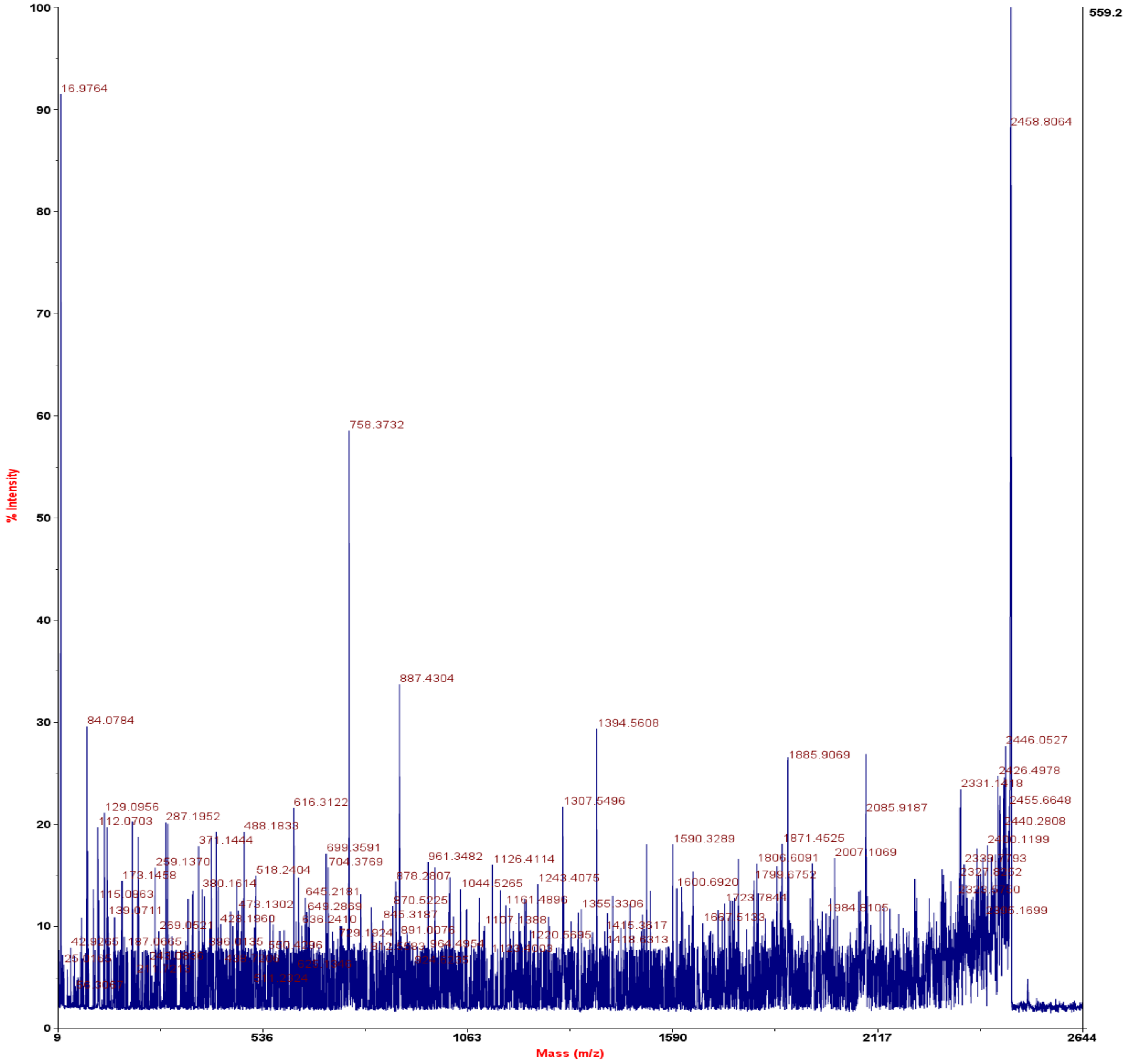

B11

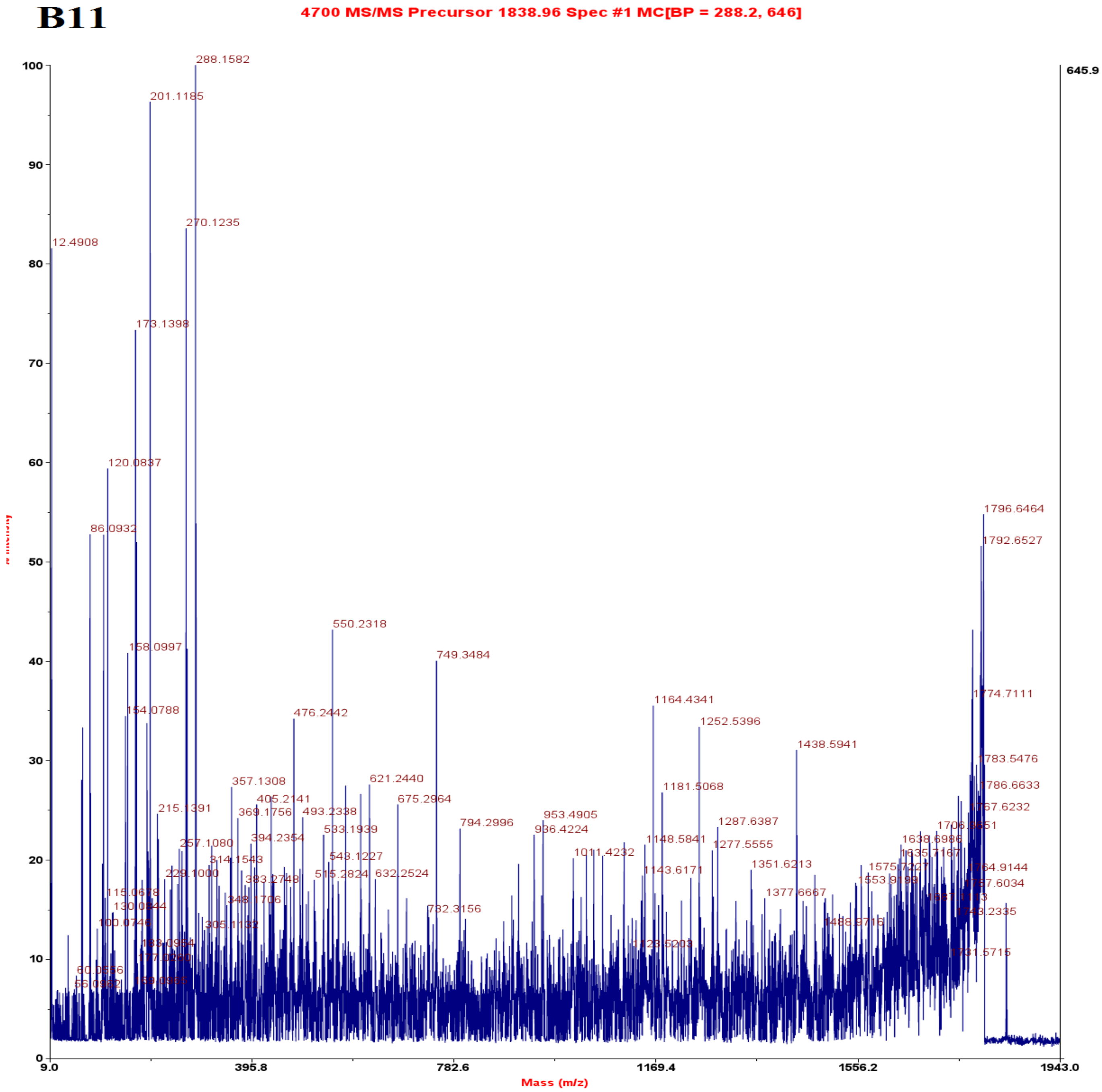
4700 MS/MS Precursor 1838.96 Spec #1 MC[BP = 288.2, 646]
645.9
Mass (m/z)
288.1582
201.1185
270.1235
12.4908
173.1398
120.0837
86.0932
1796.6464
1792.6527
550.2318
749.3484
158.0997
1164.4341
1774.7111
154.0788
476.2442
1252.5396
1438.5941
1783.5476
357.1308
621.2440
1181.5068
1786.6633
405.2141
675.2964
215.1391
369.1756
493.2338
1287.6387
953.4905
794.2996
936.4224
533.1939
257.1080
394.2354
1148.5841
1277.5555
1011.4232
314.1543
543.1227
1351.6213
229.1000
515.2824
632.2524
1143.6171
383.2748
1377.6667
115.0678
130.0844
732.3156
100.0746
305.1132
1488.8716
60.0856
1731.5715
9.0
395.8
782.6
1169.4
1556.2
1943.0

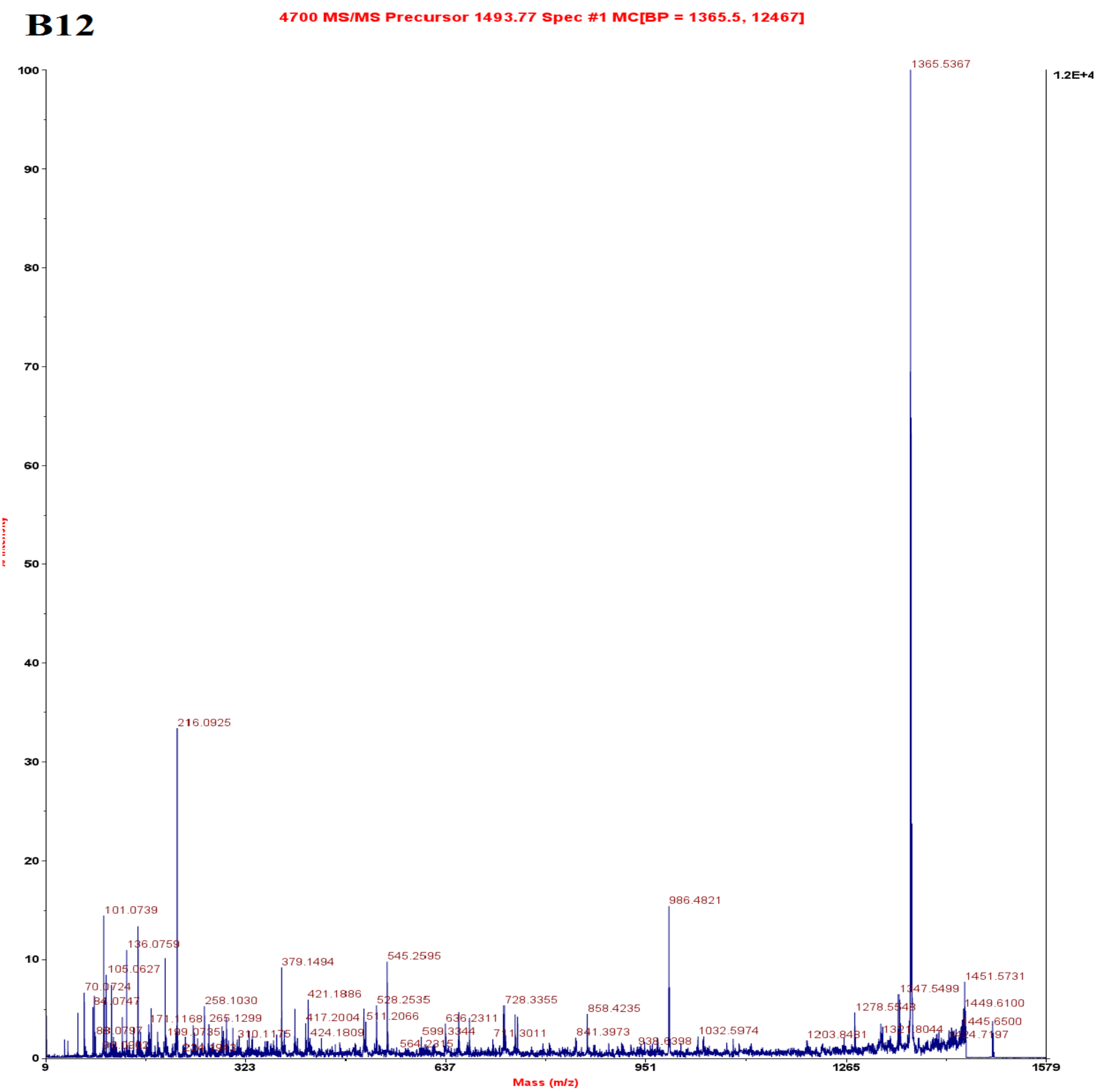

Fig. S10 Mass spectra of spot (1148) in resting cyst

A: Peptide mass fingerprinting of keratin (1148) in resting cyst; B1-B12: MS/MS spectrum of keratin (1148) in resting cyst.
