# Supplementary material for: Proteomic Approach to Reveal the Proteins Associated with Encystment of the Ciliate Euplotes encysticus
Source: PLoS One. 2014 May 16;9(5):e97362. doi: 10.1371/journal.pone.0097362 (PMC4023950; doi:10.1371/journal.pone.0097362)
Supplement: Figure S11 — Mass spectra of spot (1158) in resting cyst. A: Peptide mass fingerprinting of trimethyllysine hydroxylase (1158) in resting cyst. B: MS/MS spectrum of trimethyllysine hydroxylase (1158) in resting cyst. (PDF) [file pone.0097362.s011.pdf]

A

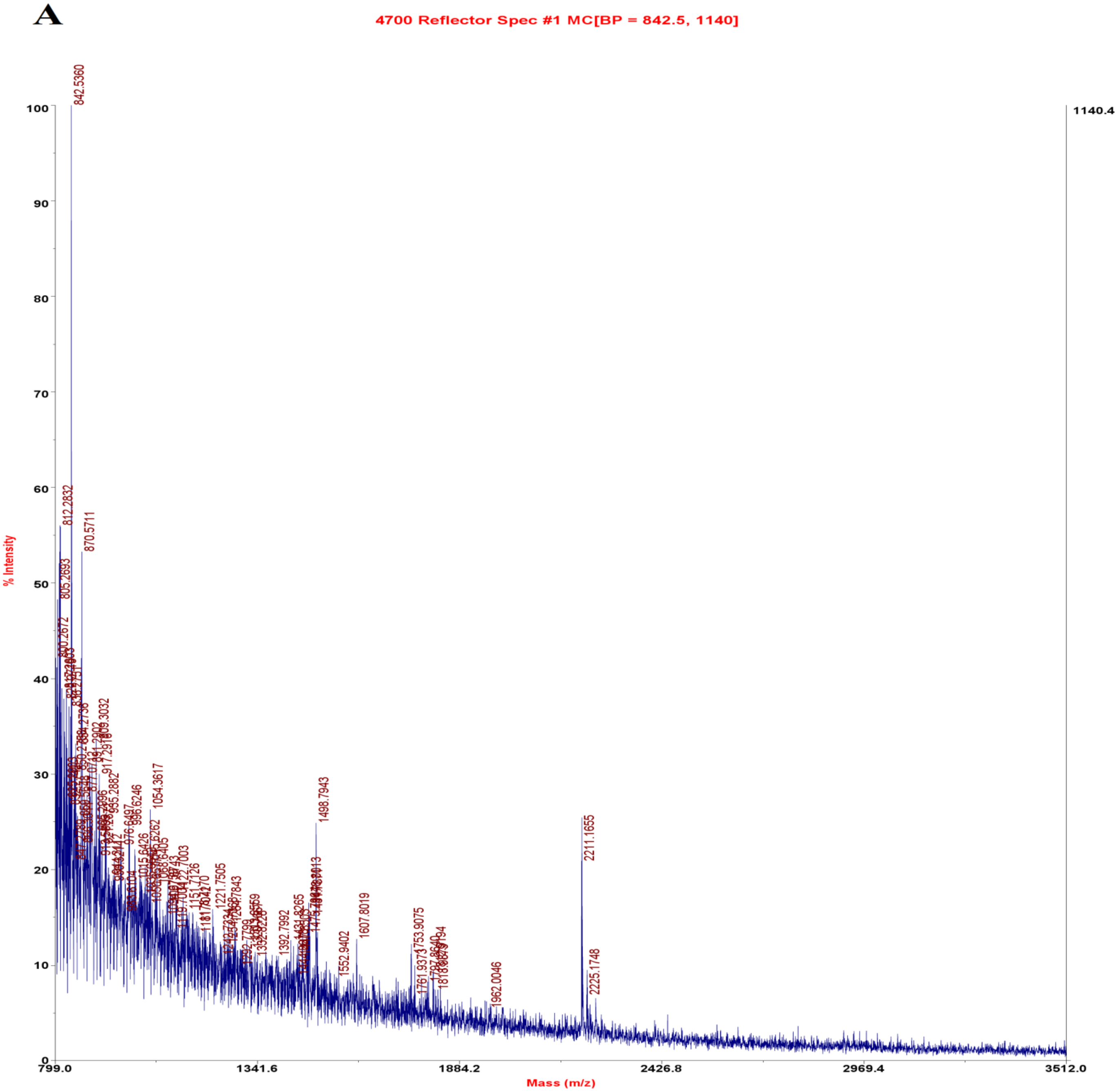
4700 Reflector Spec #1 MC[BP = 842.5, 1140]
% Intensity
Mass (m/z)
1140.4
842.5360
812.2832
870.5711
805.2693
800.2672
909.3032
917.2918
935.2882
1054.3617
996.6246
976.6497
1498.7943
2211.1655
2225.1748
1221.7505
1151.7126
1392.7992
1431.8265
1607.8019
1552.9402
1753.9075
1761.9373
1962.0046
799.0
1341.6
1884.2
2426.8
2969.4
3512.0
100
90
80
70
60
50
40
30
20
10
0

B

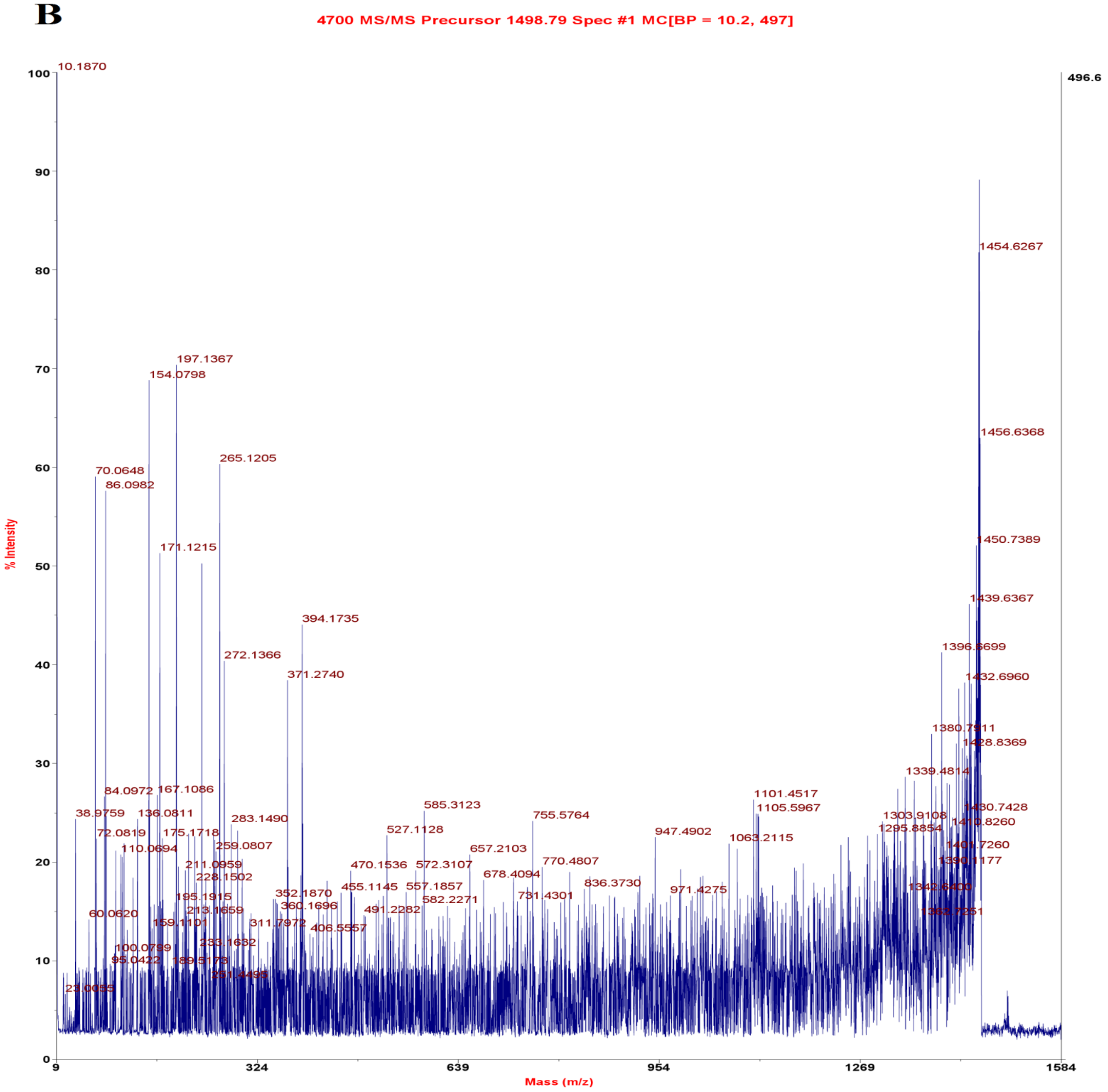

Fig. S11 Mass spectra of spot (1158) in resting cyst

A: Peptide mass fingerprinting of trimethyllysine hydroxylase (1158) in resting cyst. B: MS/MS spectrum of trimethyllysine hydroxylase (1158) in resting cyst.
